# Perioperative Outcomes in Robotic, Laparoscopic, and Open Distal Pancreatectomy: A Network Meta-Analysis and Meta-Regression

**DOI:** 10.3390/cancers17193243

**Published:** 2025-10-06

**Authors:** Nasser Abdul Halim, Eran Sadot, Ionut Negoi

**Affiliations:** 1 Rabin Medical Center, Beilinson Hospital, Medical Faculty, Tel Aviv University, Petah Tikva 4941492, Israel; 2 Clinical Emergency Hospital of Bucharest, Carol Davila University of Medicine and Pharmacy Bucharest, No. 8 Floreasca Street, Sector 1, 014461 Bucharest, Romania

**Keywords:** distal pancreatectomy, robotic surgery, laparoscopy, open approach, network meta-analysis, meta-regression

## Abstract

**Simple Summary:**

Minimally invasive techniques are increasingly used for distal pancreatectomy, yet uncertainty remains regarding the relative benefits of robotic, laparoscopic, and open approaches. Using a network meta-analysis of 67 studies and over 18,000 patients, we compared short-term perioperative outcomes across the three techniques. We found that both robotic and laparoscopic procedures reduced blood loss, transfusion rates, and hospital stay compared to open surgery. Notably, robotic surgery was associated with the lowest conversion rate and significantly reduced 30-day mortality, while laparoscopic outcomes were generally intermediate. However, readmission and 90-day major complication rates did not differ significantly, suggesting that these outcomes may be influenced more by disease-specific factors than by surgical approach. Importantly, most robotic data originated from high-volume expert centers, limiting generalizability, and long-term oncologic outcomes were not available. Overall, our findings support the perioperative safety and efficacy of minimally invasive distal pancreatectomy, while emphasizing the need for cautious interpretation of robotic advantages beyond high-volume practice.

**Abstract:**

Background: Distal pancreatectomy (DP) is a potentially curative procedure for tumors of the pancreatic body and tail. Minimally invasive DP (MIDP), including laparoscopic and robotic techniques, is increasingly being adopted. This study aimed to evaluate the perioperative outcomes of robotic DP (RDP) in comparison with laparoscopic and open approaches using a network meta-analysis and meta-regression. Methods: We systematically searched MEDLINE, EMBASE, Web of Science, and Scopus for studies comparing at least two surgical approaches. Both Bayesian and frequentist network meta-analyses were performed. Results: Sixty-seven studies involving 18,113 patients met the inclusion criteria. Surface under the cumulative ranking (SUCRA) analysis showed that RDP ranked first in 84.6% of measured parameters. Laparoscopic DP (LDP) demonstrated intermediate performance, whereas open DP (ODP) consistently ranked lowest. Operative time was significantly longer for RDP compared with ODP (MD = +25.93 min, 95% CI 7.68–44.18), while LDP and ODP were comparable. RDP significantly reduced 30-day mortality (OR = 0.37, 95% CI 0.16–0.84) and conversion rates compared with LDP (OR = 0.30, 95% CrI 0.22–0.40). Both minimally invasive approaches (RDP and LDP), compared with open surgery, were associated with reduced blood loss (−304 mL and −273 mL), fewer transfusions (OR 0.25 and 0.30), smaller transfused volumes (−1.98 and −1.86 units), shorter ICU stays (−4.0 and −2.3 days), fewer reinterventions (OR 0.45 and 0.56), and shorter hospital stays (−8.8 and −6.9 days), respectively. Conclusions: Although associated with longer operative time, RDP appears safe and may confer significant advantages over both laparoscopic and open surgery, including reduced 30-day mortality, lower conversion rates, and improved perioperative outcomes, particularly when performed in high-volume, well-equipped centers.

## 1. Introduction

The incidence of pancreatic ductal adenocarcinoma (PDAC) has increased in recent decades, ranking it as the twelfth most prevalent cancer and the sixth leading cause of cancer-related deaths globally [1]. Tumors of the pancreatic body and tail account for 20–25% of PDAC cases [2]. The widespread use of advanced imaging modalities has also led to a significant increase in the incidental finding of asymptomatic pancreatic lesions, including pancreatic neuroendocrine tumors (pNETs) and cystic neoplasms [3,4].

Distal pancreatectomy is the standard procedure for resectable lesions of the pancreatic body and tail. Since the introduction of laparoscopic distal pancreatectomy (LDP) in 1994 [5] and robotic distal pancreatectomy (RDP) in 2003 [6], there has been increasing interest in minimally invasive distal pancreatectomy (MIDP). Randomized controlled trials [7,8] and large series from high-volume centers [9,10,11] have demonstrated that MIDP offers several advantages over open distal pancreatectomy (ODP). These include reduced intraoperative bleeding, shorter hospitalization, faster postoperative recovery, and comparable oncologic outcomes [12].

Both the Miami [13] and Brescia [14] guidelines now support the use of MIDP as a standard alternative to ODP for benign, low-grade malignant, and selected malignant lesions, provided that the procedure is performed by surgeons with expertise and experience, in high-volume centers. Despite the technical advantages such as high-resolution three-dimensional visualization, tremor filtration, motion scaling, and improved ergonomics, RDP remains less commonly applied than LDP [15].

Evidence consistently confirms the benefits of MIDP over ODP [16,17,18]. However, few head-to-head comparisons exist among all three approaches. Current results are strongly influenced by heterogeneity in patient selection, surgical expertise, institutional practices, and surgical volume. Recent network meta-analyses (NMA) [19,20] have highlighted the advantages of MIDP compared with ODP but reached conflicting conclusions on the relative performance of RDP versus LDP. These inconsistencies underscore the need for an updated and comprehensive analysis to better define the role of robotic surgery in distal pancreatectomy. Network meta-analysis with meta-regression allows simultaneous comparison of open, laparoscopic, and robotic approaches, even when not directly compared within the same trial [21,22]. This method provides comprehensive ranking, clarifies relative effectiveness and safety, and offers insights that standard pairwise analyses cannot [23]. Accordingly, this study aimed to evaluate the perioperative outcomes of RDP in comparison with LDP and ODP using an NMA and meta-regression.

## 2. Methods

Network meta-analysis provides a comprehensive overview of treatment options by facilitating direct and indirect comparisons among various surgical approaches.

We compared robotic, laparoscopic, and open distal pancreatectomy. Primary and secondary perioperative outcomes were analyzed. The objective was to evaluate the perioperative outcomes of RDP in comparison with LDP and ODP.

### 2.1. Protocol and Registration

This study followed the PRISMA-NMA (Preferred Reporting Items for Systematic Reviews and Meta-Analyses incorporating Network Meta-Analyses) guidelines [24]. The study protocol was registered on the Open Science Framework (OSF; https://osf.io/ghtaz/, accessed on 15 August 2025).

### 2.2. Eligibility Criteria

We included randomized controlled trials (RCTs), prospective non-randomized comparative studies, and retrospective cohort studies. Eligible studies compared at least two of the following surgical techniques for distal pancreatectomy: open (ODP), laparoscopic (LDP), and robotic (RDP). Studies were required to report at least one perioperative outcome: patient age, sex, ASA status, history of cardiovascular comorbidities, intraoperative blood loss, quantity of transfused blood, conversion to open surgery, ICU stay, operative time, reintervention, readmission, in-hospital or 30-day mortality, 90-day major complications, or postoperative hospital stay length. Exclusion criteria were: (1) non-comparative studies; (2) case reports or case series with fewer than 10 patients; (3) studies on central or total pancreatectomy; (4) animal studies; and (5) abstracts without full-text availability.

### 2.3. Information Sources and Search Strategy

A systematic search was conducted in MEDLINE (via PubMed), EMBASE, Web of Science, and Scopus from inception to December 2024. The search included MeSH terms and keywords such as “distal pancreatectomy”, “left pancreatectomy”, “robotic”, “laparoscopic”, and “minimally invasive”. Manual searches of the references of relevant studies and systematic reviews were also performed.

### 2.4. Study Selection

All search results were imported into Paperpile and screened. Duplicates were removed. Titles and abstracts were screened for eligibility. The full texts of potentially eligible studies were assessed independently. Disagreements were resolved through discussion or consultation with a third reviewer.

### 2.5. Data Collection Process and Data Items

Data were extracted using a standardized collection form. Extracted variables included the first author, publication year, country of origin, study methodology, number of patients in each arm, surgical approach (open, laparoscopic, robotic), patient demographics (age, sex), ASA status, comorbidities, and all predefined perioperative outcomes. When available, we recorded means with standard deviations, medians with interquartile ranges, and event counts with sample sizes.

### 2.6. Risk of Bias Assessment

For non-randomized studies, the Newcastle–Ottawa Scale (NOS) was applied. Studies scoring 6 and above were selected. For prospective randomized controlled trials (RCTs), the Cochrane Risk of Bias 2.0 tool was used. Discrepancies were resolved through consensus. Results are summarized in Table 1.

### 2.7. Outcome Measures

Seventeen predefined perioperative outcomes were analyzed in this study. Continuous outcomes (e.g., operative time, blood loss, and hospital stay) were expressed as mean differences (MD), whereas dichotomous outcomes (e.g., mortality and reintervention) were reported using odds ratios (OR). Each outcome was pooled using both frequentist and Bayesian network meta-analyses.

### 2.8. Geometry of the Network

Network plots were constructed for each outcome to assess the structure of the available comparisons across the interventions. The number of direct and indirect comparisons per network was recorded.

### 2.9. Summary Measures and Statistical Analysis

Frequentist NMA was performed using the netmeta package in R (version 4.3.0), applying a random-effects model based on the DerSimonian–Laird method for variance estimation. For Bayesian NMA, we used the gemtc, BUGSnet, and bnma packages with Markov Chain Monte Carlo (MCMC) simulations. Three chains were run for 10,000 iterations after a burn-in of 5000 iterations.

Treatment rankings (Rank 1 = best, Rank 2 = second best, Rank 3 = worst) were evaluated using the Surface Under the Cumulative Ranking curve (SUCRA). To better visualize the results, we used Litmus Rank-O-Gram and radial SUCRA plots [91]. To score the overall treatment performances, we converted the ranks to scores (Rank 1 = 3 points, Rank 2 = 2 points, Rank 3 = 1 point) and created a comprehensive scoring chart showing total performance scores and category breakdowns.

Network inconsistency was assessed using global (design-by-treatment interaction) and local (node-splitting) approaches. The consistency between direct and indirect evidence was evaluated, being declared when *p* < 0.05 [92].

Model fit was evaluated by comparing the deviance information criterion (DIC), residual deviance, and leverage plots. We investigated the deviance of the model using the residual deviance from the NMA model, the unrelated mean effect (UME) inconsistency model, per-arm residual deviance, and the leverage plot for all studies [93].

Meta-regression was conducted using study publication year and sample size as covariates to explore sources of heterogeneity [94].

### 2.10. Assessment of Inconsistency and Small-Study Effects

Global tests and node-splitting models were utilized to investigate the inconsistency between direct and indirect comparisons. Funnel and comparison-adjusted funnel plots were generated to assess potential publication bias and small-study effects.

### 2.11. Additional Analyses

Prespecified sensitivity analyses included (1) restricting studies to those with a low risk of bias and (2) excluding outliers. Subgroup analyses were conducted based on publication year and surgical volume.

### 2.12. Software

Statistical analyses were performed using R environment (version 4.3.0) [95]. For frequentist NMA, we used the R package netmeta [96], while for Bayesian NMA, we used the R packages gemtc [97], BUGSNET, and bnma [95,98,99,100]. For graphical representation, Python 3.13 was also used.

## 3. Results

### 3.1. Search Results

**Figure 1:**
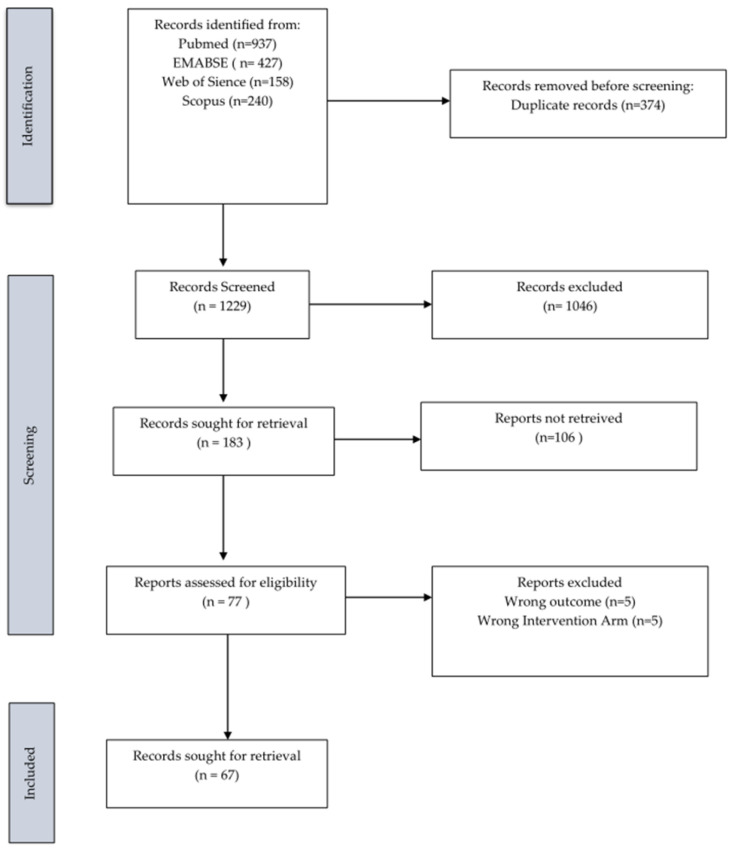
PRISMA flow diagram for the systematic review and meta-analysis.

A total of 937 studies were identified, with 67 meeting the inclusion criteria (Figure 1) [24].

### 3.2. Characteristics of the Included Studies

We included 67 studies in the NMA. The baseline characteristics are presented in Table 2.

### 3.3. Outcomes NMA

We analyzed 17 parameters comprising preoperative, intraoperative, and short-term postoperative results. The outcomes are summarized in Table 3.

### 3.4. Age and Sex of Patients

Across 59 studies, including 17,542 patients, the mean age was 59.5 years (Supplementary Table S1 and Figure S1). The network comprised fifty-two two-arm and seven multi-arm studies. A total of 73 pairwise comparisons were available. Specifically, eight trials compared ODP vs. RDP, twenty-nine trials ODP vs. LDP, and thirty-six trials LDP vs. RDP (Supplementary Figure S2).

In the frequentist NMA, patients in the RDP group were significantly younger than those in the ODP group, with a mean difference (MD) of −1.65 years (95% confidence interval [CI] −3.00 to −0.30). No significant age difference was observed between LDP and ODP (MD = −0.72, 95% CI −1.86 to 0.41) or between RDP and LDP (MD = −0.93, 95% CI −1.93 to 0.07) (Supplementary Figures S3 and S4 and Supplementary Table S1). No significant inconsistency was detected between the direct and indirect comparisons (Supplementary Table S2). In the Bayesian NMA, patients in the RDP group were significantly younger than those in the ODP group (MD = −1.67 years, 95% CI −3.11 to −0.26) (Supplementary Figure S5 and Supplementary Table S3). No significant differences in age were found between the RDP and LDP (MD = −0.93, 95% CrI −1.97 to 0.11) or between LDP and ODP (MD = −0.74, 95% CrI −1.95 to 0.43) (Supplementary Figures S6 and S7).

The model showed no evidence of lack of fit (no significant residual deviance) (Supplementary Figures S8–S10). Meta-regression indicated that patients in the ODP group were older in earlier studies (Supplementary Figure S11), with the age difference between groups diminishing in more recent years (i.e., no age-selection difference in the most recent studies) (Supplementary Figure S12).

The analysis of sex distribution across groups is presented in the Supplementary Materials, page 28.

### 3.5. ASA Status

Forty studies (54 pairwise comparisons; 10,318 patients) reported ASA class I–II distribution (Supplementary Figures S18 and S19).

No statistically significant differences were observed between the RDP, LDP, and ODP in the proportion of patients classified as ASA I–II (Supplementary Figure S20 and Supplementary Table S6). SUCRA rankings were very similar among the three groups, with no significant separation (Supplementary Figures S21 and S22). No inconsistencies were observed between the direct and indirect comparisons (Supplementary Figure S23).

Meta-regression with the year of study as a covariate showed no significant change in the relative ASA distribution over time (Supplementary Figure S24).

### 3.6. Previous Cardiovascular Diseases

Twelve studies (18 pairwise comparisons; 3307 patients) reported pre-existing cardiovascular comorbidities (Supplementary Figures S25 and S26). There were no significant differences between the surgical approaches in terms of the proportion of patients with a history of cardiovascular disease (Supplementary Figure S27 and Supplementary Table S7). Baseline cardiovascular comorbidity rates were therefore comparable across groups (Supplementary Figure S28). This suggests that patient selection in terms of cardiovascular health was similar across the groups. No inconsistency was detected (Supplementary Table S8).

Bayesian NMA results were similar to those of the frequentist analysis (Supplementary Figures S29–S32 and Supplementary Table S9). No deviance was observed between the studies (Supplementary Figures S33–S35).

Meta-regression showed that differences in cardiovascular comorbidities decreased over time (Supplementary Figure S36).

### 3.7. Operative Time

Operative time was reported in 61 studies (79 pairwise comparisons, including 16,230 patients) (Supplementary Figures S37 and S38). In the frequentist NMA, RDP was associated with a significantly longer operative time than ODP (MD = +25.93 min, 95% CI 7.68–44.18) (Supplementary Figure S39 and Supplementary Table S10). LDP had a slight mean increase in operative time compared to ODP, without statistical significance (MD = +7.63 min, 95% CI −7.59 to +22.85). No inconsistency was detected (Supplementary Table S11). SUCRA rankings identified ODP as the fastest (Rank 1), followed by LDP (Rank 2) and RDP (Rank 3) (Supplementary Figure S40). In the Bayesian NMA, findings were consistent. RDP lasted, on average, 27.2 min longer than ODP (95% CrI 4.3–50.3) (Supplementary Figure S41 and Supplementary Table S12). Differences between LDP and ODP remained small and non-significant. Approach rankings matched the frequentist results (ODP fastest, followed by LDP and RDP) (Supplementary Figures S42 and S43). No inconsistency was detected (Supplementary Figures S44–S47).

Meta-regression analysis revealed no significant temporal trends and no effect of study sample size on operative time differences (Supplementary Figures S48 and S49). Overall, RDP was associated with longer operative time than ODP or LDP, with LDP and ODP showing comparable durations.

### 3.8. Conversion to Open

**Figure 2:**
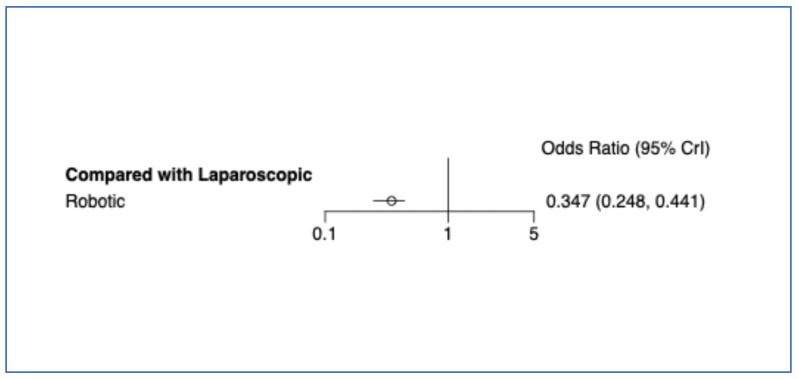
Bayesian random effect consistency model forest plot for the outcome conversion rate.

**Figure 3:**
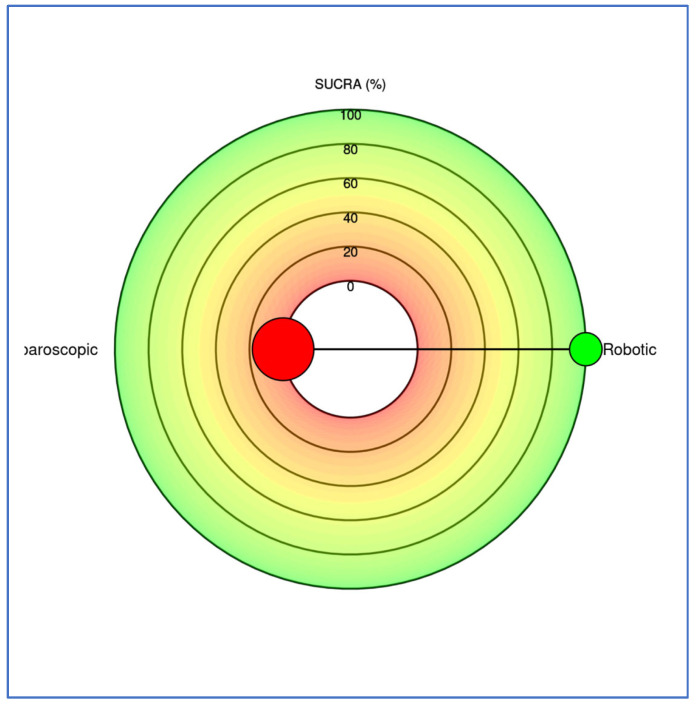
Radial SUCRA plot: Outcome conversions. Higher SUCRA values indicate better treatments; the size of the nodes represents the number of participants, and the thickness of the lines indicates the number of trials conducted. Green circle represents robotic approach. Red circle represents laparoscopic approach.

Thirty-eight studies (10,586 patients) reported conversion to open surgery among RDP and LDP (Supplementary Figures S50 and S51). In the NMA, RDP was associated with a significantly lower conversion rate than LDP. The odds of conversion were 65.3% lower with RDP compared to LDP (OR = 0.347, 95% CI 0.248–0.441 for RDP vs. LDP) (Figure 2). Conversely, the odds of conversion were approximately 2.8 times higher with LDP compared to RDP (OR = 2.88 for LDP vs. RDP, 95% CI 2.27–4.03) (Supplementary Table S14). Based on SUCRA, RDP ranked first, with the lowest conversion rate (Figure 3).

**Figure 4:**
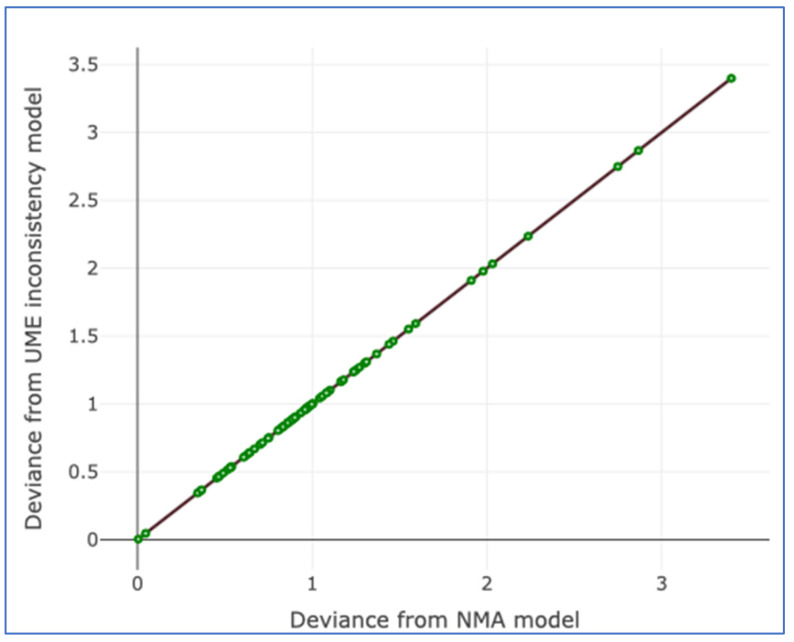
Residual deviance from the NMA model and the UME inconsistency model for all studies.

**Figure 5:**
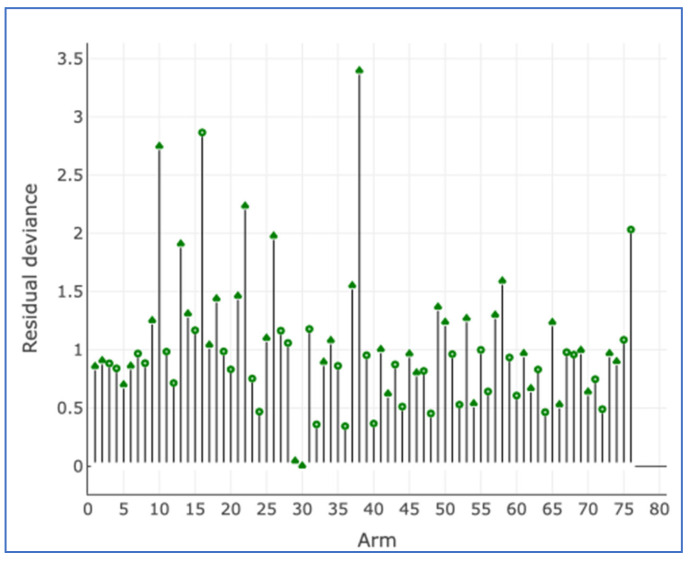
Per-arm residual deviance for all studies. This stem plot represents the posterior residual deviance per study arm.

**Figure 6:**
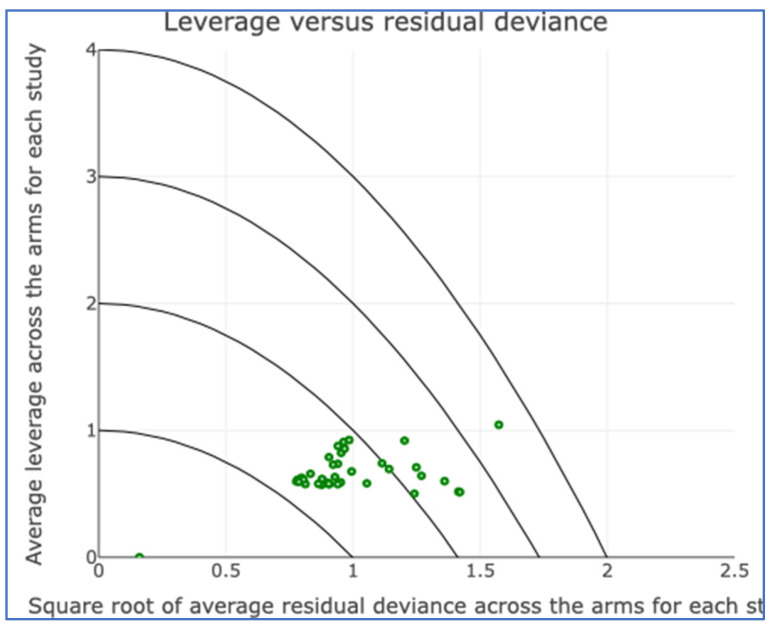
Leverage plot for all studies.

There was no evidence of inconsistency in this two-treatment network (Figure 4, Figure 5 and Figure 6).

**Figure 7:**
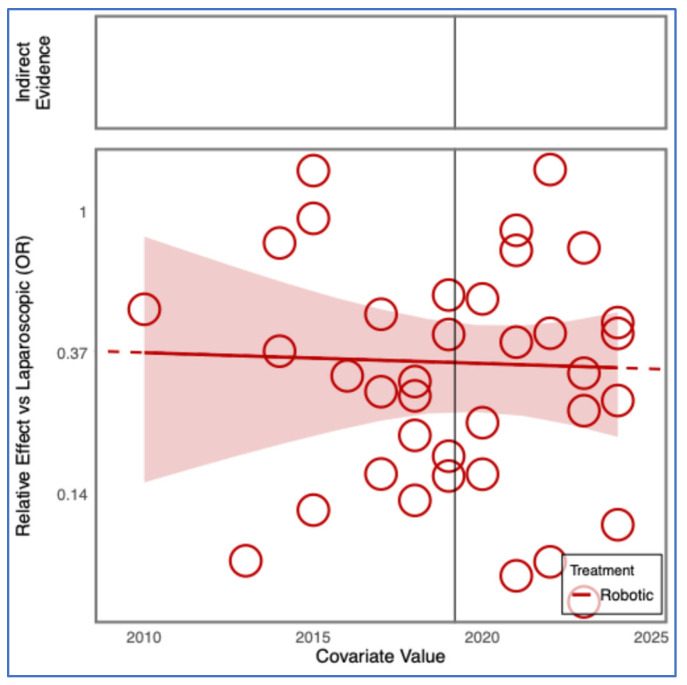
Regression plot for the outcome conversions, for robotic versus laparoscopic approaches, having as a covariate the year of study publication. OR = odds ratio.

**Figure 8:**
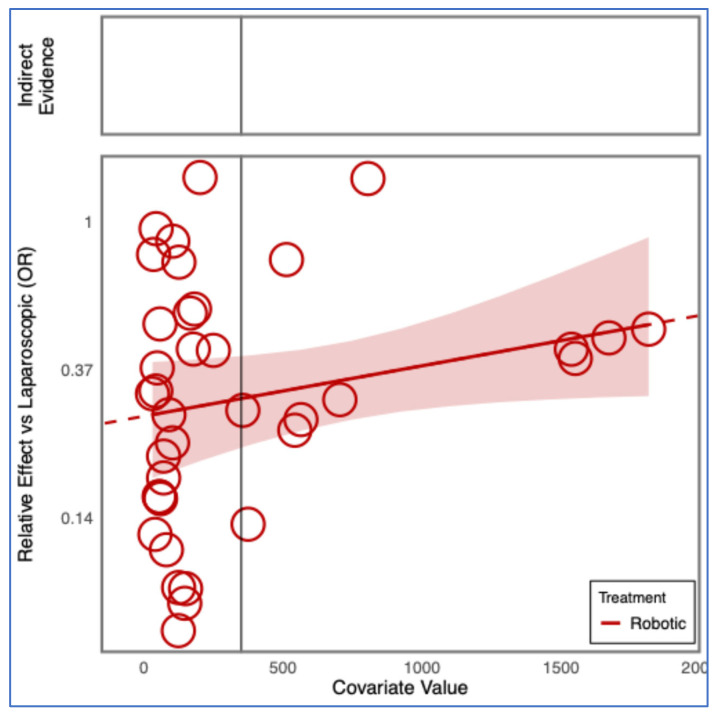
Regression plot for the outcome conversions, having as a covariate the number of patients in the included studies.

Frequentist and Bayesian analyses confirmed a significantly lower conversion risk for RDP compared with LDP (Supplementary Figure S52). Meta-regression did not show any significant effect of publication year or study size on conversion rates. The advantage of RDP over LDP remained consistent across study timelines and sample sizes (Figure 7 and Figure 8).

### 3.9. Intraoperative Blood Loss

A total of fifty-one studies (forty-six two-arm and five multi-arm) reported intraoperative blood loss, including 12,257 patients in the network (Supplementary Figure S60). There were 61 pairwise comparisons (Supplementary Figure S61).

In the frequentist NMA, both RDP and LDP were associated with significantly lower blood loss than ODP. The mean difference was −279.45 mL (95% CI −318.23 to −240.61) for RDP vs. ODP, and −248.99 mL (95% CI −283.41 to −214.57) for LDP vs. ODP. No significant differences were observed between RDP and LDP in the frequentist analysis (Supplementary Figure S62, Supplementary Table S15). No inconsistencies were detected in the network (Supplementary Table S16). SUCRA rankings ordered the approaches from lowest to highest blood loss as follows: RDP, LDP, and ODP (Supplementary Figure S63). In the Bayesian NMA, results were consistent. RDP and LDP showed significantly lower blood loss than ODP (MD = −303.98 mL, 95% CrI −382.8 to −229.26 for RDP; MD = −272.89 mL, 95% CrI −340.15 to −209.01 for LDP) (Supplementary Figures S64–S66, Supplementary Table S17). There was no notable model deviance or lack of convergence in the Bayesian analysis (Supplementary Figures S68–S70).

Meta-regression indicated that the difference in blood loss between open and minimally invasive approaches had narrowed in more recent studies compared with older (>10 years) studies (Supplementary Figures S71–S73). In contrast, sample size did not significantly affect results, although larger studies tended to report greater blood loss reduction with RDP and LDP compared to ODP (Supplementary Figure S74).

### 3.10. Intraoperative Bleeding More than 500 mL

Three studies (1427 patients) reported the number of patients experiencing intraoperative blood loss exceeding 500 mL (Supplementary Figure S76). LDP showed a significantly lower incidence of ≥500 mL blood loss than the ODP (OR = 0.32, 95% CI 0.23–0.46) (Supplementary Figure S77 and Supplementary Table S18). For RDP, the risk was also lower than ODP (OR = 0.11, 95% CI 0.01–1.07); however, this difference was not statistically significant due to wide confidence intervals, likely reflecting the small number of events in the robotic arm. No studies directly compared RDP and LDP for this outcome. However, point estimates suggested that both modalities reduced the risk of major bleeding relative to ODP. No inconsistencies were detected (Supplementary Table S19). These findings indicate that MIDP approaches tend to reduce the risk of excessive intraoperative bleeding, with laparoscopy showing a significant reduction and robotics showing a similar trend.

### 3.11. Number of Patients Receiving Transfusions

Thirty studies (38 pairwise comparisons, with 9248 patients) reported intraoperative or postoperative transfusion events (Supplementary Figures S78 and S79). Both minimally invasive approaches were associated with significantly lower transfusion rates compared to ODP. The odds of requiring transfusion were markedly reduced for RDP vs. ODP (OR = 0.25, 95% CI 0.19–0.34) and for LDP vs. ODP (OR = 0.30, 95% CI 0.24–0.37) (Supplementary Figure S80, Supplementary Table S20). There was no significant difference in the transfusion rate between RDP and LDP. No inconsistency was detected in the network (Supplementary Table S21). SUCRA rankings identified RDP as the best approach (Rank 1), followed by LDP (Rank 2) and ODP (Rank 3) (Supplementary Figure S81).

### 3.12. The Quantity of Blood Transfusion

Four studies (six pairwise comparisons, 363 patients) reported the mean number of blood units transfused per patient (Supplementary Figures S82 and S83).

Both minimally invasive approaches were associated with a lower volume of blood transfusion compared with ODP. The mean difference was −1.98 units (95% CI −3.42 to −0.54) for RDP vs. ODP and −1.86 units (95% CI −3.12 to −0.59) for LDP vs. ODP. On average, patients undergoing MIDP received approximately two fewer units of blood than those undergoing ODP (Supplementary Figure S84, Supplementary Table S22). No significant difference was observed between RDP and LDP. No inconsistencies were detected (Supplementary Table S23).

### 3.13. Intensive Care Unit Length of Stay

Nine studies (1272 patients) reported the postoperative ICU length of stay (Supplementary Figures S85 and S86). Both RDP and LDP were associated with a significantly shorter ICU stay duration than ODP. The mean difference was −4.01 days (95% CI −5.97 to −2.05) for RDP vs. ODP and −2.27 days (95% CI −3.71 to −0.83) for LDP vs. ODP (Supplementary Figure S87 and Supplementary Table S24). On average, patients undergoing RDP spent approximately four fewer days in the ICU than ODP patients, while LDP patients spent approximately two fewer days. No significant difference was observed between RDP and LDP. No inconsistencies were detected (Supplementary Table S25). SUCRA rankings identified RDP as the best approach, followed by LDP and ODP as the worst (Rank 3) (Supplementary Figure S88). These results suggest that MIDP reduces the need for prolonged critical care, reflecting lower perioperative stress and complications.

### 3.14. Reintervention Rate

Thirty-seven studies (11,568 patients) reported postoperative reintervention rates. Both RDP and LDP were associated with lower reintervention rates compared to ODP (RDP: OR = 0.451, 95% CrI 0.225–0.836; LDP: OR = 0.560, 95% CrI 0.322–0.962) (Supplementary Table S26).

No significant difference was found between RDP and LDP.

SUCRA rankings placed RDP as the best approach (Rank 1, lowest reintervention risk), LDP as Rank 2, and ODP as the worst (Rank 3) (Supplementary Figure S92). No inconsistency was detected (node split analysis, *p* > 0.05; Supplementary Figure S93).

### 3.15. Hospital Length of Stay

Sixty-three studies (18,113 patients; fifty-four two-arm and nine multi-arm studies) reported postoperative hospital stay (Supplementary Figures S97 and S98). Both minimally invasive approaches showed significantly shorter hospital stays than ODP (Supplementary Figure S101). Pooled estimates indicated that RDP patients had a shorter length of in-hospital stay than ODP, with a MD of −8.77 days (95% CrI −13.34 to −4.22), while LDP had an MD of −6.93 days (95% CrI −10.67 to −3.23) (Supplementary Table S29).

No inconsistencies were detected (Supplementary Figure S104). SUCRA rankings placed RDP as the best approach (shortest stay), LDP as second, and ODP as the worst (Supplementary Figures S102 and S103). Meta-regression showed no temporal effect; the advantage of minimally invasive approaches remained stable over time. These results confirm that MIDP, particularly RDP, facilitates faster recovery and earlier discharge compared with ODP.

### 3.16. Readmission Rate

Thirty-one studies (34 comparisons) reported 30-day readmission rates (Supplementary Figures S110 and S111). No statistically significant differences were observed among the three approaches (Supplementary Figure S112, Supplementary Table S30). No inconsistency was detected (Supplementary Table S31). SUCRA values were nearly identical across the three approaches, indicating no clear ranking advantage (Supplementary Figure S113). These findings suggest that the choice of surgical approach does not significantly affect early hospital readmission.

### 3.17. In-Hospital Mortality

Nine studies (1009 patients) reported in-hospital (same-admission) mortality (Supplementary Figures S114 and S115). No significant differences were observed among ODP, LDP, and RDP (Supplementary Figures S117 and S119).

### 3.18. 30-Day Mortality

Thirty-one studies, including 12,127 patients, reported 30-day postoperative mortality (Supplementary Figures S125 and S126).

**Figure 9:**
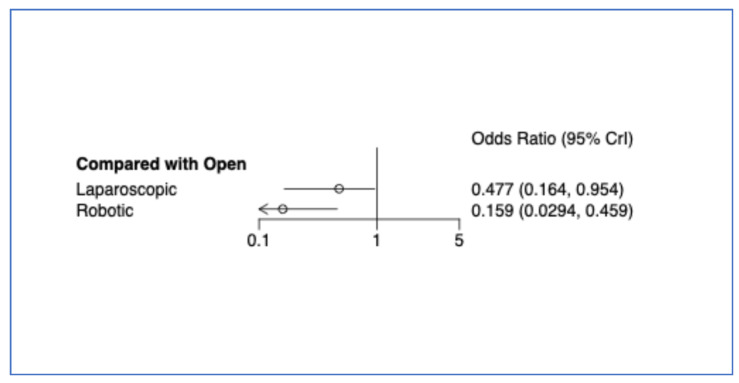
Bayesian random effect consistency model forest plot for outcome 30-day mortality. Between-study standard deviation (log-odds scale): 0.62; 95% credible interval: 0.02, 1.86.

RDP was associated with significantly lower 30-day mortality (OR = 0.159, 95% CrI 0.0294–0.459) (Figure 8). LDP also showed a reduction, although the statistical significance was marginal (OR = 0.477, 95% CrI 0.164–0.954) (Figure 9, Table 4).

**Figure 10:**
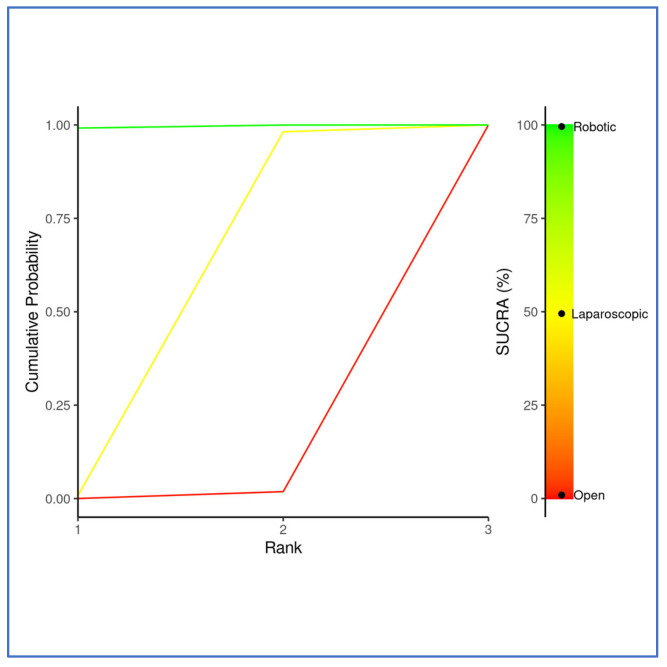
Litmus Rank-O-Gram. Outcome: 30-day mortality. Higher SUCRA (surface under the cumulative ranking curve) values and cumulative ranking curves nearer the top left indicate better performance.

**Figure 11:**
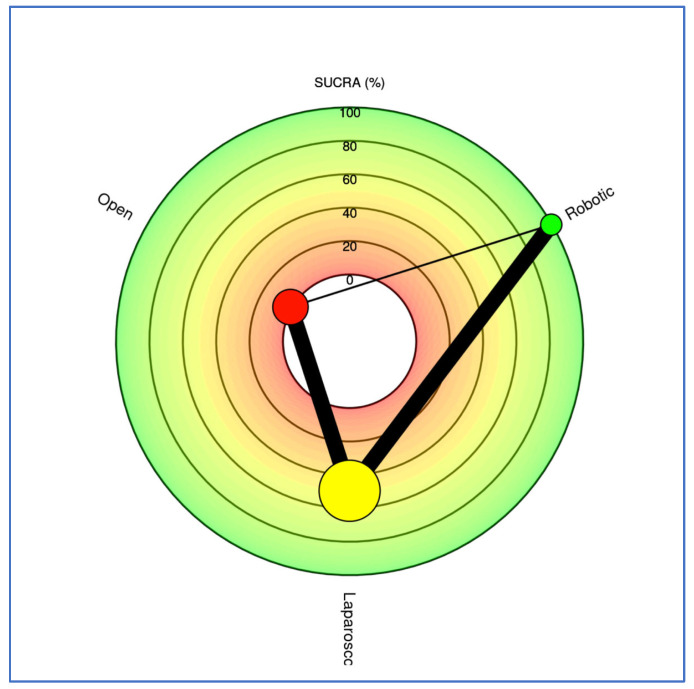
Radial SUCRA plot. Outcome: 30-day mortality. Higher SUCRA values indicate better treatments; the size of the nodes represents the number of participants, and the thickness of the lines indicates the number of trials conducted. Green circle represents robotic approach. Yellow circle represents laparoscopic approach. Red circle represents open approach.

SUCRA rankings placed RDP as the best approach, followed by LDP, and ODP third (Figure 10 and Figure 11). In the frequentist NMA, the survival benefit of LDP was attenuated and did not reach significance (Supplementary Figures S127–S130 and Supplementary Table S32).

No inconsistencies were detected between direct and indirect comparisons (Supplementary Figures S131 and S132). No deviance was observed (Supplementary Figures S133–S135).

No evidence of publication bias was observed (Supplementary Figure S140).

### 3.19. 90-Day Major Complications

Three studies (1427 patients) reported 90-day major complications (typically Clavien–Dindo grade III or higher) (Supplementary Figures S141 and S142). No significant differences were observed among the three approaches (Supplementary Figure S145). Pooled estimates for RDP vs. ODP and LDP vs. ODP showed odds ratios close to 1, with wide Crls reflecting limited data. The comparison between RDP and LDP was also non-significant (Supplementary Table S37).

## 4. Summary

**Figure 12:**
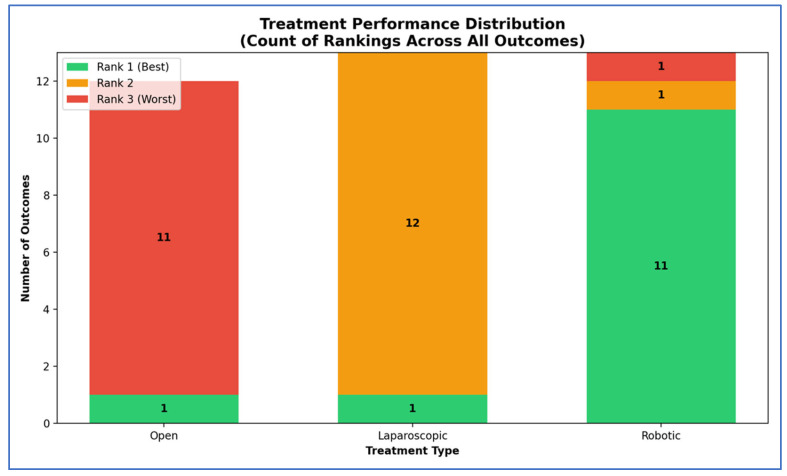
Stacked bar chart of treatment performance distribution.

**Figure 13:**
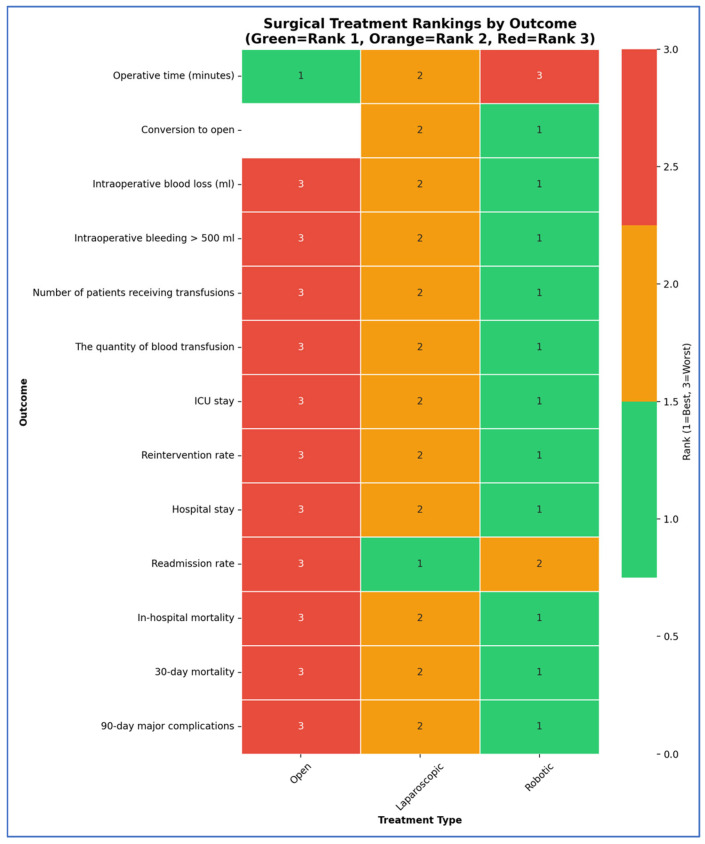
Treatment performance heatmap.

**Figure 14:**
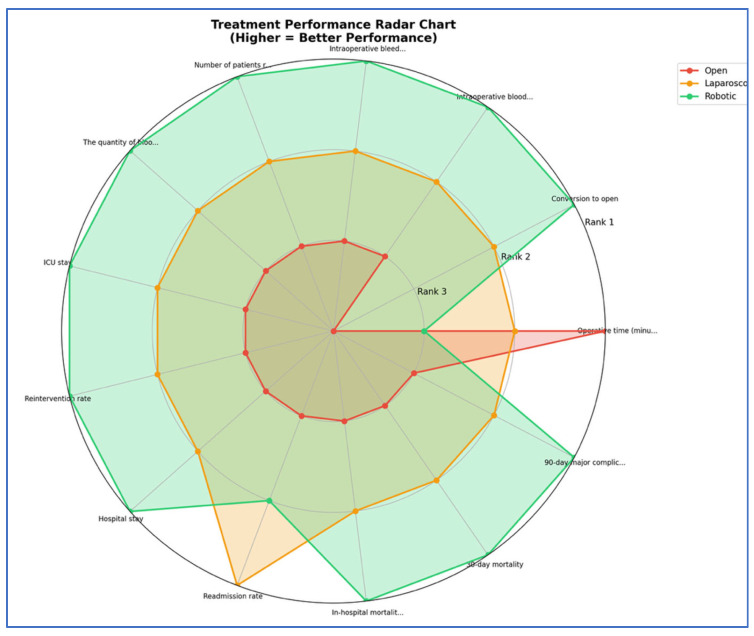
Radar chart—treatment performance comparison.

**Figure 15:**
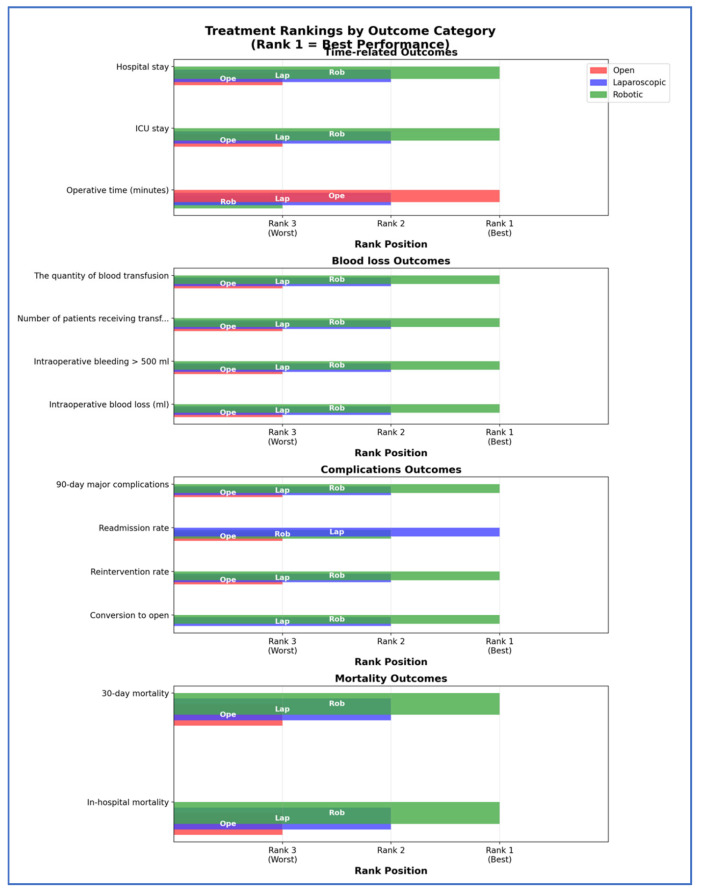
Horizontal bar chart by outcome categories.

**Figure 16:**
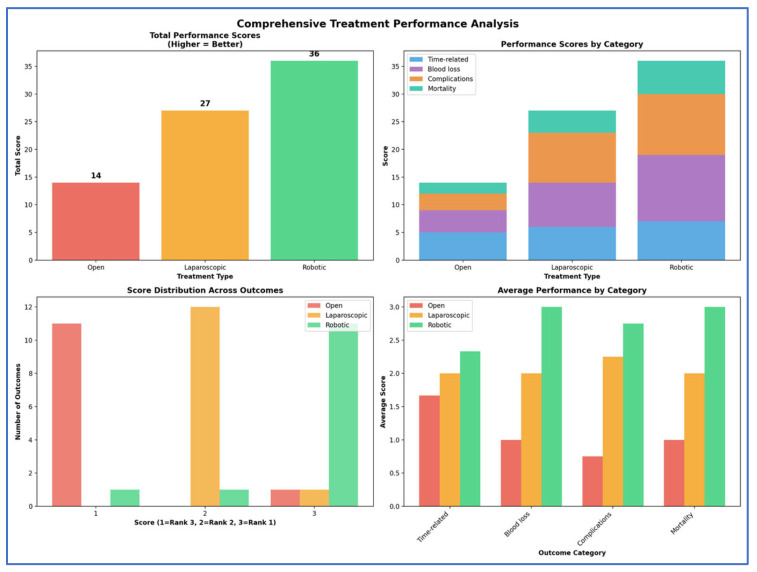
Comprehensive performance analysis. To score the overall treatment performances, we converted the ranks to scores (Rank 1 = 3 points, Rank 2 = 2 points, and Rank 3 = 1 point).

Our SUCRA ranking analysis demonstrates that robotic surgery consistently outperformed the other approaches, achieving the top rank in 84.6% of outcomes and capturing 92.3% of the maximum possible score (Figure 12, Figure 13, Figure 14, Figure 15 and Figure 16). Laparoscopic surgery showed intermediate performance, while open surgery ranked lowest across comparisons.

## 5. Discussions

This network meta-analysis provides an updated and comprehensive comparison of perioperative outcomes among robotic, laparoscopic and open distal pancreatectomy, aiming to clarify the role of robotic distal pancreatectomy (RDP) in treating resectable left pancreatic lesions.

Based on SUCRA rankings, robotic surgery demonstrated favorable performance across most evaluated outcomes, achieving the highest rank in 84.6% of assessed parameters and accounting for 92.3% of the maximum possible performance score. Specifically, the robotic approach was associated with reduced blood loss, fewer transfusions, and lower volumes of transfused blood products. These intraoperative advantages were reflected in shorter ICU and hospital stays, fewer reinterventions, and lower rates of 30-day mortality and 90-day major complications. Laparoscopy generally showed intermediate performance, while the open approach consistently ranked lowest in this comparative analysis.

The mean age of patients in the RDP group was significantly lower than that of the ODP group but not significantly different from the LDP group. However, these age differences have diminished in more recent publications compared to earlier ones. In a previous meta-analysis, patients undergoing RDP were significantly younger than those undergoing LDP [101]. The current findings suggest that with increased familiarity and broader implementation of the robotic platform in abdominal surgeries in general [102], as well as pancreatic surgery in particular [103], the indications for RDP have expanded to include older patients with more comorbidities.

Operative time was significantly longer for RDP, consistent with reports from high-volume centers [86]. This may be attributed to docking and instrument exchange, as well as the lack of tactile sensation, which often leads to more cautious maneuvers, particularly early in the learning curve. Our meta-regression analysis showed no significant temporal or sample-size-related trends in operative time, suggesting that this difference persists across settings. Three studies in our analysis compared all three techniques head-to-head [25,52,59], consistently reporting the longest operative time in the RDP and the shortest in the ODP group. However, prior meta-analysis [104] found comparable operative times across the three techniques, underlining the variability in reported experience. Operative time is inherently influenced by surgical experience and standardization of the technique [105]. Large multicenter analyses demonstrate a marked reduction in operative time in RDP as surgeons and institutions progress along the learning curve. Marked reductions are typically seen after 18–46 cases, with a plateau reached after 43–73 cases, depending on the definition of proficiency [106,107,108]. This is attributed to increased familiarity with robotic systems, improved technical efficiency, and optimized team coordination. In the present analysis, results from expert centers were pooled with those from centers still early in the learning curve. From this perspective, the open approach ranked highest in the SUCRA scores, likely reflecting the broader experience of surgeons with open surgery.

The intraoperative blood loss was significantly lower in MIDP compared to the open approach. Within MIDP, RDP showed lower blood loss than LDP, though this difference did not reach statistical significance. Intraoperative bleeding more than 500 mL was reported in only three studies, limiting the strength of the available evidence.

The conversion rate was significantly lower for RDP than for LDP, and this advantage persisted across study periods and sample sizes in meta-regression. This is a notable, given that laparoscopy is more widely adopted than robotics. The predominance of high-volume centers in RDP publications likely contributes to these favorable results.

Postoperative outcomes similarly favored minimally invasive approaches over the open approach. Hospital stay was shortest with RDP. Overall, minimally invasive procedures were associated with lower mortality than ODP. However, no significant differences were observed in readmission or 90-day major complication rates. This may indicate that these outcomes are more strongly influenced by organ-specific complications rather than the surgical approach. It is important to acknowledge that 90-day major complications were reported in only a limited number of studies (n = 3). Although these studies included a total of 1427 patients, the results should be interpreted with caution, and the conclusions must be considered preliminary. Major postoperative complications within 30 or 90 days are associated with increased recurrence and negatively impact both disease-free and overall survival [109,110]. Postoperative morbidity is considered a significant modifiable risk factor that can influence long-term prognosis in cancer patients [110]. The present analysis, which revealed reduced perioperative morbidity and mortality observed with minimally invasive approaches, particularly RDP, supports the concept of improved long-term outcomes. On the other hand, taking into account that the long-term endpoints were not the focus of the present analysis, the oncologic implications of our findings should be interpreted with caution.

Although not in the scope of this study, cost-effectiveness warrants consideration. The cost disparity between robotic and laparoscopic abdominal surgery has persisted over time [111]. The main drivers of increased expenses are consumable supplies, longer operative times, and the substantial capital investment required for robotic systems. Nevertheless, some studies and meta-analyses have reported lower overall costs for RDP compared with LDP and ODP [66,112], a finding also supported by a prospective trial [43]. The overall economic balance, however, remains debated and is strongly influenced by local healthcare systems, institutional resources, and surgical volume.

The heterogeneity in pathologic entities included in our analysis represents an important limitation that warrants acknowledgment. Malignant pancreatic tumors, particularly pancreatic ductal adenocarcinoma, necessitate fundamentally different surgical approaches compared to benign pancreatic lesions [113,114,115]. Malignant lesions require extensive retroperitoneal dissection, radical lymphadenectomy, and often more complex vascular reconstructions to achieve oncological adequacy [116,117]. These procedures are associated with increased operative complexity, prolonged operative times, higher blood loss, and elevated complication rates compared to resections for benign conditions. In contrast, benign pancreatic lesions such as serous cystadenomas, mucinous cystic neoplasms, and pancreatic neuroendocrine tumors typically require less extensive dissection with parenchyma-preserving techniques being prioritized [118]. The differential surgical complexity between malignant and benign pathology directly impacts perioperative outcomes, with malignant cases demonstrating higher morbidity profiles. Our pooled analysis offers important insights into how different surgical methods compare in terms of effectiveness across the spectrum of pancreatic pathology. However, these findings should be interpreted recognizing the inherent heterogeneity in diseases, tumor biology, and required surgical complexity.

To address potential concerns regarding overlapping cohorts or duplicate populations, we implemented systematic measures during study selection and data extraction [119,120]. Studies originating from the same institutions or national databases were carefully examined for overlapping time periods and patient populations, with particular attention paid to large multi-institutional series that could share common patients. When overlapping populations were suspected, we prioritized studies with the most comprehensive datasets or longest follow-up periods and excluded those with clear evidence of duplicate reporting to prevent double-counting of patients and outcomes. The heterogeneity observed in our analysis may partly reflect the inclusion of studies from different healthcare systems and time periods, which actually reduces the likelihood of substantial population overlap while enhancing the generalizability of our findings. Additionally, our network meta-analysis methodology is inherently robust to minor population overlaps, as the statistical framework accounts for between-study heterogeneity that would be expected when some degree of patient duplication exists [121].

Most published robotic series originate from high-volume expert centers, where outcomes are likely influenced by surgical expertise, institutional experience, and advanced stages of the learning curve. This may limit the generalizability of the results to lower-volume centers or surgeons at the beginning of their robotic practice. Moreover, this study focused exclusively on short-term perioperative outcomes; this limits our ability to draw firm conclusions regarding oncologic equivalence among the three approaches. The predominance of retrospective observational studies, which are inherently subject to selection bias, unmeasured confounding, and variability in patient selection, surgical expertise, and institutional practices, represents a limitation. While we attempted to mitigate these concerns by applying standardized quality assessment tools (Newcastle–Ottawa Scale for observational studies and Cochrane RoB-2 for RCTs), the risk of bias included in the primary data is partially translated in the final output. Nevertheless, the inclusion of prospective studies and one randomized trial adds strength to the overall evidence. Heterogeneity was present in patient selection, surgical expertise, and perioperative protocols, which reflects real-world clinical practice. Although many comparisons were indirect, statistical consistency across the network and the application of established meta-analytical methods strengthen the credibility of the results.

## 6. Conclusions

This network meta-analysis demonstrates that minimally invasive distal pancreatectomy is associated with clear perioperative advantages over the open approach. Among minimally invasive techniques, the robotic platform showed potential benefits, including lower conversion rates and reduced short-term mortality. These findings support the safety and feasibility of RDP, while underscoring the overall value of minimally invasive approaches in distal pancreatectomy. However, interpretation should remain cautious, as most robotic data derive from high-volume expert centers, which may limit generalizability.

## Figures and Tables

**Table 1:** Risk of bias assessment using the Newcastle–Ottawa Scale and the Cochrane Risk of Bias 2.0 tool.

Study (Year)	Total NOS (0–9)
Rodriguez (2018) [25]	9
Abu Hilal (2012) [26]	9
Alfieri (2019) [27]	9
Aly (2010) [28]	7
Beker (2009) [29]	9
Benizri (2014) [30]	8
van Bodegraven (2024) [31]	9
Butturini (2011) [32]	8
Butturini (2015) [33]	8
Casadei (2010) [34]	9
Chang (2024) [35]	9
Chen (2022) [36]	9
Chen (2023) [37]	9
Daouadi (2013) [38]	8
De Pastena (2021) [39]	8
De Pastena (2024) [40]	9
Ding (2023) [41]	7
DiNorcia (2010) [42]	6
Duran (2014) [43]	7
Eom (2008) [44]	7
Finan (2009) [45]	7
Fox (2012) [46]	8
Goh (2016) [47]	7
Guerrero-Ortiz (2024) [48]	7
Hong (2020) [49]	8
Jarufe (2018) [50]	9
Jiang (2020) [51]	9
Kamarajah (2022) [52]	8
Kang (2010) [53]	8
Khaled (2015) [54]	9
Kim(2008) [55]	6
Kooby (2008) [56]	8
Lai (2015) [57]	9
Lai (2022) [58]	8
Lee (2015) [59]	9
Lee (2020) [60]	7
Lelpo (2017) [61]	7
Limongelli (2012) [62]	8
Liu (2017) [63]	9
Lof (2021) [64]	9
Lyman (2019) [65]	9
Magge (2018) [66]	9
Marino (2019) [67]	9
Mehta (2012) [68]	8
Morelli (2016) [69]	9
Najafi (2020) [70]	9
Nakamura (2009) [71]	7
Nakamura (2015) [72]	9
Nickel (2023) [73]	9
Qu (2018) [74]	9
Raoof (2018) [75]	9
Shin (2023) [76]	9
Souche (2018) [77]	7
Stauffer (2013) [78]	9
Velanovich (2006) [79]	8
Vicente (2019) [80]	7
Vijan (2010) [81]	9
Waters (2010) [82]	7
Wellner (2017) [83]	9
Weng (2021) [84]	9
Xourafas (2017) [85]	9
Yan (2015) [86]	7
Zhang (2017) [87]	7
Zhang (2022) [88]	9
Cho (2011) [89]	9
Chopra (2021) [90]	9
**Study (year)**	**Cochrane Risk of Bias 2.0 tool**
Björnsson (2020) [8]	4/5

**Table 2:** Baseline characteristics of the included studies.

Study	Country	Study Period	Study Type (RCT/Retrospective/Retrospective + Prospectively Held Database/Prospective Observational/Prospective Observational CU Propensity Match)	No. Included Patients	Comparaison (RDP vs. LDP vs. ODP)
Rodriguez_2018 [25]	France	2012–2015	Retrospective with prospectively maintained database	89	RDP vs. LDP vs. ODP
Abu Hilal_2012 [26]	UK	2005–2011	Retrospective from a prospectively held database	51	LDP vs. ODP
Alfieri_2019 [27]	Italy	2008–2016	Retrospective	181	LDP vs. RDP
Aly_2010 [28]	Japan	1998–2009	Retrospective	75	LDP vs. ODP
Beker_2009 [29]	USA	2003–2008	Prospective non-randomized	112	LDP vs. ODP
Benizri_2014 [30]	France	2004–2011	Retrospective with prospectively maintained database	34	LDP vs. RDP
Björnsson_2020 [8]	Sweden	2015–2019	RCT	58	LDP vs. ODP
van Bodegraven_2024 [31]	Pan-European	2019–2021	Retrospective with prospectively maintained database	1672	RDP vs. LDP
Butturini_2011 [32]	Italy	1999–2006	Retrospective non-randomized study	116	LDP vs. ODP
Butturini_2015 [33]	Italy	2011–2014	Prospective non-randomized	43	LDP vs. RDP
Casadei_2010 [34]	Italy	2000–2010	Retrospective case–control study	44	LDP vs. ODP
Chang_2024 [35]	USA	2010–2020	Retrospective propensity score matching	1537	LDP vs. RDP
Chen_2022 [36]	China	2013–2019	Retrospective case study	149	LDP vs. RDP
Chen_2023 [37]	Internation	2010–2019	Retrospective	542	LDP vs. RDP
Daouadi_2013 [38]	USA	2004–2011	Retrospective	124	LDP vs. RDP
De Pastena_2021 [39]	Italy	2011–2017	Retrospective propensity score matching	103	LDP vs. RDP
De Pastena_2024 [40]	Italy	2010–2020	Retrospective	564	LDP vs. RDP
Ding_2023 [41]	UK	2008–2023	Retrospective with prospectively maintained database	123	LDP vs. RDP
DiNorcia_2010 [42]	USA	1991–2009	Retrospective with prospectively maintained database	387	LDP vs. ODP
Duran_2014 [43]	Spain	2008–2013	Retrospective	47	LDP vs. ODP vs. RDP
Eom_2008 [44]	Korea	1995–2006	Retrospective case–control study	93	LDP vs. ODP
Finan_2009 [45]	USA	2002–2007	Retrospective	148	LDP vs. ODP
Fox_2012 [46]	Canada	2004–2010	Retrospective	118	LDP vs. ODP
Goh_2016 [47]	Singapore	2006–2015	Retrospective with prospectively maintained database	39	LDP vs. RDP
Guerrero-Ortiz_2024 [48]	Spain	2022	Prospective, multicenter national observational study	80	LDP vs. RDP
Hong_2020 [49]	Republic of Korea	2015–2017	Retrospective	228	LDP vs. RDP
Jarufe_2018 [50]	Chile	2001–2015	Retrospective	93	LDP vs. ODP
Jiang_2020 [51]	China	2011–2018	Retrospective	166	LDP vs. RDP
Kamarajah_2022 [52]	UK	2007–2018	Retrospective	125	LDP vs. RDP vs. ODP
Kang_2010 [53]	Korea	1999–2008	Retrospective	32	LDP vs. ODP
Khaled_2015 [54]	UK	2002–2011	Retrospective case-matched	44	LDP vs. ODP
Kim_2008 [55]	Republic of Korea	-	Retrospective	128	LDP vs. ODP
Kooby_2008 [56]	USA	2002–2006	Retrospective multicenter cohort from a prospectively held database	667	LDP vs. ODP
Lai_2015 [57]	China	1999–2015	Retrospective from a prospectively held database	35	LDP vs. RDP
Lai_2022 [58]	Taiwan	2011–2020	Retrospective using a prospectively maintained database	177	LDP vs. RDP
Lee_2015 [59]	USA	200–2013	Retrospective	805	LDP vs. RDP vs. ODP
Lee_2020 [60]	Singapore	2006–2019	Retrospective from a prospectively held database	102	LDP vs. RDP
Lelpo_2017 [61]	Spain	2011–2017	Retrospective	54	LDP vs. RDP
Limongelli_2012 [62]	Italy	2000–2010	Retrospective from a prospectively held database	45	LDP vs. ODP
Liu_2017 [63]	China	2011–2015	Retrospective propensity score-matched study	355	LDP vs. RDP
Lof_2021 [64]	Europe	2011–2019	Retrospective, propensity score matching	1551	LDP vs. RDP
Lyman_2019 [65]	USA	2008–2017	Retrospective from a prospectively held database	249	LDP vs. RDP
Magge_2018 [66]	USA	2010–2016	Retrospective from a prospectively held database	374	LDP vs. ODP vs. RDP
Marino_2019 [67]	Italy	2014–2017	Retrospective case-matched	70	LDP vs. RDP
Mehta_2012 [68]	France	1998–2009	Retrospective case–control study	60	LDP vs. ODP
Morelli_2016 [69]	Italy	2010–2014	Retrospective case-matched	30	LDP vs. RDP
Najafi_2020 [70]	Germany	2008–2018	Retrospective	56	LDP vs. RDP
Nakamura_2009 [71]	Japan	2000–2007	Retrospective	36	LDP vs. ODP
Nakamura_2015 [72]	Japan	2006–2013	Retrospective Propensity score matching	2010	LDP vs. ODP
Nickel_2023 [73]	Germany	2007–2020	Retrospective case-matched	512	LDP vs. RDP vs. ODP
Qu_2018 [74]	China	2011–2015	Retrospective propensity score matching	70	LDP vs. RDP
Raoof_2018 [75]	USA	2010–2013	Retrospective	704	LDP vs. RDP
Shin_2023 [76]	Korea	2015–2020	Retrospective propensity score-matched study	42	LDP vs. RDP
Souche_2018 [77]	France	2011–2016	Prospective non-randomized	38	LDP vs. RDP
Stauffer_2013 [78]	USA	2005–2011	Retrospective	172	LDP vs. ODP
Velanovich_2006 [79]	USA	1996–2005	Retrospective case-matched	30	LDP vs. ODP
Vicente_2019 [80]	Spain	2014–2018	Prospective non-randomized	59	LDP vs. RDP
Vijan_2010 [81]	USA	2004–2009	Retrospective case-matched	200	LDP vs. ODP
Waters_2010 [82]	USA	2008–2009	Retrospective from a prospectively held database	57	LDP vs. ODP vs. RDP
Wellner_2017 [83]	Germany	2013–2016	Retrospective propensity score-matched study	198	LDP vs. ODP
Weng_2021 [84]	China	2012–2019	Retrospective case–control study	679	RDP vs. ODP
Xourafas_2017 [85]	USA	2014	Retrospective	1815	RDP vs. LDP vs. ODP
Yan_2015 [86]	China	2010–2012	Retrospective	91	LDP vs. ODP
Zhang_2017 [87]	China	2010–2017	Retrospective	74	LDP vs. RDP
Zhang_2022 [88]	China	2020–2021	Retrospective	201	LDP vs. RDP
Cho_2011 [89]	USA	1999–2008	Retrospective using a prospectively maintained database	693	LDP vs. ODP
Chopra_2021 [90]	USA	2008–2019	Retrospective with prospectively maintained database	146	LDP vs. ODP vs. RDP

**Table 3:** Summary of findings.

Outcome		Number of Studies	Number of Patients	Robotic vs. Open		Laparoscopic vs. Open		Robotic vs. Laparoscopic		Ranks Based on SUCRA Values: I, II, III
				Frequentist NMA	Bayesian NMA	Frequentist NMA	Bayesian NMA	Frequentist NMA	Bayesian NMA	
Population characteristics	Age of patients (years) (Frequentist—MD, 95% CI; Bayesian—MD, 95% CrI)	59	17,542	−1.65 [−3.00; −0.30]	−1.67 (−3.11, −0.27)	−0.72 [−1.86; 0.41]	−0.74 (−1.95, 0.43)	−0.93 [−1.93; 0.07]	−0.93 (−1.97, 0.11)	Robotic Laparoscopic Open
	Sex of patients (male) (Frequentist—OR, 95% CI; Bayesian—OR, 95% CrI)	63	18,030	0.73 [0.64; 0.82]	-	0.76 [0.69; 0.84]	-	0.95 [0.87; 1.05]	-	Robotic Laparoscopic Open
	ASA status (grade I–II) (Frequentist—OR, 95% CI; Bayesian—OR, 95% CrI)	40	10,318	-	1.24 (0.87, 1.8)	-	1.29 (0.94, 1.78)	-	0.96 (0.74, 1.27)	Laparoscopic Robotic Open
	Previous cardiovascular diseases (Frequentist—OR, 95% CI; Bayesian—OR, 95% CrI)	12	3307	0.99 [0.62; 1.61]	0.84 (0.41, 1.45)	0.94 [0.69; 1.29]	0.94 (0.62, 1.37)	1.19 [0.72; 1.96]	0.9 (0.46, 1.49)	Laparoscopic Robotic Open
Intra-operative characteristics	Operative time (minutes) (Frequentist—MD, 95% CI; Bayesian—MD, 95% CrI)	61	16,230	25.93 [7.68; 44.18]	27.17 (4.27, 50.33)	7.63 [−7.59; 22.85]	7.84 (−11.24, 26.94)	18.30 [5.12; 31.49;]	19.32 (2.74, 36.27)	Open Laparoscopic Robotic
	Conversion to open (Frequentist—OR, 95% CI; Bayesian—OR, 95% CrI)	38	10,586	-	-	-	-	0.41 [0.34; 0.49]	0.30 [0.22; 0.40]	Robotic Laparoscopic
	Intraoperative blood loss (mL) (Frequentist—MD, 95% CI; Bayesian—MD, 95% CrI)	51	12,257	−279.45 [−318.28; −240.61]	−303.98 (−382.8, −229.26)	−248.99 [−283.41; −214.57]	−272.89 (−340.15, −209.01)	−30.45 [ −54.66; −6.25]	−31.17 (−81.92, 19.44)	Robotic Laparoscopic Open
	Intraoperative bleeding > 500 mL (Frequentist—OR, 95% CI; Bayesian—OR, 95% CrI)	3	1427	0.11 [0.01; 1.07]	-	0.32 [0.23; 0.46]	-	0.33 [0.03; 3.24]	-	Robotic Laparoscopic Open
	Number of patients receiving transfusions (Frequentist—OR, 95% CI; Bayesian—OR, 95% CrI)	30	9248	0.25 [0.19; 0.34]	-	0.30 [0.24; 0.37]	-	0.85 [0.66; 1.10]	-	Robotic Laparoscopic Open
	The quantity of blood transfusion (Frequentist—MD, 95% CI; Bayesian—MD, 95% CrI)	4	363	1.98 [−3.42; −0.54]	-	−1.86 [−3.12; −0.59]	-	−0.12 [−1.14; 0.89]	-	Robotic Laparoscopic Open
Postoperative characteristics	ICU stay (Frequentist—MD, 95% CI; Bayesian—MD, 95% CrI)	9	1272	−4.01 [−5.97; −2.05]	-	−2.27 [−3.71; −0.83]	-	−1.74 [−3.52; 0.04]	-	Robotic Laparoscopic Open
	Reintervention rate (Frequentist—OR, 95% CI; Bayesian—OR, 95% CrI)	37	11,568	-	0.45 (0.23, 0.84)	-	0.56 (0.32, 0.96)	-	0.81 (0.47, 1.3)	Robotic Laparoscopic Open
	Hospital stay (Frequentist—MD, 95% CI; Bayesian—MD, 95% CrI)	63	18,113	−7.63 [−8.65; −6.61]	−8.77 (−13.34, −4.22)	−6.47 [−7.33; −5.60]	−6.93 (−10.67, −3.23)	−1.16 [−1.88; −0.44]	−1.83 (−5.17, 1.55)	Robotic Laparoscopic Open
Morbidity and Mortality	Readmission rate (Frequentist—OR, 95% CI; Bayesian—OR, 95% CrI)	31	12,330	0.93 [0.67; 1.30]		0.80 [0.59; 1.09]		0.86 [0.71; 1.05]		Laparoscopic Robotic Open
	In-hospital mortality (Frequentist—OR, 95% CI; Bayesian—OR, 95% CrI)	9	1009	-	1.14 × 10^−7^ (5.812–24, 1.36 × 10^6^)	0.76 [0.21; 2.72]	0.42 [0.04; 2.57]	-	-	Robotic Laparoscopic Open
	30-day mortality (Frequentist—OR, 95% CI; Bayesian—OR, 95% CrI)	31	12,127	0.37 [0.16; 0.84]	-	0.68 [0.40; 1.17]	-	0.54 [0.27; 1.07]	-	Robotic Laparoscopic Open
	90-day major complications (Frequentist—OR, 95% CI; Bayesian—OR, 95% CrI)	3	1427	0.86 [0.44; 1.68]	0.87 (0.36, 2.42)	0.88 [0.51; 1.52]	0.9 (0.45, 2.3)	0.98 [0.56; 1.71]	0.97 (0.42, 1.99)	Robotic Laparoscopic Open

95% CI—95% confidence interval; 95% CrI—95% credible interval; MD—mean difference; NMA—network meta-analysis; OR—odds ratio; SUCRA—surface under the cumulative ranking curve.

**Table 4:** Treatment effects for all studies: Comparison of all treatment pairs. Outcome: 30-day mortality. Bayesian NMA.

	Laparoscopic	Open	Robotic
Laparoscopic	Laparoscopic	2.1 (1.05, 6.1)	0.34 (0.1, 0.83)
Open	0.48 (0.16, 0.95)	Open	0.16 (0.03, 0.46)
Robotic	2.97 (1.2, 9.78)	6.29 (2.18, 34.05)	Robotic

## Data Availability

The data presented in this study are available on request from the corresponding authors.

## Supplementary Materials

The following supporting information can be downloaded at: https://www.mdpi.com/article/10.3390/cancers17193243/s1, File S1: Aff of the Supplementary materials.
