# Supplementary material for: Perioperative Outcomes in Robotic, Laparoscopic, and Open Distal Pancreatectomy: A Network Meta-Analysis and Meta-Regression
Source: Cancers (Basel). 2025 Oct 6;17(19):3243. doi: 10.3390/cancers17193243 (PMC12523380; doi:10.3390/cancers17193243)
Supplement: Supplementary file 1 [file cancers-17-03243-s001.zip › cancers-3876473-supplementary.pdf]

Supplementary material: Perioperative outcomes in robotic, laparoscopic and open distal pancreatectomy: a network meta-analysis and meta-regression

Nasser Abdul Halim[1], Eran Sadot[1], Ionut Negoi[2]

[1] Rabin Medical Center, Beilinson Hospital, affiliated to the medical faculty of Tel Aviv University, Petah Tikva, Israel

[2] Carol Davila University of Medicine and Pharmacy Bucharest, Clinical Emergency Hospital of Bucharest, Romania

Corresponding authors:

Nasser Abdul Halim M.D, Beilinson Hospital, Petah Tikva, Israel.

Email: shac.x15@gmail.com, nasserah@clalit.org.il

Ionut Negoi M.D. Ph.D., Carol Davila University of Medicine and Pharmacy Bucharest, Clinical Emergency Hospital of Bucharest, No. 8 Floreasca Street, Sector 1, 014461, Bucharest, Romania. E-mail: ionut.negoi@umfcd.ro, ORCID ID: 0000-0002-6950-9599

Table of Contents

1. Age of the patients 18
2. Sex of patients 30
3. ASA status 35
4. Previous cardiovascular diseases 40
5. Operative time 50
6. Conversion to open 60
7. Intraoperative blood loss 68
8. Intraoperative bleeding more than 500 ml 80
9. Number of patients receiving transfusions 82
10. The quantity of blood tranfusion 86
11. ICU stay 89
12. Reintervention rate 93
13. Hospital stay 98
14. Readmission rate 108
15. In-hospital mortality 112
16. 30-day mortality 120
17. 90-day major complications 130

Supplementary Figure S1: Individual study results grouped by treatment comparison for the outcome Age. 18

Supplementary Figure S2: Network plot of all studies for outcome Age. The size of the nodes and thickness of edges represent the number of studies that examined a treatment and compared two given treatments respectively. 19

Supplementary Figure S3: Forest plot for outcome age. The size of the nodes and thickness of edges represent the number of studies that examined a treatment and compared two given treatments respectively 19

Supplementary Figure S4: Summary Forrest Plot for outcome Age. Ranking of the interventions based on the SUCRA value 21

Supplementary Figure S5: Bayesian random effect consistency model forrest plot for outcome Age. Between studies standard deviation 2.31, 95% credible interval 1.67 to 3.06. 22

Supplementary Figure S6: Litmus Rank-O-Gram: Outcomes Age. Higher SUCRA (Surface Under the Cumulative Ranking Curve) values and cumulative ranking curves nearer the top left indicate better performance 23

Supplementary Figure S7: Radial SUCRA plot: Outcomes Age. Higher SUCRA values indicate better treatments; size of nodes represent number of participants and thickness of lines indicate number of trials conducted 24

Supplementary Figure S8: Residual deviance from NMA model and UME inconsistency model for all studies. 25

Supplementary Figure S9: Per-arm residual deviance for all studies. This stem plot represents the posterior residual deviance per study arm 26

Supplementary Figure S10: Leverage plot for all studies. 27

Supplementary Figure S11: Metaregression for the outcome Age having as covariate the year of study publication. 28

Supplementary Figure S12: Forrest plot of metaregression for the outcome age having as covariate the year of the study publication. 29

Supplementary Figure S13: Metaregression for the outcome Age having as covariate the number of patients in the included studies. 29

Supplementary Figure S14: Individual study results grouped by treatment comparison for the outcome Sex. 31

Supplementary Figure S15: Network plot of all studies for outcome Sex. The size of the nodes and thickness of edges represent the number of studies that examined a treatment and compared two given treatments respectively. 32

Supplementary Figure S16: Forest plot for outcome Sex. The size of the nodes and thickness of edges represent the number of studies that examined a treatment and compared two given treatments respectively.. 33

Supplementary Figure S17: Summary Forrest Plot for outcome Sex. Ranking of the interventions based on the SUCRA value.. 34

Supplementary Figure S18: Individual study results grouped by treatment comparison for the outcome ASA I-II. 35

Supplementary Figure S19: Network plot of all studies for outcome ASA I-II. The size of the nodes and thickness of edges represent the number of studies that examined a treatment and compared two given treatments respectively. 36

Supplementary Figure S20: Bayesian random effect consistency model forrest plot for outcome ASA I-II. Between studies standard deviation 0.49, 95% credible interval 0.26 to 0.77. 36

Supplementary Figure S21: Litmus Rank-O-Gram: Outcomes ASA I-II. Higher SUCRA (Surface Under the Cumulative Ranking Curve) values and cumulative ranking curves nearer the top left indicate better performance. 37

Supplementary Figure S22: Radial SUCRA plot: Outcomes ASA I-II. Higher SUCRA values indicate better treatments; size of nodes represent number of participants and thickness of lines indicate number of trials conducted. 38

Supplementary Figure S23: Inconsistency test with nodesplitting model for all studies for outcome ASA I-II. 38

Supplementary Figure S24: Metaregression for the outcome ASA I-II having as covariate the year of study publication.. 39

Supplementary Figure S25: Individual study results grouped by treatment comparison for the outcome cardiovascular diseases. 40

Supplementary Figure S26: Network plot of all studies for outcome cardiovascular diseases. The size of the nodes and thickness of edges represent the number of studies that examined a treatment and compared two given treatments respectively. 41

Supplementary Figure S27: Forest plot for outcome cardiovascular diseases. The size of the nodes and thickness of edges represent the number of studies that examined a treatment and compared two given treatments respectively. Between-study standard deviation (log-odds scale): 0 , Number of studies: 12 , Number of treatments: 3.. 41

Supplementary Figure S28: Summary Forrest Plot for outcome cardiovascular diseases. Ranking of the interventions based on the SUCRA value. 43

Supplementary Figure S29: Bayesian random effect consistency model forrest plot for outcomecardiovascular diseases. Between-study standard deviation (log-odds scale): 0.27 . 95% credible interval: 0.01 , 0.77 44

Supplementary Figure S30: Litmus Rank-O-Gram: Outcomes cardiovascular diseases. Higher SUCRA (Surface Under the Cumulative Ranking Curve) values and cumulative ranking curves nearer the top left indicate better performance. 45

Supplementary Figure S31: Radial SUCRA plot: Outcomes cardiovascular diseases. Higher SUCRA values indicate better treatments; size of nodes represent number of participants and thickness of lines indicate number of trials conducted. 46

Supplementary Figure S32: Nodesplit model for the outcome operative duration. 46

Supplementary Figure S33: Residual deviance from NMA model and UME inconsistency model for all studies. 47

Supplementary Figure S34: Per-arm residual deviance for all studies. This stem plot represents the posterior residual deviance per study arm 47

Supplementary Figure S35: Leverage plot for all studies. 48

Supplementary Figure S36: Regression plot for the outcome cardiovascular diseases having as covariate the year of study publication. 49

Supplementary Figure S37: Individual study results grouped by treatment comparison for the outcome operative time. 50

Supplementary Figure S38: Network plot of all studies for outcome operative time. The size of the nodes and thickness of edges represent the number of studies that examined a treatment and compared two given treatments respectively 51

Supplementary Figure S39: Forest plot for outcome operative time. The size of the nodes and thickness of edges represent the number of studies that examined a treatment and compared two given treatments respectively. 51

Supplementary Figure S40: Summary Forrest Plot for outcome operative time. Ranking of the interventions based on the SUCRA value. 53

Supplementary Figure S41: Bayesian random effect consistency model forrest plot for outcome operative duration. Between studies standard deviation 51.49, 95% credible interval 41.93 to 63.28 54

Supplementary Figure S42: Litmus Rank-O-Gram: Outcomes operative duration. Higher SUCRA (Surface Under the Cumulative Ranking Curve) values and cumulative ranking curves nearer the top left indicate better performance. 55

Supplementary Figure S43: Radial SUCRA plot: Outcomes operative time. Higher SUCRA values indicate better treatments; size of nodes represent number of participants and thickness of lines indicate number of trials conducted. 56

Supplementary Figure S44: Inconsistency test with nodesplitting model for all studies for outcome opertative durationI. 56

Supplementary Figure S45: Residual deviance from NMA model and UME inconsistency model for all studies. 57

Supplementary Figure S46: Per-arm residual deviance for all studies. This stem plot represents the posterior residual deviance per study arm 57

Supplementary Figure S47: Leverage plot for all studies. 58

Supplementary Figure S48: Regression plot for the outcome operative duration having as covariate the year of study publication. 58

Supplementary Figure S49: Regression plot for the outcome operative duration having as covariate the number of patients in the included studies. 59

Supplementary Figure S50: Individual study results grouped by treatment comparison for the outcome conversions to open. 60

Supplementary Figure S51: Network plot of all studies for outcome conversions. The size of the nodes and thickness of edges represent the number of studies that examined a treatment and compared two given treatments respectively. 61

Supplementary Figure S52: Forest plot for outcome conversions. The size of the nodes and thickness of edges represent the number of studies that examined a treatment and compared two given treatments respectively. Between-study standard deviation (log-odds scale): 0.2 , Number of studies: 37 , Number of treatments: 2. All outcomes are versus the reference treatment: Laparoscopic 61

Supplementary Figure S53: Litmus Rank-O-Gram: Outcomes conversions. Higher SUCRA (Surface Under the Cumulative Ranking Curve) values and cumulative ranking curves nearer the top left indicate better performance. 62

Supplementary Figure S54: Forrest plot of metaregression for the outcome conversions having as covariate the number of patients in the included studies. Between-study standard deviation (log-odds scale): 0.39 . 95% credible interval: 0.02 , 0.81 63

Supplementary Figure S55: : Litmus Rank-O-Gram: Outcomes conversions. Higher SUCRA (Surface Under the Cumulative Ranking Curve) values and cumulative ranking curves nearer the top left indicate better performance. 64

Supplementary Figure S56: Radial SUCRA plot: Outcomes conversions. Higher SUCRA values indicate better treatments; size of nodes represent number of participants and thickness of lines indicate number of trials conducted. 65

Supplementary Figure S57: Convergence assessment plots for the outcomes conversion. 66

Supplementary Figure S58: Per-arm residual deviance for all studies. This stem plot represents the posterior residual deviance per study arm.. 67

Supplementary Figure S59: Leverage plot for all studies.. 67

Supplementary Figure S60: Individual study results grouped by treatment comparison for the outcome Intraoperative blood loss. 68

Supplementary Figure S61: Network plot of all studies for outcome Intraoperative bleeding. The size of the nodes and thickness of edges represent the number of studies that examined a treatment and compared two given treatments respectively. 69

Supplementary Figure S62: Forest plot for outcome Intraoperative bleeding. The size of the nodes and thickness of edges represent the number of studies that examined a treatment and compared two given treatments respectively. 69

Supplementary Figure S63: Summary Forrest Plot for outcome Intraoperative bleeding. Ranking of the interventions based on the SUCRA value. 71

Supplementary Figure S64: Bayesian random effect consistency model forrest plot for outcome Intraoperative bleeding. Between studies standard deviation 139.19, 95% credible interval 109.56 to 177.58. 71

Supplementary Figure S65: Litmus Rank-O-Gram: Outcomes Intraoperative bleeding. Higher SUCRA (Surface Under the Cumulative Ranking Curve) values and cumulative ranking curves nearer the top left indicate better performance. 72

Supplementary Figure S66: Radial SUCRA plot: Outcomes Intraoperative bleeding. Higher SUCRA values indicate better treatments; size of nodes represent number of participants and thickness of lines indicate number of trials conducted. 73

Supplementary Figure S67: The nodesplit model for the Bayesian NMA. 73

Supplementary Figure S68: Residual deviance from NMA model and UME inconsistency model for all studies.. 74

Supplementary Figure S69: Per-arm residual deviance for all studies. This stem plot represents the posterior residual deviance per study arm.. 74

Supplementary Figure S70: Leverage plot for all studies.. 75

Supplementary Figure S71: The summary characteristic plot having as co-variate year of publication across al treatment arms. 76

Supplementary Figure S72: Metaregression for the outcome Intraoperative bleeding having as covariate the year of study. publication. 77

Supplementary Figure S73: Forrest plot of metaregression for the outcome intraoperative bleeding having as covariate the year of the study publication. 78

Supplementary Figure S74: Metaregression for the outcome Intraoperative bleeding having as covariate the number of patients in the included studies. 78

Supplementary Figure S75: Forrest plot of metaregression for the outcome intraoperative bleeding having as covariate the number of patients in the included studies. 79

Supplementary Figure S76: Network plot of all studies for outcome intraoperative bleeding more 500 ml. The size of the nodes and thickness of edges represent the number of studies that examined a treatment and compared two given treatments respectively.................................... 80

Supplementary Figure S77. Forest plot for outcome intraoperative bleeding more 500 ml......... 80

Supplementary Figure S78: Individual study results grouped by treatment comparison for the outcome patients receiving blood transfusion... 82

Supplementary Figure S79: Network plot of all studies for outcome patients receiving blood transfusion. The size of the nodes and thickness of edges represent the number of studies that examined a treatment and compared two given treatments respectively.................................... 83

Supplementary Figure S80: Forest plot for outcome Number of patients receiving blood transfusions. 83

Supplementary Figure S81: Summary Forrest Plot for outcome number of patients having blood tranfusuions. Ranking of the interventions based on the SUCRA value. 84

Supplementary Figure S82: Individual study results grouped by treatment comparison for the outcome quantity blood transfused. 86

Supplementary Figure S83: Network plot of all studies for outcome quantity of blood transfused. The size of the nodes and thickness of edges represent the number of studies that examined a treatment and compared two given treatments respectively. 87

Supplementary Figure S84: Forest plot for outcome quantity of blood transfused. Between-study standard deviation: 0.53 , Number of studies: 3, Number of treatments: 3. All outcomes are versus the reference treatment: Open. 87

Supplementary Figure S85: Individual study results grouped by treatment comparison for the outcome ICU stay. 89

Supplementary Figure S86: Network plot of all studies for outcome ICU stay. The size of the nodes and thickness of edges represent the number of studies that examined a treatment and compared two given treatments respectively. ... 90

Supplementary Figure S87: Forest plot for outcome ICU stay. The size of the nodes and thickness of edges represent the number of studies that examined a treatment and compared two given treatments respectively. ... 90

Supplementary Figure S88: Summary Forrest Plot for outcome ICU stay. Ranking of the interventions based on the SUCRA value. ... 92

Supplementary Figure S89: Network plot of all studies for outcome reintervention rate. The size of the nodes and thickness of edges represent the number of studies that examined a treatment and compared two given treatments respectively. ... 93

Supplementary Figure S90: Bayesian random effect consistency model forrest plot for outcome reintervention rate. ... 93

Supplementary Figure S91: Litmus Rank-O-Gram: Outcomes reintervention rate. Higher SUCRA (Surface Under the Cumulative Ranking Curve) values and cumulative ranking curves nearer the top left indicate better performance. ... 94

Supplementary Figure S92: Radial SUCRA plot: Outcomes reintervention rate. Higher SUCRA values indicate better treatments; size of nodes represent number of participants and thickness of lines indicate number of trials conducted. ... 95

Supplementary Figure S93: Nodesplit model for the outcome reintervention rate. ... 95

Supplementary Figure S94: Residual deviance from NMA model and UME inconsistency model for all studies. ... 96

Supplementary Figure S95: Per-arm residual deviance for all studies. This stem plot represents the posterior residual deviance per study arm. ... 96

Supplementary Figure S96: Leverage plot for all studies. ... 97

Supplementary Figure S97: Individual study results grouped by treatment comparison for the outcome hospital stay. ... 98

Supplementary Figure S98: Network plot of all studies for outcome hospital stay. The size of the nodes and thickness of edges represent the number of studies that examined a treatment and compared two given treatments respectively. ... 99

Supplementary Figure S99: Forest plot for outcome hospital stay. Between-study standard deviation: 2.07 , Number of studies: 63 , Number of treatments: 3. All outcomes are versus the reference treatment: Open. ... 99

Supplementary Figure S100: Summary Forrest Plot for outcome hospital stay. Ranking of the interventions based on the SUCRA value. 101

Supplementary Figure S101: Bayesian random effect consistency model forrest plot for outcome hospital stay. Between-study standard deviation: 10.86 . 95% credible interval: 9.14 , 12.96 . 101

Supplementary Figure S102: Litmus Rank-O-Gram: Outcomes hospital stay. Higher SUCRA (Surface Under the Cumulative Ranking Curve) values and cumulative ranking curves nearer the top left indicate better performance. 102

Supplementary Figure S103: Radial SUCRA plot: Outcomes hospital stay. Higher SUCRA values indicate better treatments; size of nodes represent number of participants and thickness of lines indicate number of trials conducted. 103

Supplementary Figure S104: Nodesplit model for the outcome hospital stay. 104

Supplementary Figure S105: Residual deviance from NMA model and UME inconsistency model for all studies. 104

Supplementary Figure S106: Per-arm residual deviance for all studies. This stem plot represents the posterior residual deviance per study arm. 105

Supplementary Figure S107: Leverage plot for all studies. 105

Supplementary Figure S108: Regression plot for the outcome hospital stay having as covariate the year of study publication. 106

Supplementary Figure S109: Regression plot for the outcome hospital stay having as covariate the number of patients in the inlcuded studies. 107

Supplementary Figure S110: Individual study results grouped by treatment comparison for the outcome readmission rate. 108

Supplementary Figure S111: Network plot of all studies for outcome readmission rate. The size of the nodes and thickness of edges represent the number of studies that examined a treatment and compared two given treatments respectively. 109

Supplementary Figure S112: Forest plot for outcome readmission rate. The size of the nodes and thickness of edges represent the number of studies that examined a treatment and compared two given treatments respectively. Between-study standard deviation (log-odds scale): 0.22 , Number of studies: 29 , Number of treatments: 3. 109

Supplementary Figure S113: Summary Forrest Plot for outcome readmission rate. Ranking of the interventions based on the SUCRA value. 111

Supplementary Figure S114: Individual study results grouped by treatment comparison for the outcome in-hospital mortality. 112

Supplementary Figure S115: Network plot of all studies for outcome in-hospital mortality. The size of the nodes and thickness of edges represent the number of studies that examined a treatment and compared two given treatments respectively. 113

Supplementary Figure S116: Forest plot for outcome in-hospital mortality. The size of the nodes and thickness of edges represent the number of studies that examined a treatment and compared two given treatments respectively.. 113

Supplementary Figure S117: Bayesian random effect consistency model forrest plot for outcome in-hospital mortality... 114

Supplementary Figure S118: Litmus Rank-O-Gram: Outcomes in-hospital mortality. Higher SUCRA (Surface Under the Cumulative Ranking Curve) values and cumulative ranking curves nearer the top left indicate better performance. 114

Supplementary Figure S119: Radial SUCRA plot: Outcomes in-hospital mortality. Higher SUCRA values indicate better treatments; size of nodes represent number of participants and thickness of lines indicate number of trials conducted. 115

Supplementary Figure S120: Residual deviance from NMA model and UME inconsistency model for all studies.. 116

Supplementary Figure S121: Per-arm residual deviance for all studies. This stem plot represents the posterior residual deviance per study arm.. 116

Supplementary Figure S122: Leverage plot for all studies.. 117

Supplementary Figure S123: Regression plot for the outcome in-hospital mortality having as covariate the year of study publication. 118

Supplementary Figure S124: Regression plot for the outcome in-hospital mortality having as covariate the number of patients in included studies. 119

Supplementary Figure S125: Individual study results grouped by treatment comparison for the outcome 30-day mortality. 120

Supplementary Figure S126: Network plot of all studies for outcome 30-day mortality. The size of the nodes and thickness of edges represent the number of studies that examined a treatment and compared two given treatments respectively. 121

Supplementary Figure S127: Forest plot for outcome 30-day mortality. With red – prediction intervals.. 121

Supplementary Figure S128: P-scores Heatmap for the outcome 30-day mortality. 122

Supplementary Figure S129: Forest plot for outcome 30-day mortality. Between-study standard deviation (log-odds scale): 0 , Number of studies: 20 , Number of treatments: 3. All outcomes are versus the reference treatment: Open... 122

Supplementary Figure S130: Summary Forrest Plot for outcome 30-day mortality. Ranking of the interventions based on the SUCRA value. 123

Supplementary Figure S131: Nodesplit model for the outcome 30-day mortality. 124

Supplementary Figure S132: evaluation of inconsistency in CINEMA. 124

Supplementary Figure S133: Residual deviance from NMA model and UME inconsistency model for all studies. 124

Supplementary Figure S134: Per-arm residual deviance for all studies. This stem plot represents the posterior residual deviance per study arm 125

Supplementary Figure S135: Leverage plot for all studies. 125

Supplementary Figure S136: Regression plot for the outcome 30-day mortality having as covariate the year of study publication. 126

Supplementary Figure S137: Forrest plot of metaregression for the outcome 30-day mortality having as covariate the year of the study publication. 127

Supplementary Figure S138: Regression plot for the outcome 30-day mortality having as covariate the number of patients in the included studies. 128

Supplementary Figure S139: Forrest plot of metaregression for the outcome 30-day mortality having as covariate the number of patiemts in theincluded studies. 129

Supplementary Figure S140: Publication bias evaluation for the outcomes 30-day mortality... 129

Supplementary Figure S141: Individual study results grouped by treatment comparison for the outcome 90-day major complications. 130

Supplementary Figure S142: Network plot of all studies for outcome 90-day major complications. The size of the nodes and thickness of edges represent the number of studies that examined a treatment and compared two given treatments respectively 131

Supplementary Figure S143: Forest plot for outcome 90-day major complications. Between-study standard deviation (log-odds scale): 0.25 , Number of studies: 3 , Number of treatments: 3. All outcomes are versus the reference treatment: Open 131

Supplementary Figure S144: Summary Forrest Plot for outcome 90-day major complications. Ranking of the interventions based on the SUCRA value. 133

Supplementary Figure S145: Bayesian random effect consistency model forrest plot for outcome 90-day major complications. Between-study standard deviation (log-odds scale): 0.37 . 95% credible interval: 0.02 , 0.78 . 133

Supplementary Figure S146: Litmus Rank-O-Gram: Outcomes 90-day major complications. Higher SUCRA (Surface Under the Cumulative Ranking Curve) values and cumulative ranking curves nearer the top left indicate better performance. 134

Supplementary Figure S147: Radial SUCRA plot: Outcomes 90-day major complications. Higher SUCRA values indicate better treatments; size of nodes represent number of participants and thickness of lines indicate number of trials conducted. 135

Supplementary Figure S148: Nodesplit model for the outcome 90-day major complications... 135

Supplementary Figure S149: Residual deviance from NMA model and UME inconsistency model for all studies. 136

Supplementary Figure S150: Per-arm residual deviance for all studies. This stem plot represents the posterior residual deviance per study arm 136

Supplementary Figure S151: Leverage plot for all studies. 137

Supplementary Figure S152: Regression plot for the outcome 90-day major complications having as covariate the year of study publication. 138

Supplementary Table S1: Comparison of all treatment pairs related to outcome age. Treatments are ranked from best to worst along the leading diagonal. Above the leading diagonal are estimates from pairwise meta-analyses, below the leading diagonal are estimates from network meta-analyses. Relative treatment effects in ranked order for all studies.................................... 20

Supplementary Table S2: Assessment of inconsistency for all studies related to outcome age... 20

Supplementary Table S3: Treatment effects for all studies: comparison of all treatment pairs. Outcomes Age. Bayesian NMA.. 22

Supplementary Table S4: Comparison of all treatment pairs related to outcome Sex. Treatments are ranked from best to worst along the leading diagonal. Above the leading diagonal are estimates from pairwise meta-analyses, below the leading diagonal are estimates from network meta-analyses. Relative treatment effects in ranked order for all studies.................................... 33

Supplementary Table S5: Assessment of inconsistency for all studies related to outcome Sex. . 33

Supplementary Table S6: Treatment effects for all studies: comparison of all treatment pairs. Outcomes ASA I-II class. Bayesian NMA. 37

Supplementary Table S7: Comparison of all treatment pairs related to outcome cardiovascular diseases. Treatments are ranked from best to worst along the leading diagonal. Above the leading diagonal are estimates from pairwise meta-analyses, below the leading diagonal are estimates from network meta-analyses. Relative treatment effects in ranked order for all studies............. 42

Supplementary Table S8: Assessment of inconsistency for all studies related to outcome cardiovascular diseases. 42

Supplementary Table S9: Treatment effects for all studies: comparison of all treatment pairs. Outcomes Age. Bayesian NMA.. 44

Supplementary Table S10: Comparison of all treatment pairs related to outcome operative time. Treatments are ranked from best to worst along the leading diagonal. Above the leading diagonal are estimates from pairwise meta-analyses, below the leading diagonal are estimates from network meta-analyses. Relative treatment effects in ranked order for all studies 52

Supplementary Table S11: Assessment of inconsistency for all studies related to outcome operative time.. 52

Supplementary Table S12: Treatment effects for all studies: comparison of all treatment pairs. Outcomes operative duration. Bayesian NMA. 54

Supplementary Table S13: Comparison of all treatment pairs related to outcome conversions. Treatments are ranked from best to worst along the leading diagonal. Above the leading diagonal are estimates from pairwise meta-analyses, below the leading diagonal are estimates from network meta-analyses. Relative treatment effects in ranked order for all studies 61

Supplementary Table S14: Treatment effects for all studies: comparison of all treatment pairs. Outcomes conversions. Bayesian NMA. 62

Supplementary Table S15: Comparison of all treatment pairs related to outcome intraoperative bleeding. Treatments are ranked from best to worst along the leading diagonal. Above the leading diagonal are estimates from pairwise meta-analyses, below the leading diagonal are estimates from network meta-analyses. Relative treatment effects in ranked order for all studies 70

Supplementary Table S16: Assessment of inconsistency for all studies related to outcome Intraoperative bleeding. 70

Supplementary Table S17: Treatment effects for all studies: comparison of all treatment pairs. Outcomes Intraoperative bleeding. Bayesian NMA. 72

Supplementary Table S18: Comparison of all treatment pairs related to outcome intraoperative bleeding more 500 ml. Treatments are ranked from best to worst along the leading diagonal. Above the leading diagonal are estimates from pairwise meta-analyses, below the leading diagonal are estimates from network meta-analyses. Relative treatment effects in ranked order for all studies 81

Supplementary Table S19: Assessment of inconsistency for all studies related to outcome intraoperative bleeding more 500 ml. 81

Supplementary Table S20: Comparison of all treatment pairs related to outcome number of patients receiving transfusions. Treatments are ranked from best to worst along the leading diagonal. Above the leading diagonal are estimates from pairwise meta-analyses, below the leading diagonal are estimates from network meta-analyses. Relative treatment effects in ranked order for all studies 83

Supplementary Table S21: Assessment of inconsistency for all studies related to outcome number of patients with blood transfusions. 84

Supplementary Table S22: Comparison of all treatment pairs related to outcome quantity of blood transfused. Treatments are ranked from best to worst along the leading diagonal. Above the leading diagonal are estimates from pairwise meta-analyses, below the leading diagonal are estimates from network meta-analyses. Relative treatment effects in ranked order for all studies. 87

Supplementary Table S23: Assessment of inconsistency for all studies related to outcome quantity of blood transfused.. 88

Supplementary Table S24: Comparison of all treatment pairs related to outcome ICU stay. Treatments are ranked from best to worst along the leading diagonal. Above the leading diagonal are estimates from pairwise meta-analyses, below the leading diagonal are estimates from network meta-analyses. Relative treatment effects in ranked order for all studies 91

Supplementary Table S25: Assessment of inconsistency for all studies related to outcome ICU stay. 91

Supplementary Table S26: Treatment effects for all studies: comparison of all treatment pairs. Outcomes reinterention rate. Bayesian NMA. 94

Supplementary Table S27: Comparison of all treatment pairs related to outcome hospital stay. Treatments are ranked from best to worst along the leading diagonal. Above the leading diagonal are estimates from pairwise meta-analyses, below the leading diagonal are estimates from network meta-analyses. Relative treatment effects in ranked order for all studies. 100

Supplementary Table S28: Assessment of inconsistency for all studies related to outcome hospital stay. 100

Supplementary Table S29: Treatment effects for all studies: comparison of all treatment pairs. Outcomes hospital stay. Bayesian NMA. 102

Supplementary Table S30: Comparison of all treatment pairs related to outcome readmission rate. Treatments are ranked from best to worst along the leading diagonal. Above the leading diagonal are estimates from pairwise meta-analyses, below the leading diagonal are estimates from network meta-analyses. Relative treatment effects in ranked order for all studies. 110

Supplementary Table S31: Assessment of inconsistency for all studies related to outcome readmission rate. 110

Supplementary Table S32: Comparison of all treatment pairs related to outcome 30-day mortality. Treatments are ranked from best to worst along the leading diagonal. Above the leading diagonal are estimates from pairwise meta-analyses, below the leading diagonal are estimates from network meta-analyses. Relative treatment effects in ranked order for all studies 122

Supplementary Table S33: CINEMA league table. 122

Supplementary Table S34: Assessment of inconsistency for all studies related to outcome 30-day mortality. 123

Supplementary Table S35: Comparison of all treatment pairs related to outcome 90-day major complication. Treatments are ranked from best to worst along the leading diagonal. Above the leading diagonal are estimates from pairwise meta-analyses, below the leading diagonal are estimates from network meta-analyses. Relative treatment effects in ranked order for all studies 132

Supplementary Table S36: Assessment of inconsistency for all studies related to outcome 90-day major complications. 132

Supplementary Table S37: Treatment effects for all studies: comparison of all treatment pairs. Outcomes 90-day major complications. Bayesian NMA. 134

1. Age of the patients

Frequentist NMA

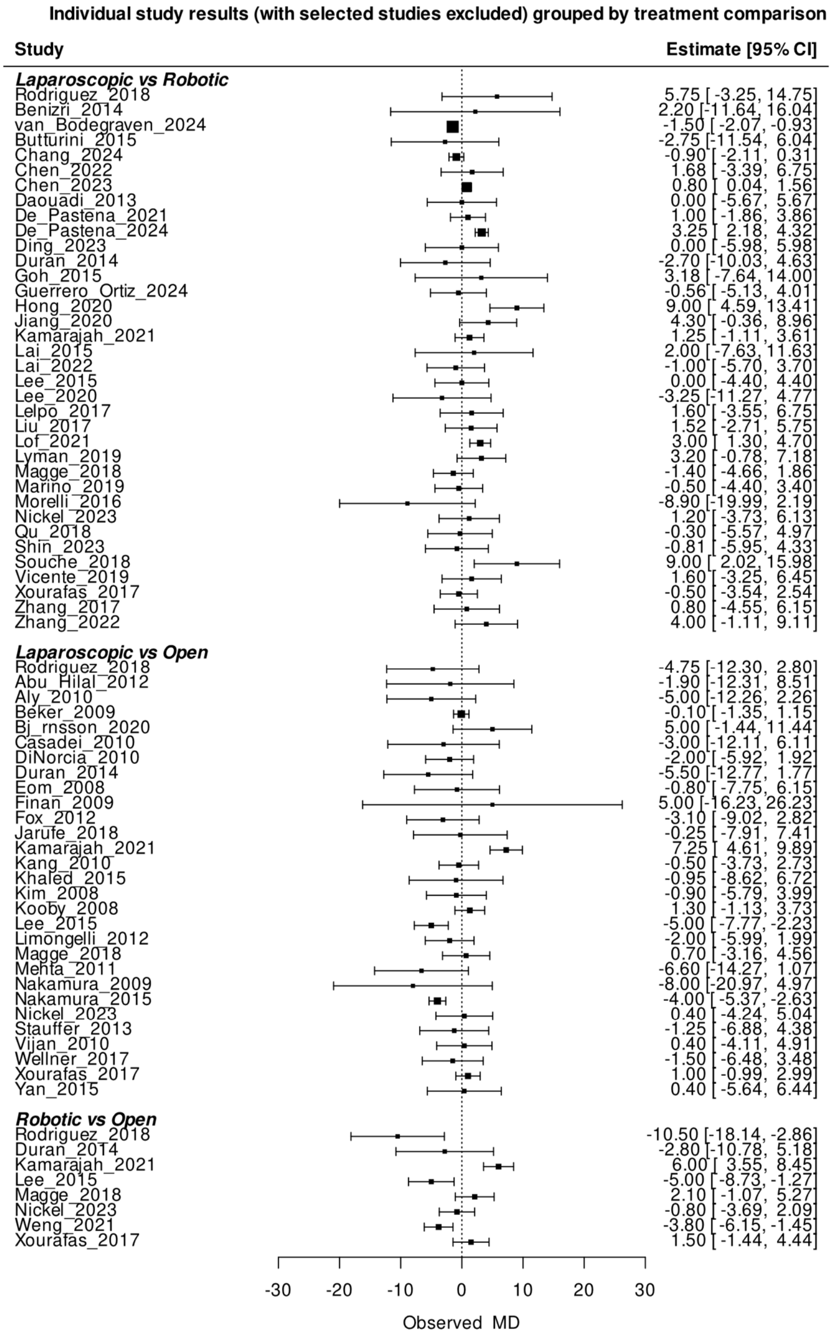

Supplementary Figure S1: Individual study results grouped by treatment comparison for the outcome Age.

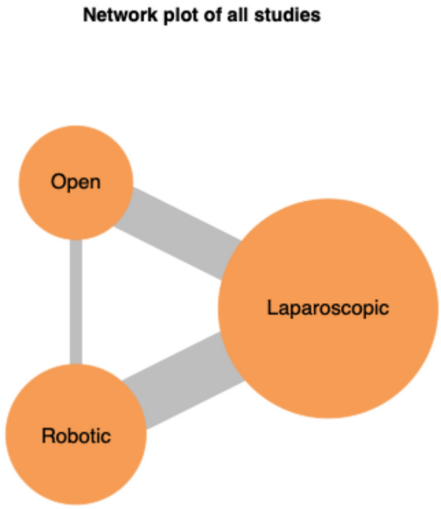

Supplementary Figure S2: Network plot of all studies for outcome Age. The size of the nodes and thickness of edges represent the number of studies that examined a treatment and compared two given treatments respectively.

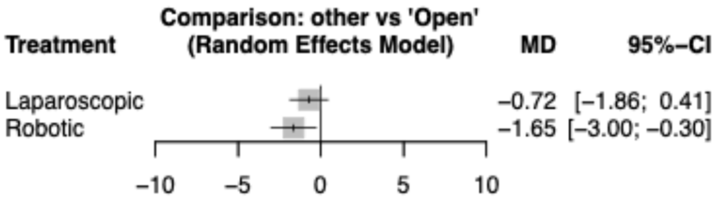

Supplementary Figure S3: Forest plot for outcome age. The size of the nodes and thickness of edges represent the number of studies that examined a treatment and compared two given treatments respectively.

Supplementary Table S1: Comparison of all treatment pairs related to outcome age. Treatments are ranked from best to worst along the leading diagonal. Above the leading diagonal are estimates from pairwise meta-analyses, below the leading diagonal are estimates from network meta-analyses. Relative treatment effects in ranked order for all studies

	Robotic	Laparoscopic	Open
**Robotic**	Robotic	-1.05 [-2.08; -0.01]	-0.56 [-2.56; 1.44]
**Laparoscopic**	-0.93 [-1.93; 0.07]	Laparoscopic	-0.81 [-2.02; 0.40]
**Open**	-1.65 [-3.00; -0.30]	-0.72 [-1.86; 0.41]	Open

Supplementary Table S2: Assessment of inconsistency for all studies related to outcome age.

	Comparison	No.Studies	NMA	Direct	Indirect	Difference	Diff_95CI_lower	Diff_95CI_upper	pValue
1	Laparoscopic:Open	29	-0.723973410787378	-0.812458112785672	-0.0612322414621663	-0.751225871323505	-4.27796626325485	2.77551452060783	0.676320699561054
2	Laparoscopic:Robotic	36	0.929452011571375	1.04788795803302	-0.557945876299199	1.60583383433222	-2.2052431588584	5.41691082752285	0.408889403280084
3	Robotic:Open	8	-1.65342542235875	-0.558195556917817	-2.57282517064541	2.01462961372759	-0.695797411577971	4.72505663903316	0.145166360271871

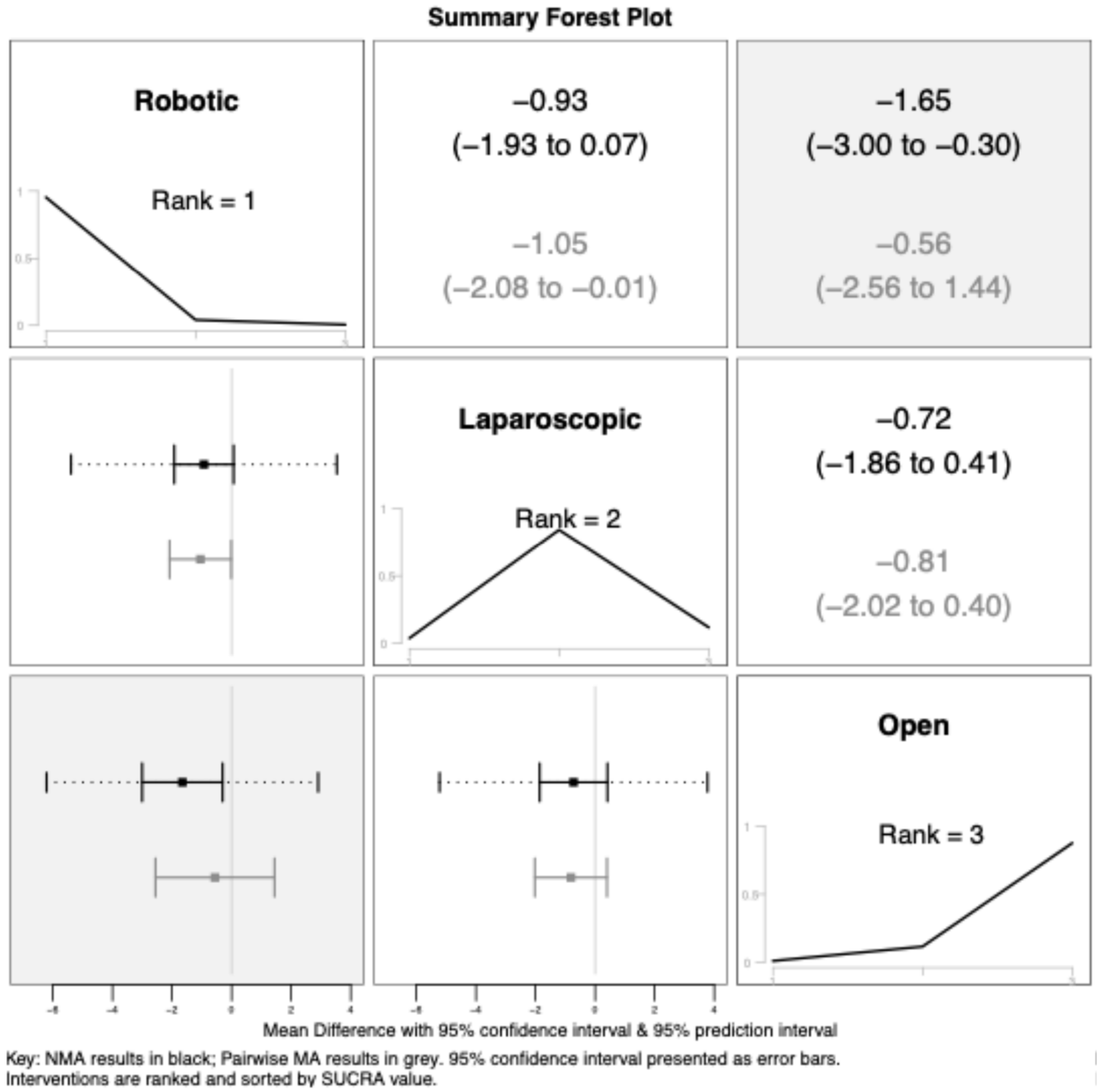

Supplementary Figure S4: Summary Forrest Plot for outcome Age. Ranking of the interventions based on the SUCRA value.

Bayesian NMA

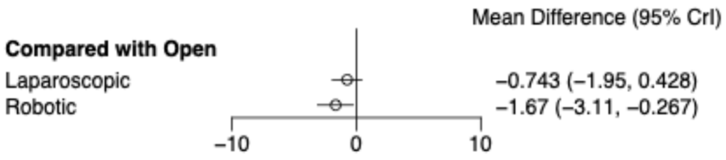

Supplementary Figure S5: Bayesian random effect consistency model forrest plot for outcome Age. Between studies standard deviation 2.31, 95% credible interval 1.67 to 3.06.

Supplementary Table S3: Treatment effects for all studies: comparison of all treatment pairs. Outcomes Age. Bayesian NMA.

	Laparoscopic	Open	Robotic
**Laparoscopic**	Laparoscopic	0.74 (-0.43, 1.95)	-0.93 (-1.97, 0.11)
**Open**	-0.74 (-1.95, 0.43)	Open	-1.67 (-3.11, -0.27)
**Robotic**	0.93 (-0.11, 1.97)	1.67 (0.27, 3.11)	Robotic

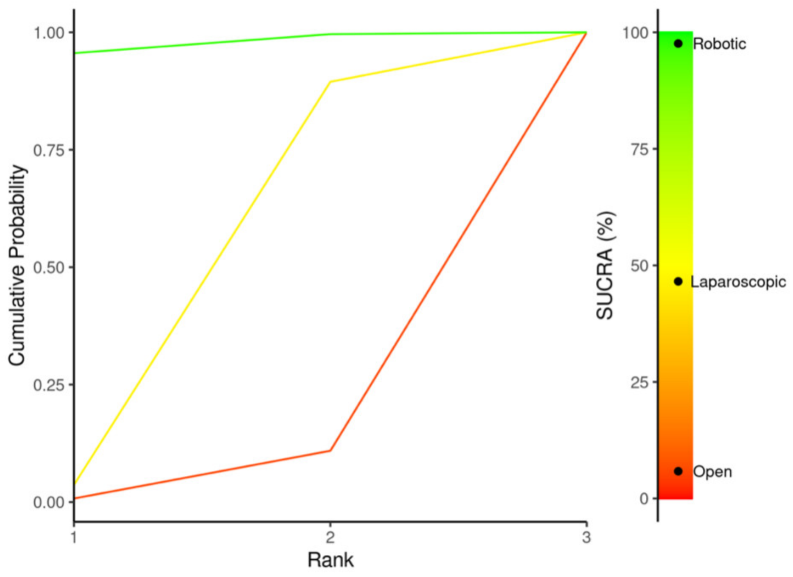

Supplementary Figure S6: Litmus Rank-O-Gram: Outcomes Age. Higher SUCRA (Surface Under the Cumulative Ranking Curve) values and cumulative ranking curves nearer the top left indicate better performance.

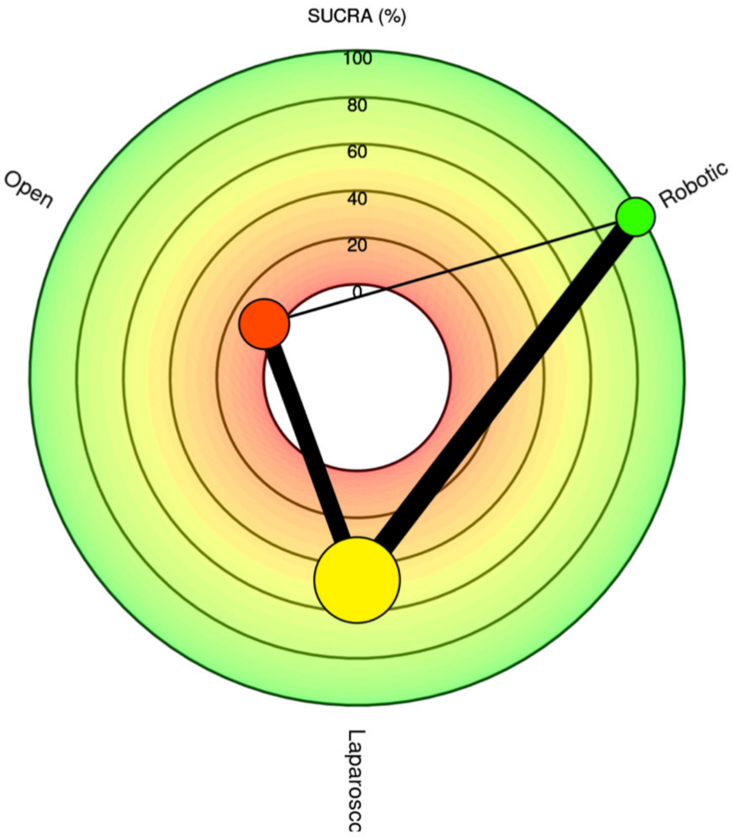

Supplementary Figure S7: Radial SUCRA plot: Outcomes Age. Higher SUCRA values indicate better treatments; size of nodes represent number of participants and thickness of lines indicate number of trials conducted.

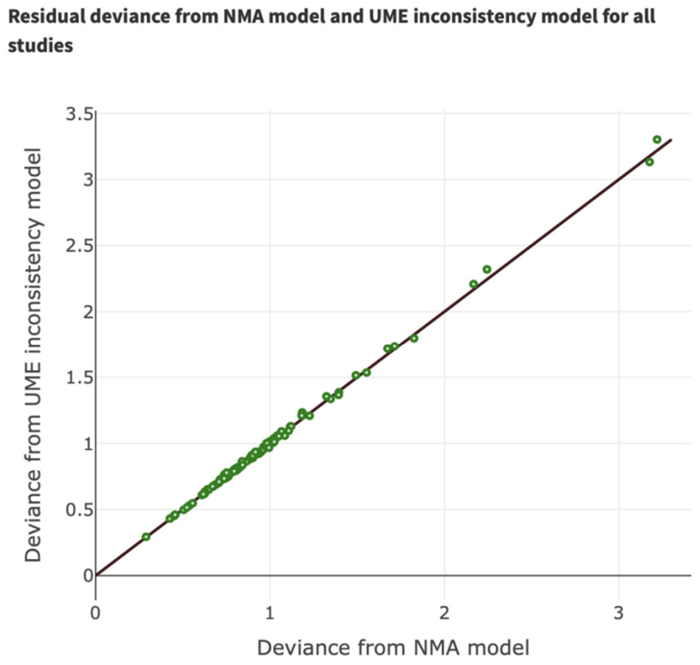

Supplementary Figure S8: Residual deviance from NMA model and UME inconsistency model for all studies.

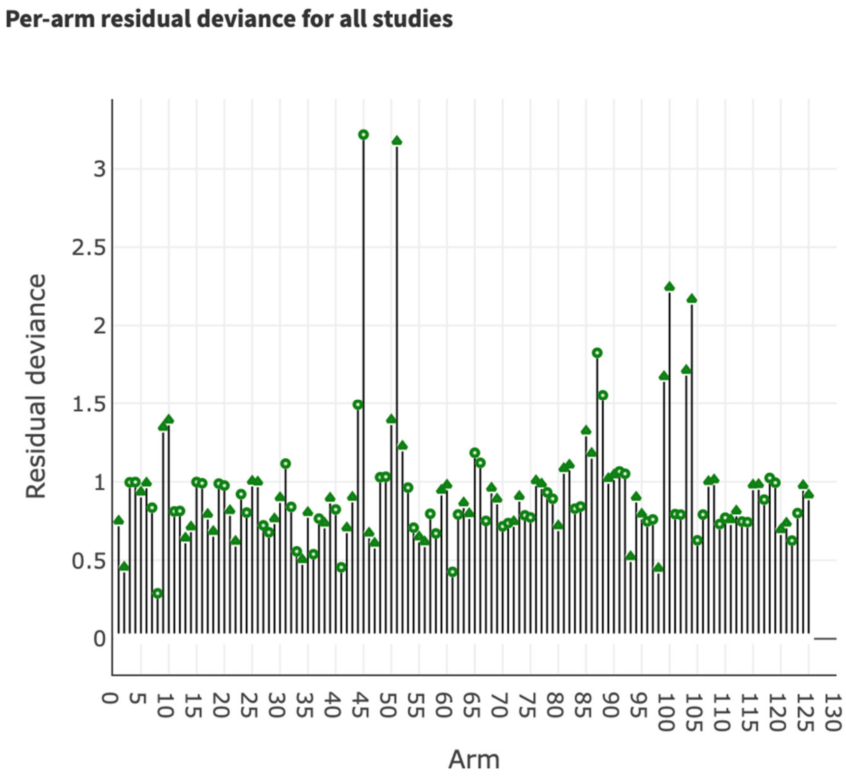

Supplementary Figure S9: Per-arm residual deviance for all studies. This stem plot represents the posterior residual deviance per study arm.

Leverage plot for all studies

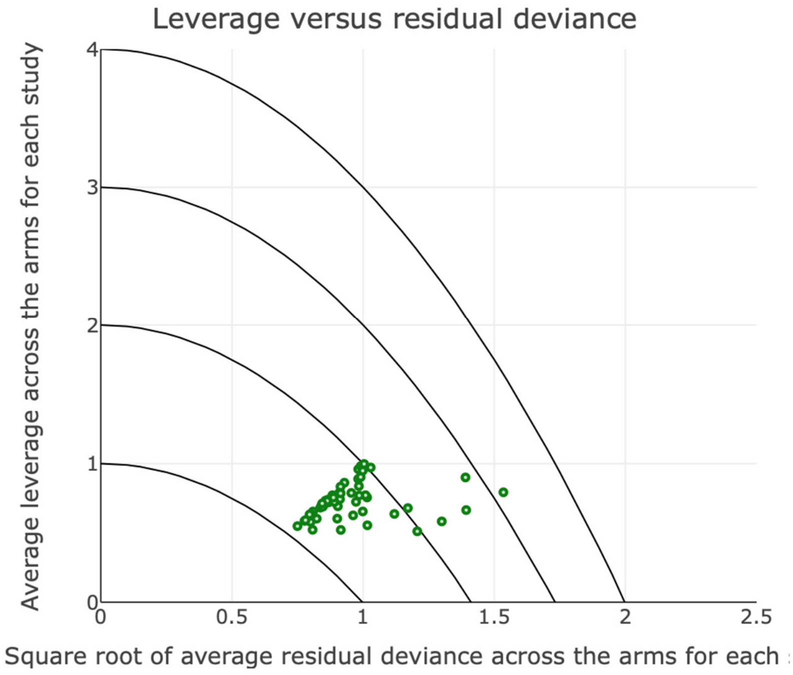

Supplementary Figure S10: Leverage plot for all studies.

Meta-regression

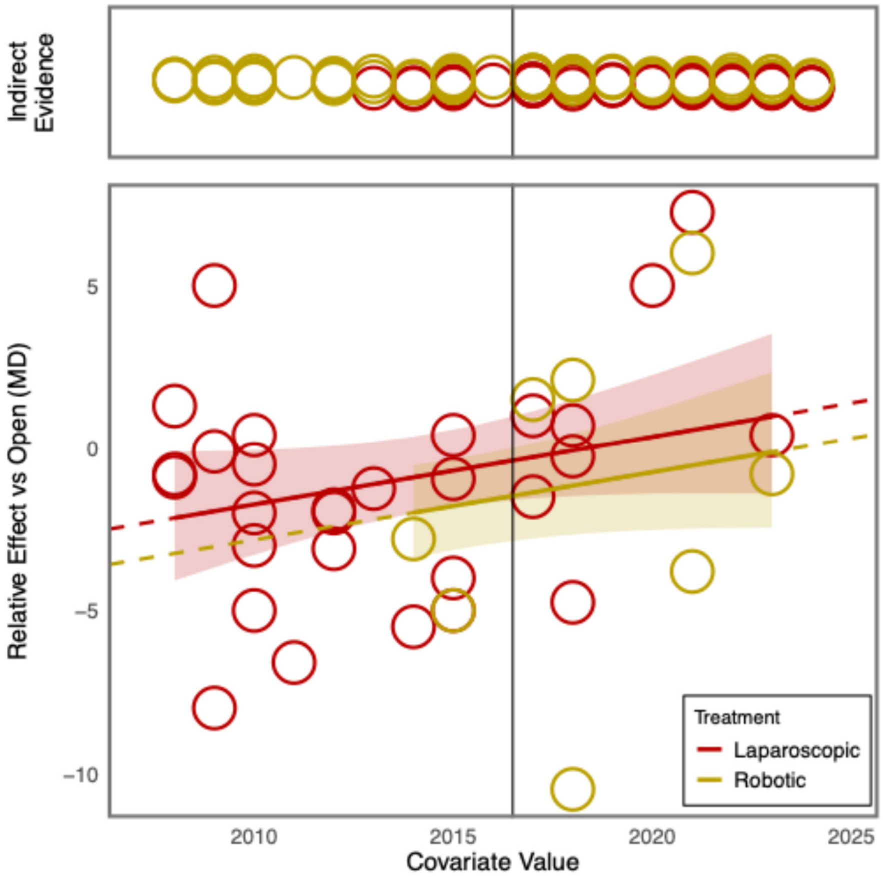

Supplementary Figure S11: Metaregression for the outcome Age having as covariate the year of study publication.

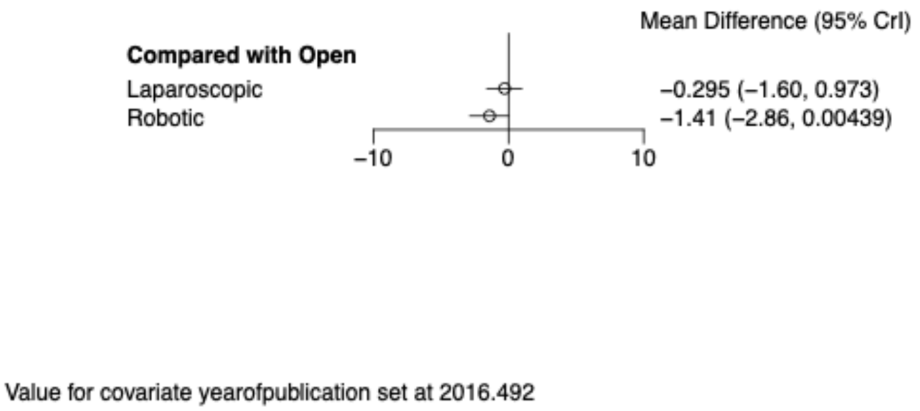

Supplementary Figure S12: Forrest plot of metaregression for the outcome age having as covariate the year of the study publication.

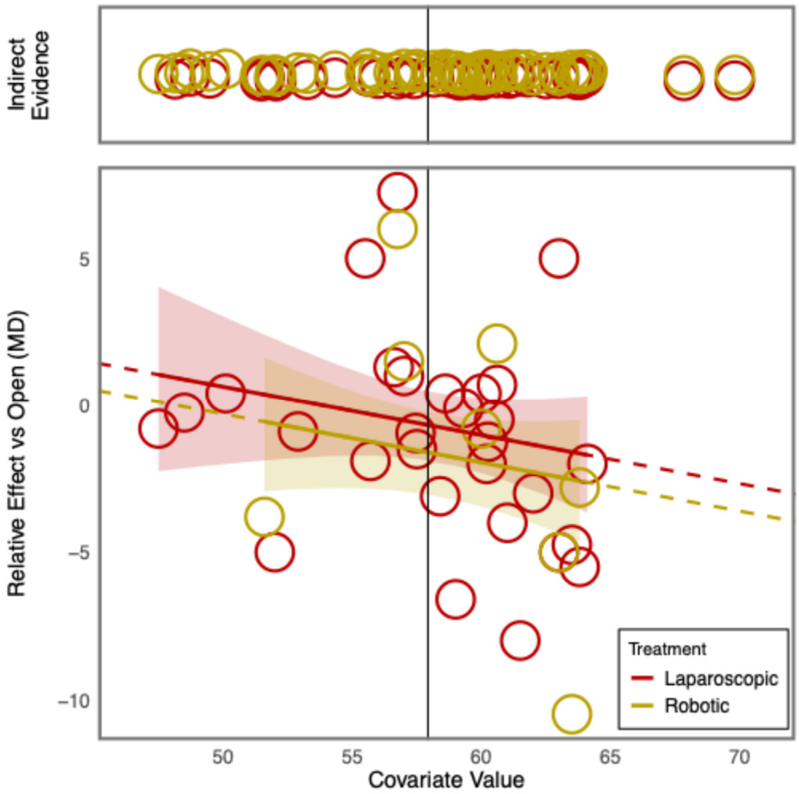

Supplementary Figure S13: Metaregression for the outcome Age having as covariate the number of patients in the included studies.

2. Sex of patients

A total of 63 studies, including 18,030 patients, reported sex distribution (Supplementary Figure S14). Of these, 55 were two-arm and 8 were multi-arm studies. The network included 79 pairwise comparisons. Nine trials compared ODP vs RDP, 30 compared ODP vs LDP, and 36 compared LDP vs RDP (Supplementary Figure S15).

In the frequentist NMA, both RDP (pooled Odds Ratio, OR [OR] = 0.73, 95% CI 0.64–0.82) and LDP (OR = 0.76, 95% CI 0.69–0.84) had a significantly lower proportion of male patients than the ODP (Supplementary Figure S16). No significant difference was observed between RDP and LDP (OR = 0.93; 95% CI: 0.85–1.03) (Supplementary Figure S17, supplementary Table 4). No evidence of inconsistency was observed between the direct and indirect estimates (Supplementary Table S5).

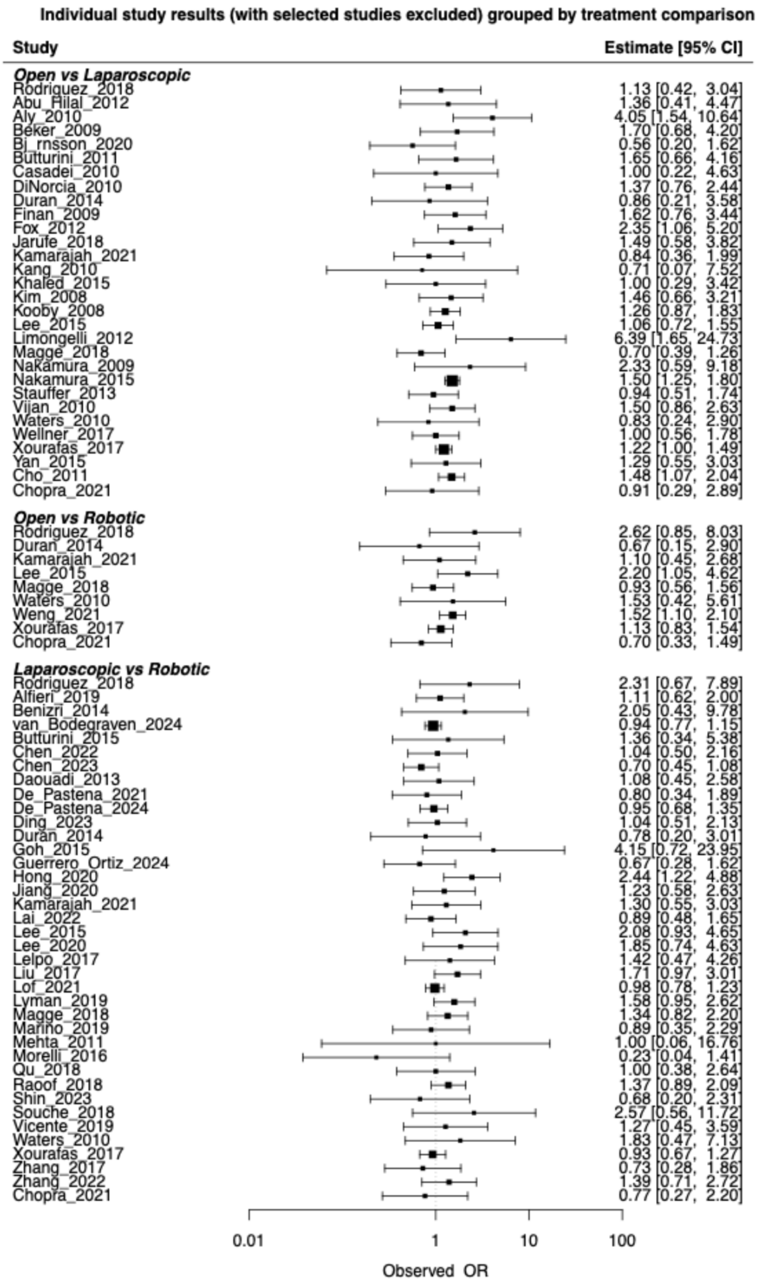

Supplementary Figure S14: Individual study results grouped by treatment comparison for the outcome Sex.

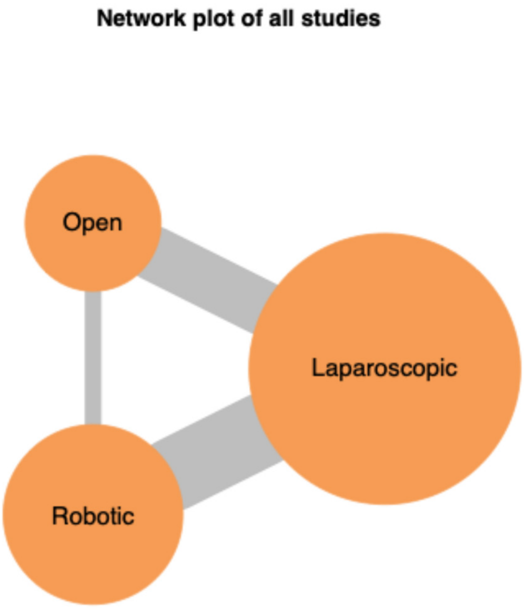

Supplementary Figure S15: Network plot of all studies for outcome Sex. The size of the nodes and thickness of edges represent the number of studies that examined a treatment and compared two given treatments respectively.

Frequentist NMA

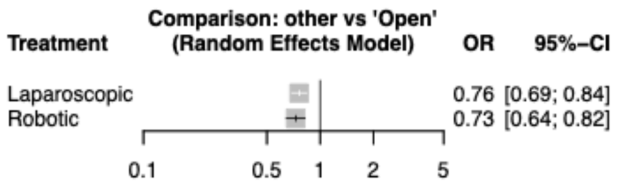

Supplementary Figure S16: Forest plot for outcome Sex. The size of the nodes and thickness of edges represent the number of studies that examined a treatment and compared two given treatments respectively.

Supplementary Table S4: Comparison of all treatment pairs related to outcome Sex. Treatments are ranked from best to worst along the leading diagonal. Above the leading diagonal are estimates from pairwise meta-analyses, below the leading diagonal are estimates from network meta-analyses. Relative treatment effects in ranked order for all studies.

	Robotic	Laparoscopic	Open
**Robotic**	Robotic	0.93 [0.85; 1.03]	0.80 [0.66; 0.97]
**Laparoscopic**	0.95 [0.87; 1.05]	Laparoscopic	0.76 [0.69; 0.84]
**Open**	0.73 [0.64; 0.82]	0.76 [0.69; 0.84]	Open

Supplementary Table S5: Assessment of inconsistency for all studies related to outcome Sex.

	Comparison	No.Studies	NMA	Direct	Indirect	Difference	Diff_95CI_lower	Diff_95CI_upper	pValue
1	Laparoscopic:Open	30	-0.271830774746157	-0.271361599594625	-0.275494240048995	0.00413264045437062	-0.300358622614517	0.308623903523259	0.978777833257442
2	Laparoscopic:Robotic	38	0.0464732854921395	0.0702441678965956	-0.202156546233755	0.272400714130351	-0.0596669069425284	0.60446833520323	0.107880790993562
3	Robotic:Open	9	-0.318304060238297	-0.221004495020797	-0.385434711687796	0.164430216666999	-0.0819791720529211	0.410839605386918	0.19090934530982

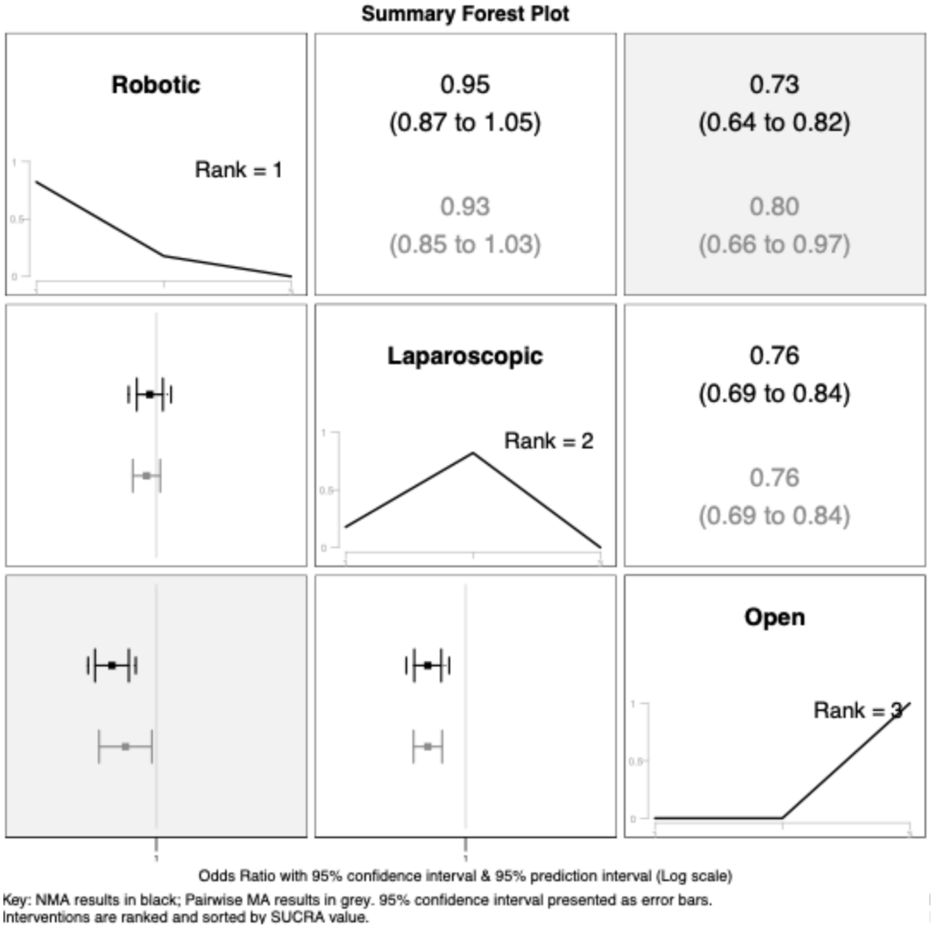

Supplementary Figure S17: Summary Forrest Plot for outcome Sex. Ranking of the interventions based on the SUCRA value.

3. ASA status

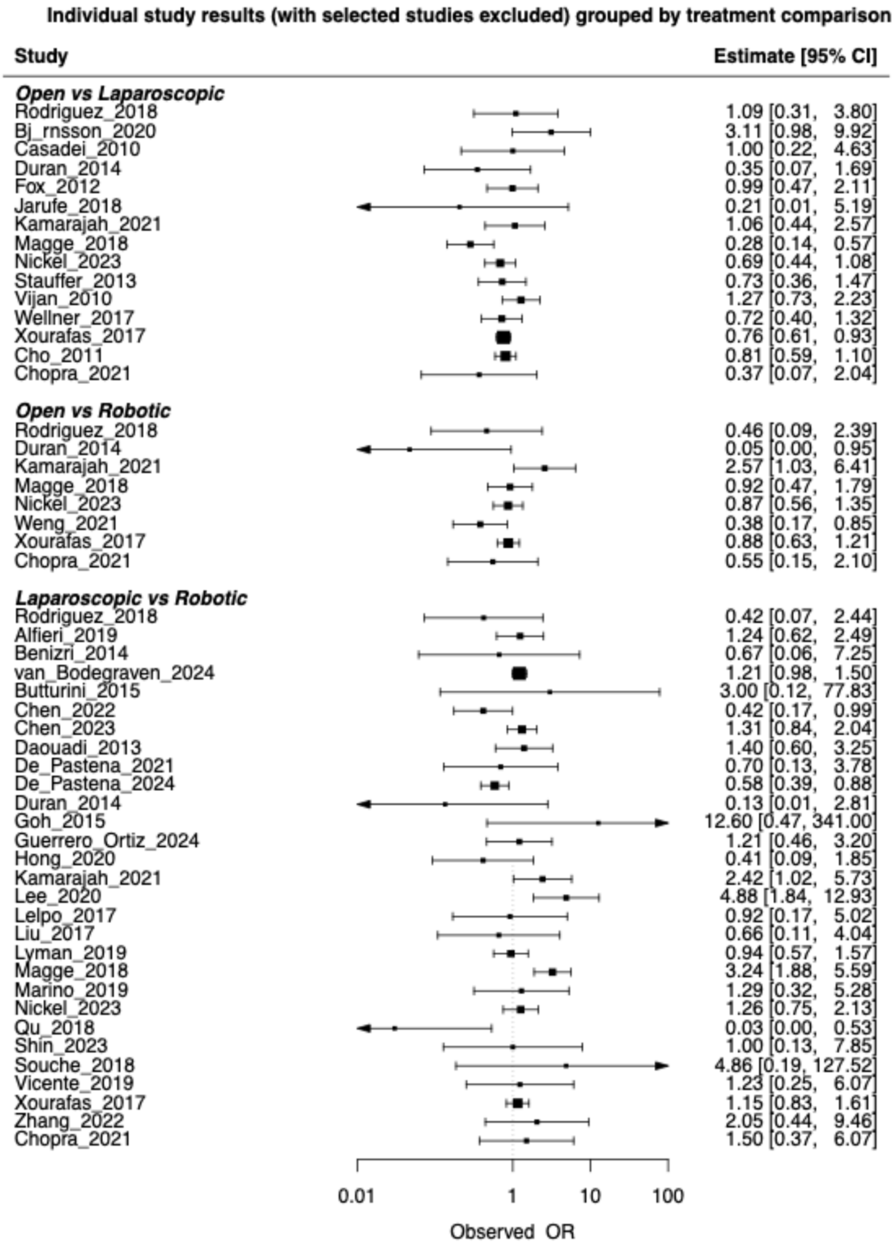

Supplementary Figure S18: Individual study results grouped by treatment comparison for the outcome ASA I-II.

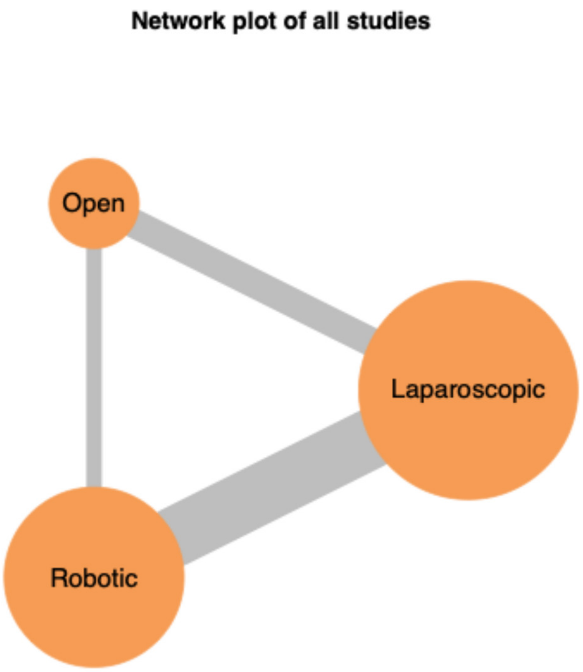

Supplementary Figure S19: Network plot of all studies for outcome ASA I-II. The size of the nodes and thickness of edges represent the number of studies that examined a treatment and compared two given treatments respectively.

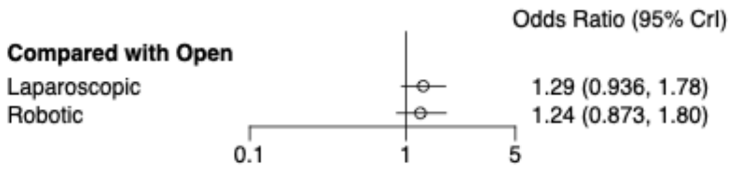

Supplementary Figure S20: Bayesian random effect consistency model forrest plot for outcome ASA I-II. Between studies standard deviation 0.49, 95% credible interval 0.26 to 0.77.

Supplementary Table S6: Treatment effects for all studies: comparison of all treatment pairs. Outcomes ASA I-II class. Bayesian NMA.

	Laparoscopic	Open	Robotic
**Laparoscopic**	Laparoscopic	0.78 (0.56, 1.07)	0.96 (0.74, 1.27)
**Open**	1.29 (0.94, 1.78)	Open	1.24 (0.87, 1.8)
**Robotic**	1.04 (0.79, 1.35)	0.81 (0.56, 1.15)	Robotic

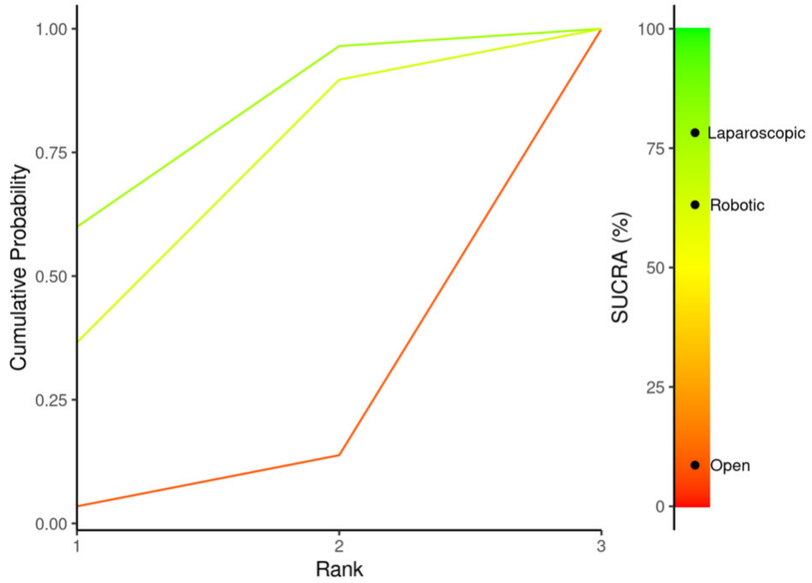

Supplementary Figure S21: Litmus Rank-O-Gram: Outcomes ASA I-II. Higher SUCRA (Surface Under the Cumulative Ranking Curve) values and cumulative ranking curves nearer the top left indicate better performance.

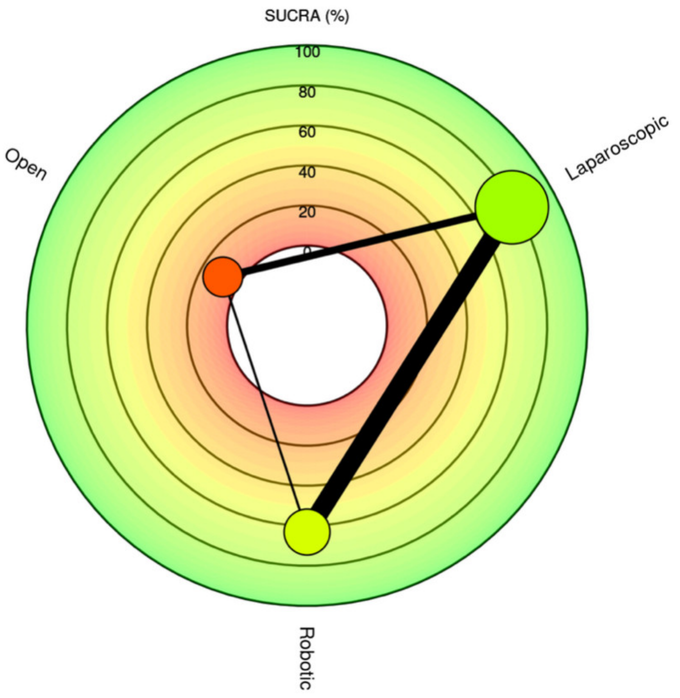

Supplementary Figure S22: Radial SUCRA plot: Outcomes ASA I-II. Higher SUCRA values indicate better treatments; size of nodes represent number of participants and thickness of lines indicate number of trials conducted.

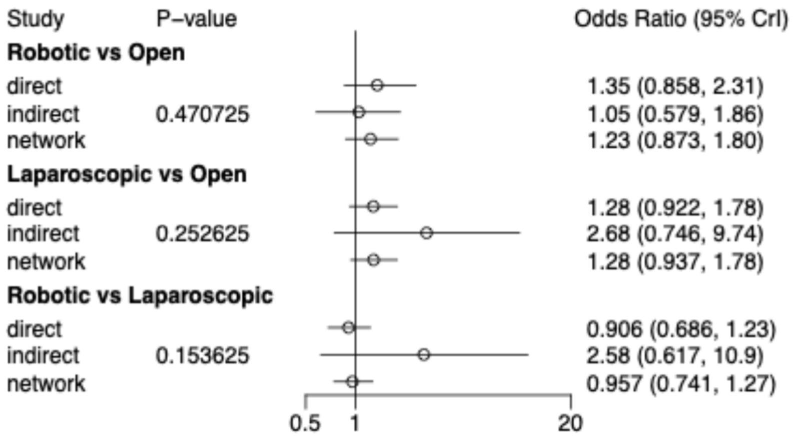

Supplementary Figure S23: Inconsistency test with nodesplitting model for all studies for outcome ASA I-II.

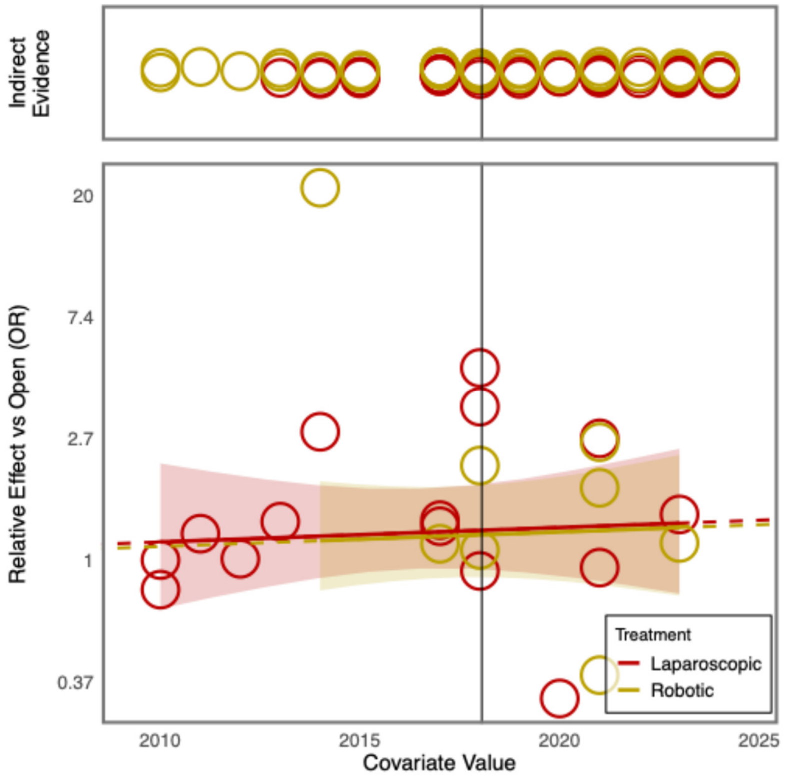

Supplementary Figure S24: Metaregression for the outcome ASA I-II having as covariate the year of study publication.

4. Previous cardiovascular diseases

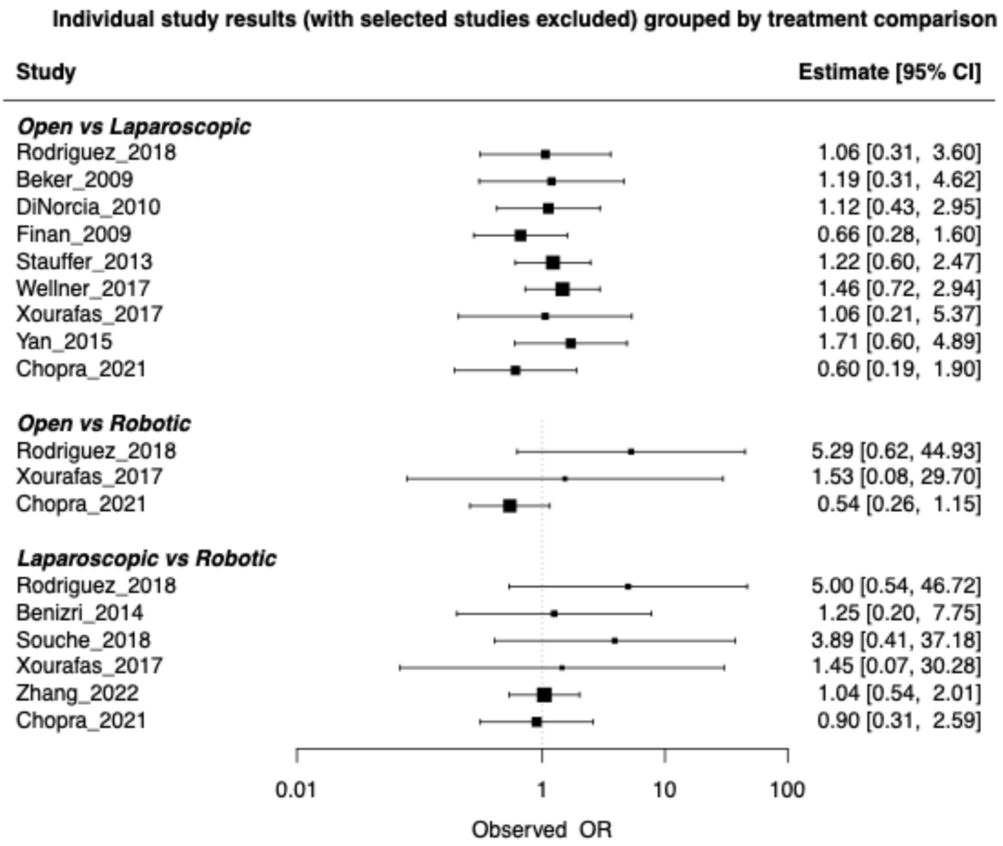

Supplementary Figure S25: Individual study results grouped by treatment comparison for the outcome cardiovascular diseases.

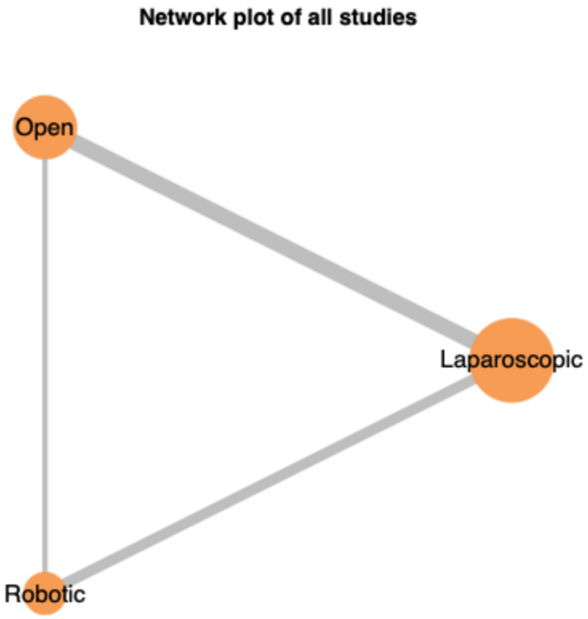

Supplementary Figure S26: Network plot of all studies for outcome cardiovascular diseases. The size of the nodes and thickness of edges represent the number of studies that examined a treatment and compared two given treatments respectively.

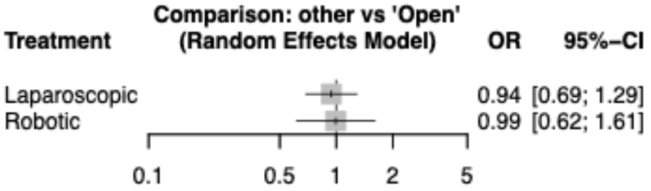

Supplementary Figure S27: Forest plot for outcome cardiovascular diseases. The size of the nodes and thickness of edges represent the number of studies that examined a treatment and compared two given treatments respectively. Between-study standard deviation (log-odds scale): 0 , Number of studies: 12 , Number of treatments: 3

Supplementary Table S7: Comparison of all treatment pairs related to outcome cardiovascular diseases. Treatments are ranked from best to worst along the leading diagonal. Above the leading diagonal are estimates from pairwise meta-analyses, below the leading diagonal are estimates from network meta-analyses. Relative treatment effects in ranked order for all studies.

	**Laparoscopic**	**Robotic**	**Open**
**Laparoscopic**	Laparoscopic	1.19 [0.72; 1.96]	0.90 [0.65; 1.24]
**Robotic**	0.95 [0.61; 1.48]	Robotic	1.38 [0.69; 2.73]
**Open**	0.94 [0.69; 1.29]	0.99 [0.62; 1.61]	Open

Supplementary Table S8: Assessment of inconsistency for all studies related to outcome cardiovascular diseases.

	**Comparison**	**No.Studies**	**NMA**	**Direct**	**Indirect**	**Difference**	**Diff_95CI_lower**	**Diff_95CI_upper**	**pValue**
**1**	Laparoscopic:Open	9	-0.0589911754875715	-0.10448583790025	0.611094786196309	-0.71558062409656	-1.99066465408722	0.559503405894102	0.271359521448009
**2**	Laparoscopic:Robotic	6	-0.053808157500433	0.171951107094265	-0.90428458829858	1.07623569539284	-0.0157623030011989	2.16823369378689	0.0533998621161568
**3**	Robotic:Open	3	-0.00518301798713842	0.318458973627791	-0.311010458364345	0.629469431992135	-0.329478964763673	1.58841782874794	0.198250323793178

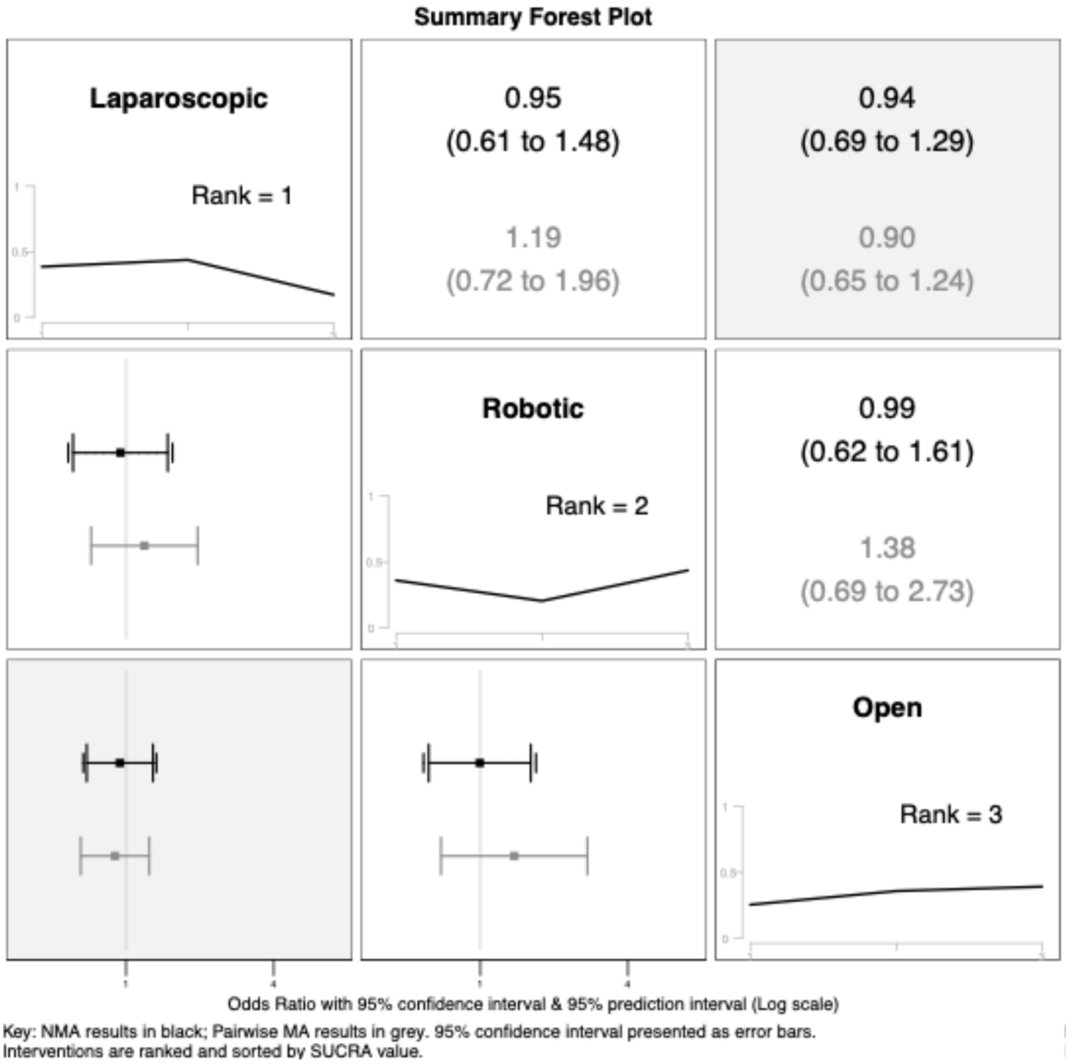

Supplementary Figure S28: Summary Forrest Plot for outcome cardiovascular diseases. Ranking of the interventions based on the SUCRA value.

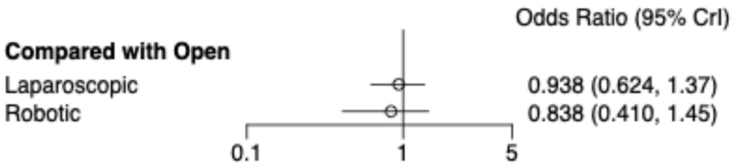

Supplementary Figure S29: Bayesian random effect consistency model forrest plot for outcomecardiovascular diseases. Between-study standard deviation (log-odds scale): 0.27 . 95% credible interval: 0.01 , 0.77 .

Supplementary Table S9: Treatment effects for all studies: comparison of all treatment pairs. Outcomes Age. Bayesian NMA.

	**Laparoscopic**	**Open**	**Robotic**
**Laparoscopic**	Laparoscopic	1.07 (0.73, 1.6)	0.9 (0.46, 1.49)
**Open**	0.94 (0.62, 1.37)	Open	0.84 (0.41, 1.45)
**Robotic**	1.11 (0.67, 2.16)	1.19 (0.69, 2.44)	Robotic

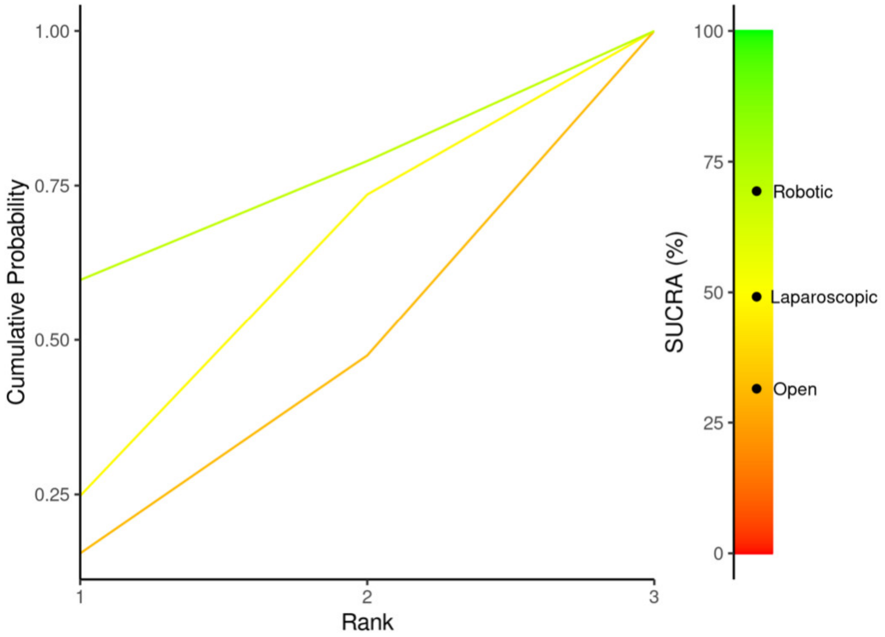

Supplementary Figure S30: Litmus Rank-O-Gram: Outcomes cardiovascular diseases. Higher SUCRA (Surface Under the Cumulative Ranking Curve) values and cumulative ranking curves nearer the top left indicate better performance.

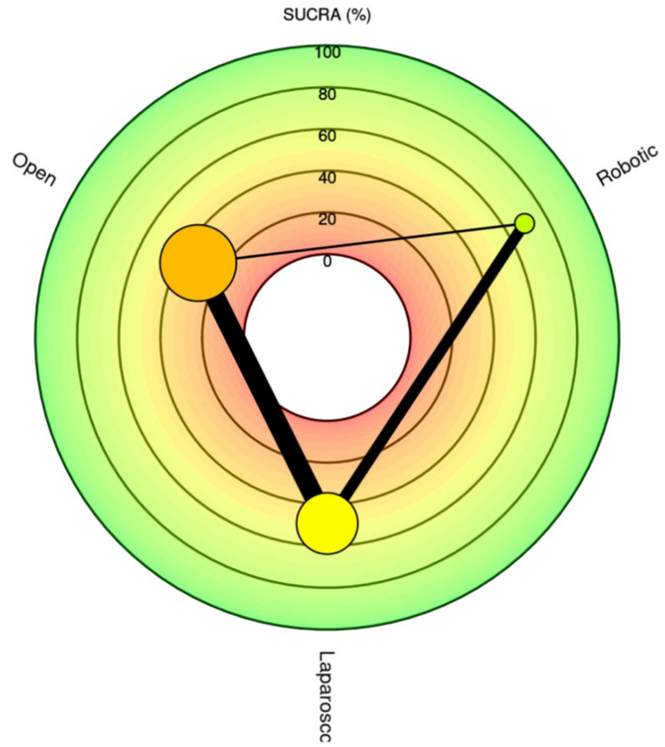

Supplementary Figure S31: Radial SUCRA plot: Outcomes cardiovascular diseases. Higher SUCRA values indicate better treatments; size of nodes represent number of participants and thickness of lines indicate number of trials conducted.

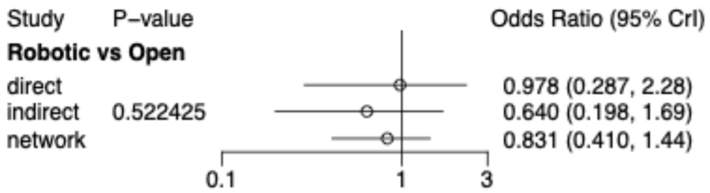

Supplementary Figure S32: Nodesplit model for the outcome operative duration.

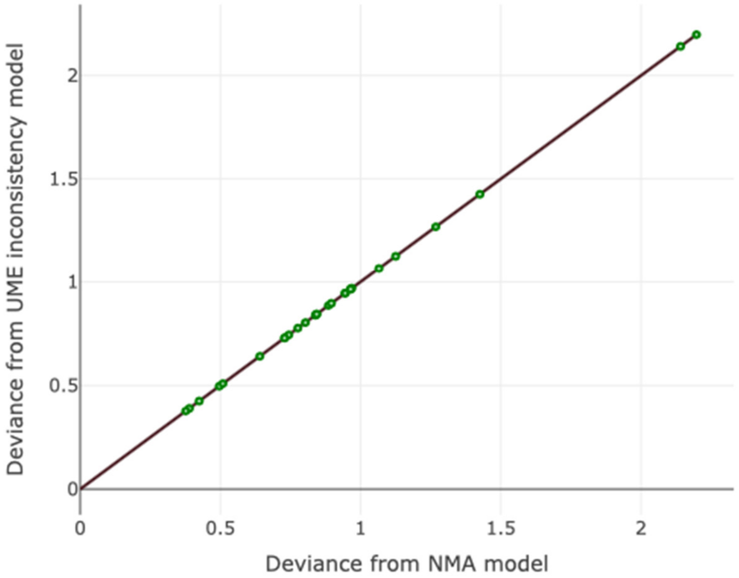

Supplementary Figure S33: Residual deviance from NMA model and UME inconsistency model for all studies.

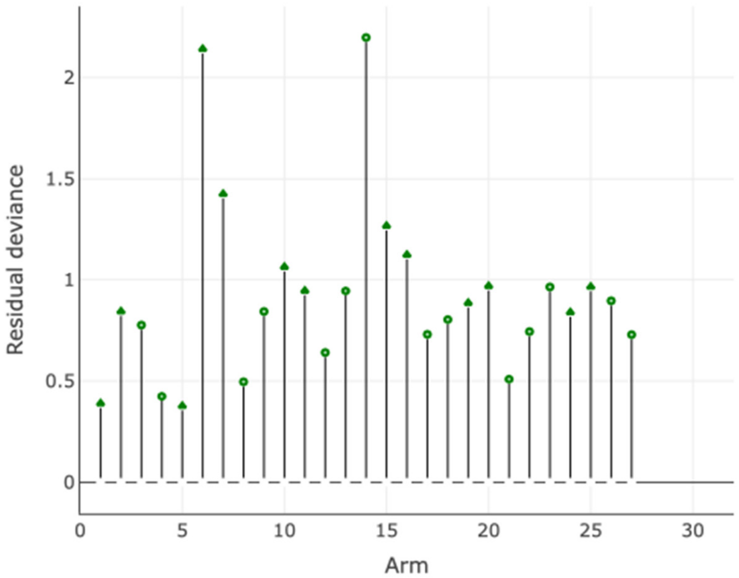

Supplementary Figure S34: Per-arm residual deviance for all studies. This stem plot represents the posterior residual deviance per study arm.

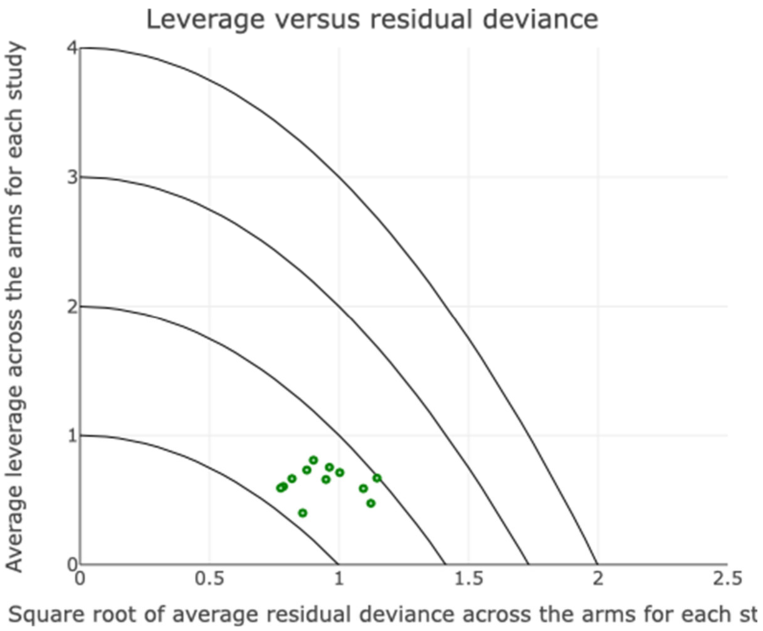

Supplementary Figure S35: Leverage plot for all studies.

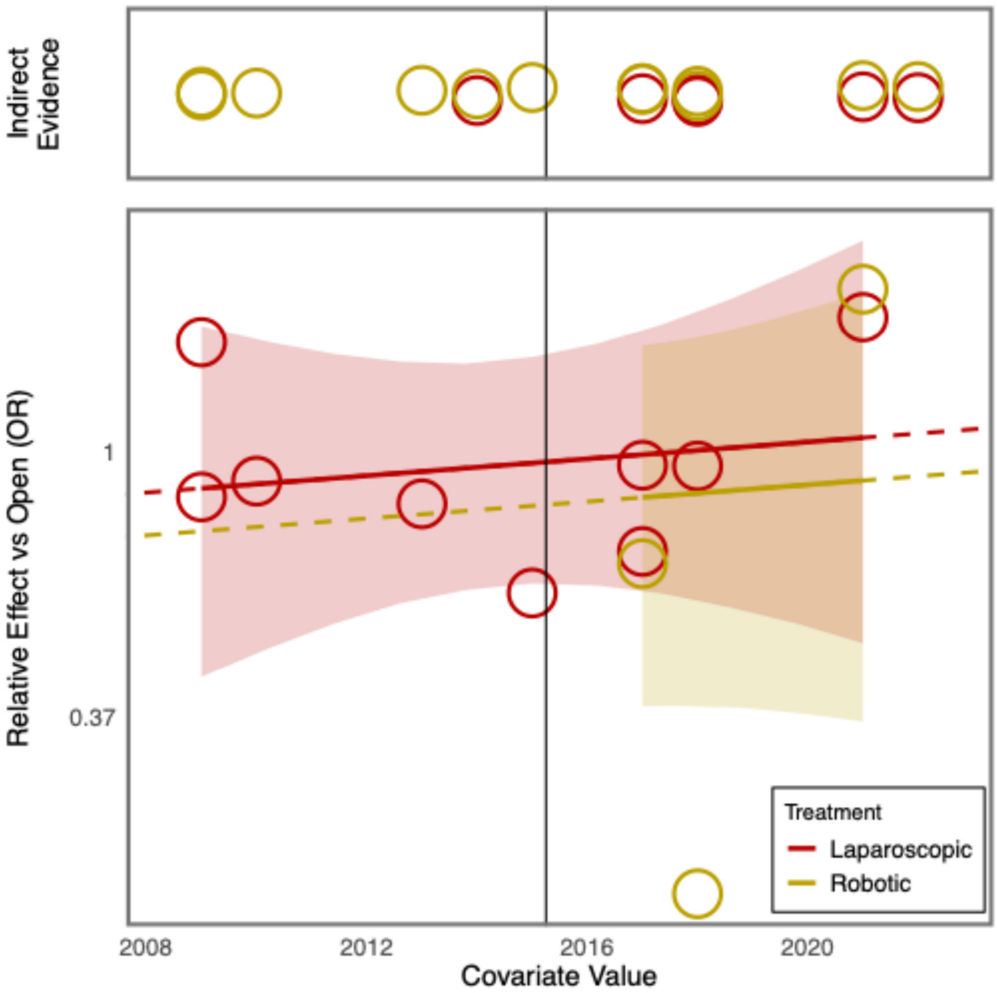

Supplementary Figure S36: Regression plot for the outcome cardiovascular diseases having as covariate the year of study publication.

5. Operative time

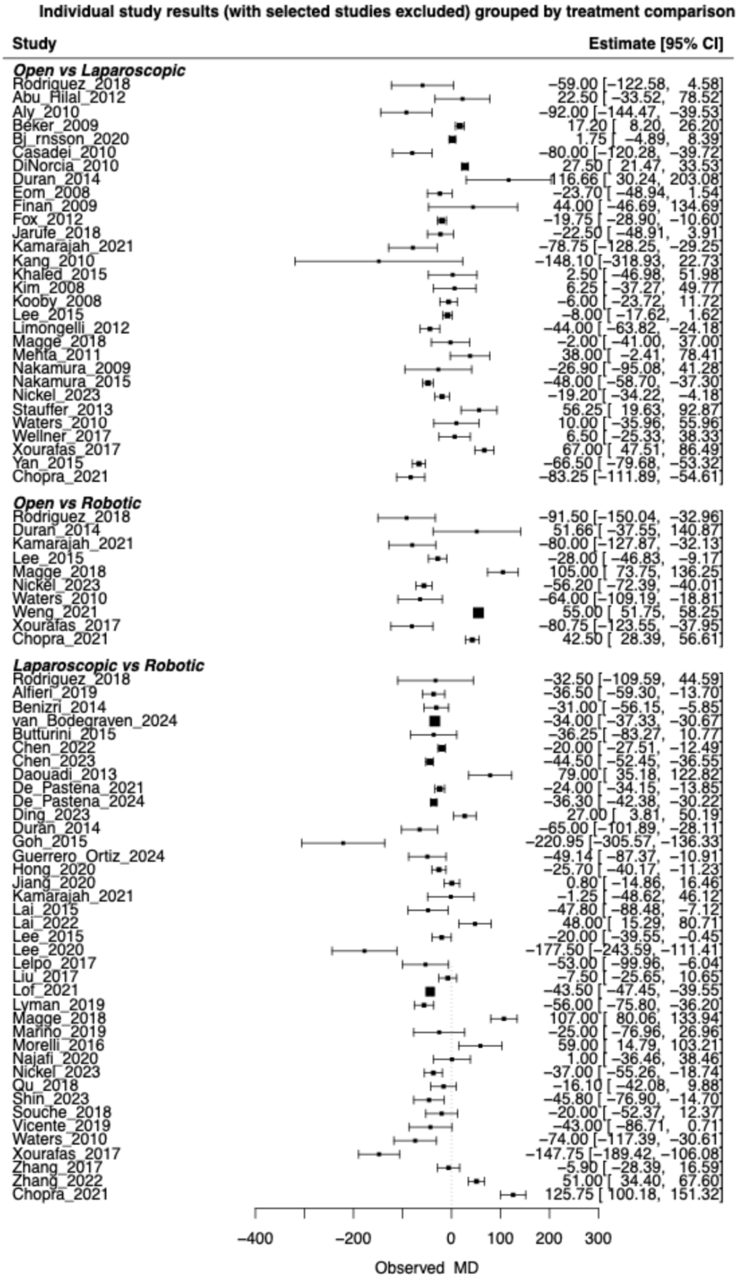

Supplementary Figure S37: Individual study results grouped by treatment comparison for the outcome operative time.

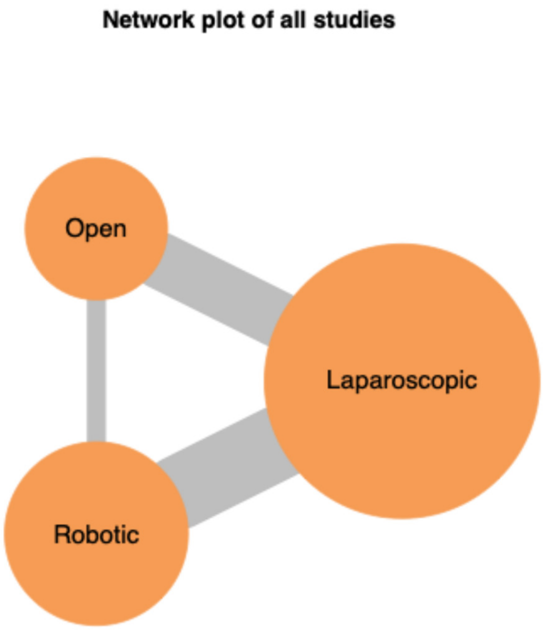

Supplementary Figure S38: Network plot of all studies for outcome operative time. The size of the nodes and thickness of edges represent the number of studies that examined a treatment and compared two given treatments respectively

.

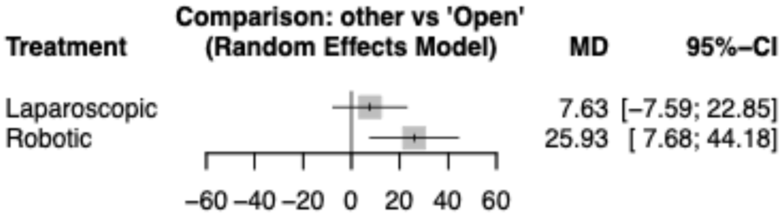

Supplementary Figure S39: Forest plot for outcome operative time. The size of the nodes and thickness of edges represent the number of studies that examined a treatment and compared two given treatments respectively.

Supplementary Table S10: Comparison of all treatment pairs related to outcome operative time. Treatments are ranked from best to worst along the leading diagonal. Above the leading diagonal are estimates from pairwise meta-analyses, below the leading diagonal are estimates from network meta-analyses. Relative treatment effects in ranked order for all studies

	**Open**	**Laparoscopic**	**Robotic**
**Open**	Open	-12.10 [-28.09; 3.89]	-12.20 [-39.77; 15.37]
**Laparoscopic**	-7.63 [-22.85; 7.59]	Laparoscopic	-20.82 [-34.43; -7.21]
**Robotic**	-25.93 [-44.18; -7.68]	-18.30 [-31.49; -5.12]	Robotic

Supplementary Table S11: Assessment of inconsistency for all studies related to outcome operative time.

	**Comparison**	**No.Studies**	**NMA**	**Direct**	**Indirect**	**Difference**	**Diff_95CI_lower**	**Diff_95CI_upper**	**pValue**
**1**	Laparoscopic:Open	30	7.63057203841852	12.1007915581876	-35.7965387734342	47.8973303316219	-4.43763996737038	100.232300630614	0.0728494516830057
**2**	Laparoscopic:Robotic	39	-18.3019383111563	-20.8212053430887	20.141979312067	-40.9631846551557	-95.8401371440765	13.913767833765	0.143460472484556
**3**	Robotic:Open	10	25.9325103495748	12.2002790921557	36.6451707549976	-24.4448916628419	-61.2233420304397	12.3335587047559	0.19267887385987

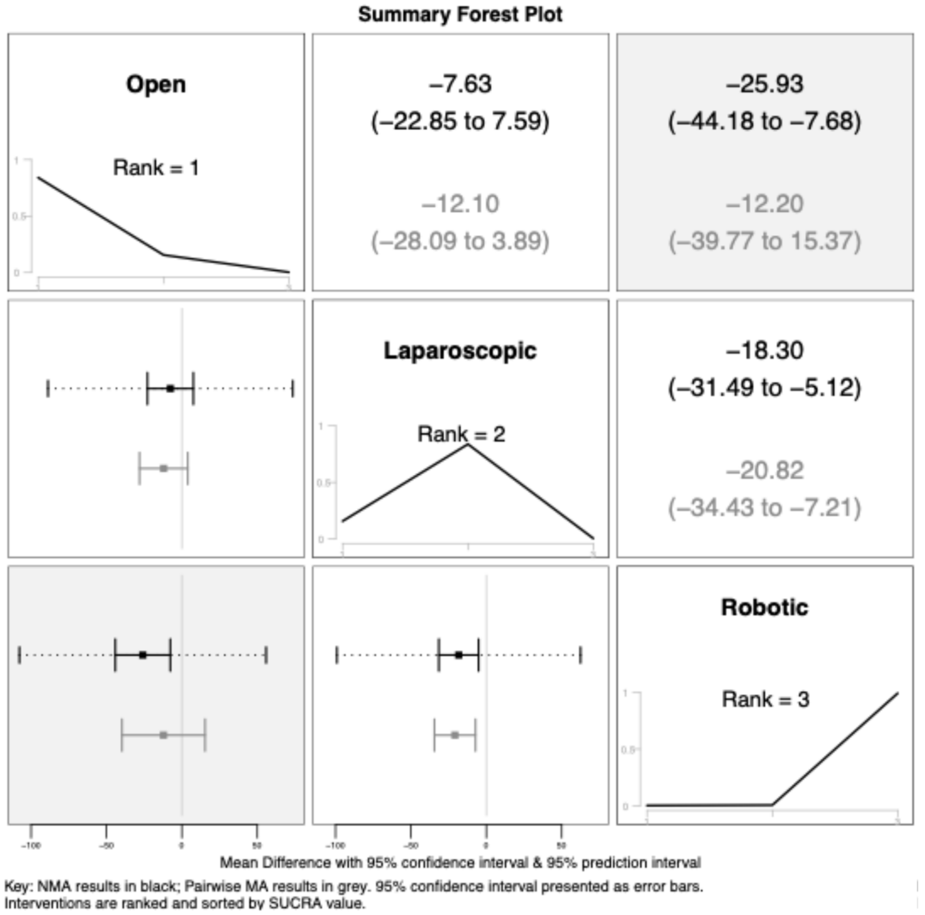

Supplementary Figure S40: Summary Forrest Plot for outcome operative time. Ranking of the interventions based on the SUCRA value.

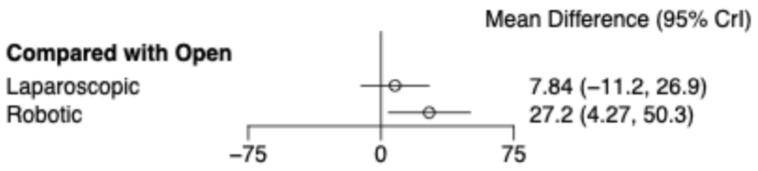

Supplementary Figure S41: Bayesian random effect consistency model forrest plot for outcome operative duration. Between studies standard deviation 51.49, 95% credible interval 41.93 to 63.28.

Supplementary Table S12: Treatment effects for all studies: comparison of all treatment pairs. Outcomes operative duration. Bayesian NMA.

	**Laparoscopic**	**Open**	**Robotic**
**Laparoscopic**	Laparoscopic	-7.84 (-26.94, 11.24)	19.32 (2.74, 36.27)
**Open**	7.84 (-11.24, 26.94)	Open	27.17 (4.27, 50.33)
**Robotic**	-19.32 (-36.27, -2.74)	-27.17 (-50.33, -4.27)	Robotic

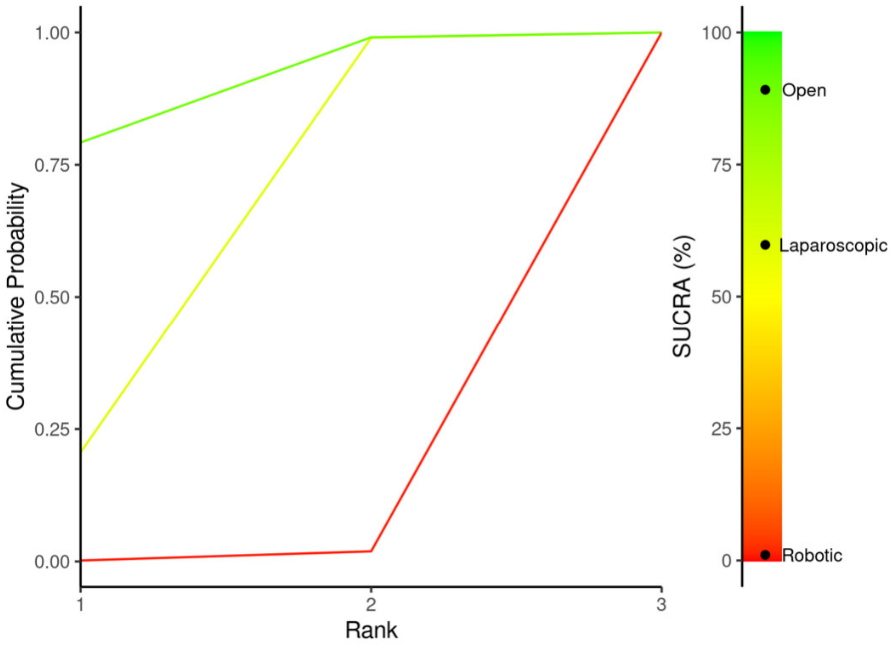

Supplementary Figure S42: Litmus Rank-O-Gram: Outcomes operative duration. Higher SUCRA (Surface Under the Cumulative Ranking Curve) values and cumulative ranking curves nearer the top left indicate better performance.

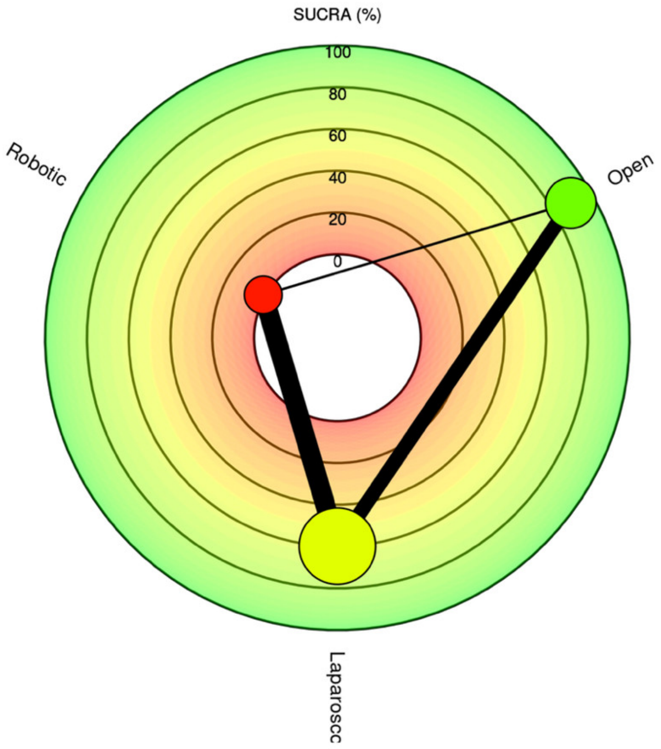

Supplementary Figure S43: Radial SUCRA plot: Outcomes operative time. Higher SUCRA values indicate better treatments; size of nodes represent number of participants and thickness of lines indicate number of trials conducted.

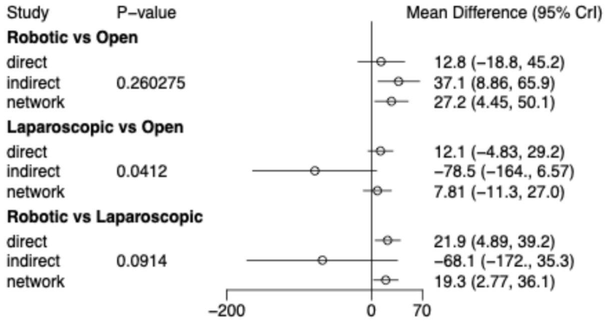

Supplementary Figure S44: Inconsistency test with nodesplitting model for all studies for outcome opertative durationl.

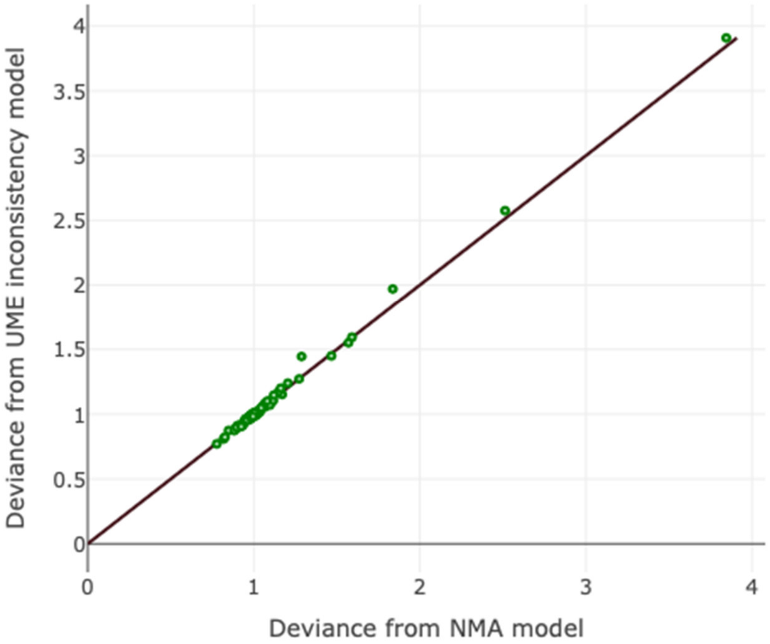

Supplementary Figure S45: Residual deviance from NMA model and UME inconsistency model for all studies.

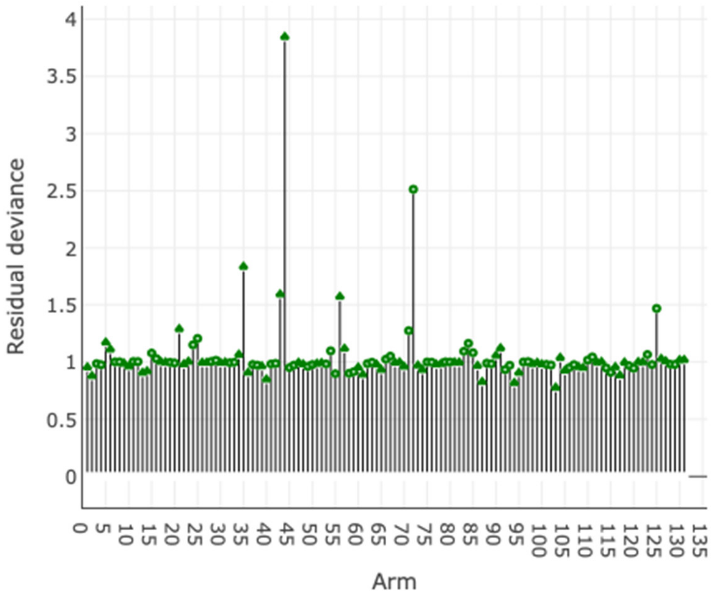

Supplementary Figure S46: Per-arm residual deviance for all studies. This stem plot represents the posterior residual deviance per study arm.

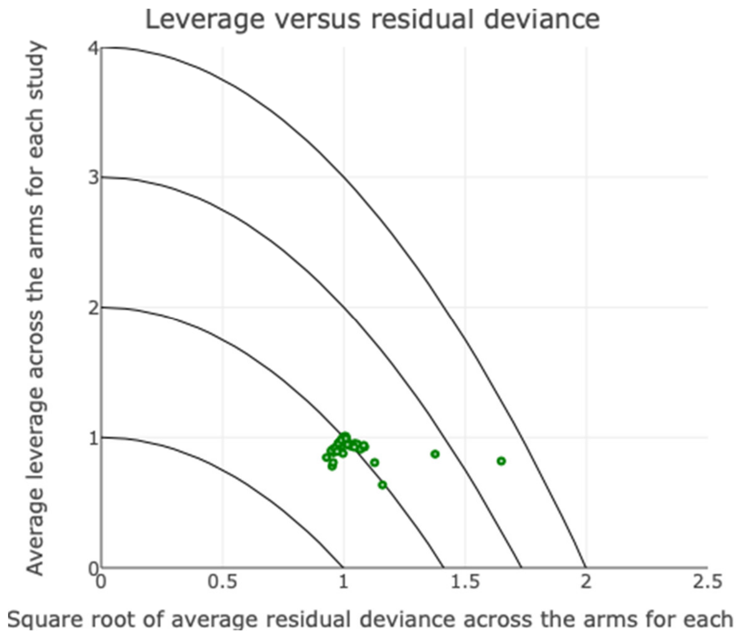

Supplementary Figure S47: Leverage plot for all studies.

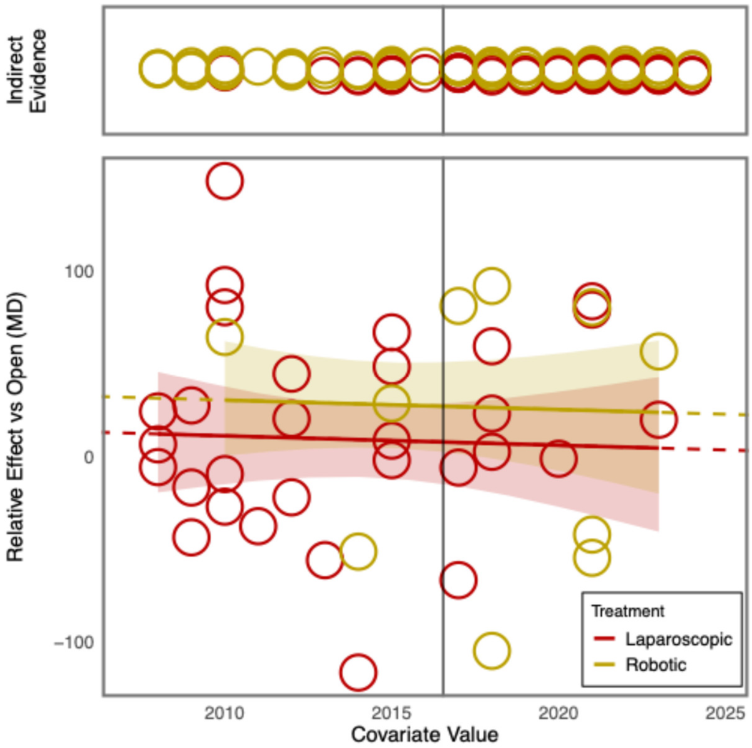

Supplementary Figure S48: Regression plot for the outcome operative duration having as covariate the year of study publication.

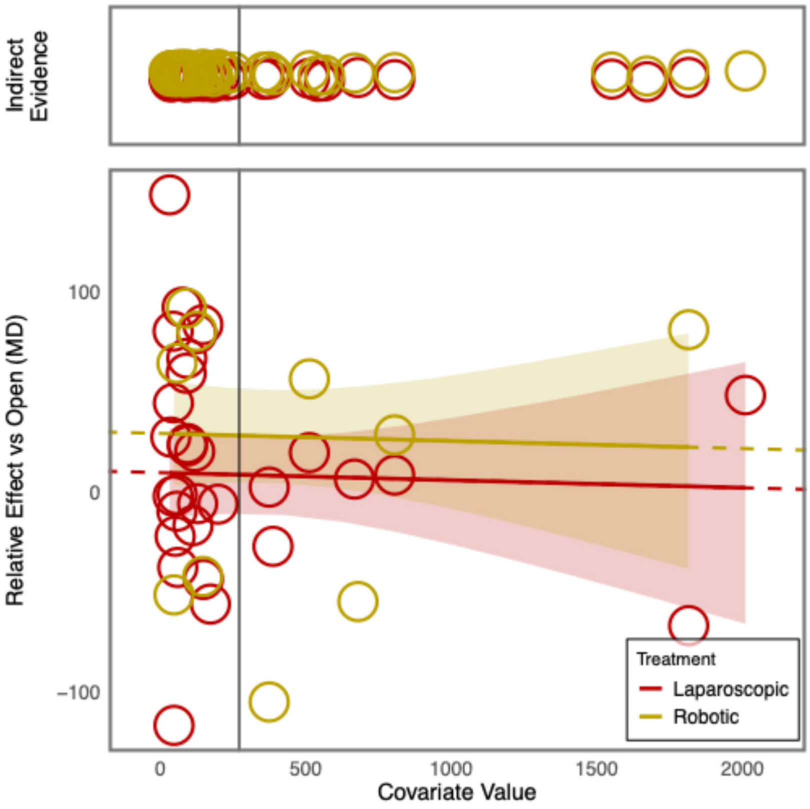

Supplementary Figure S49: Regression plot for the outcome operative duration having as covariate the number of patients in the included studies.

6. Conversion to open

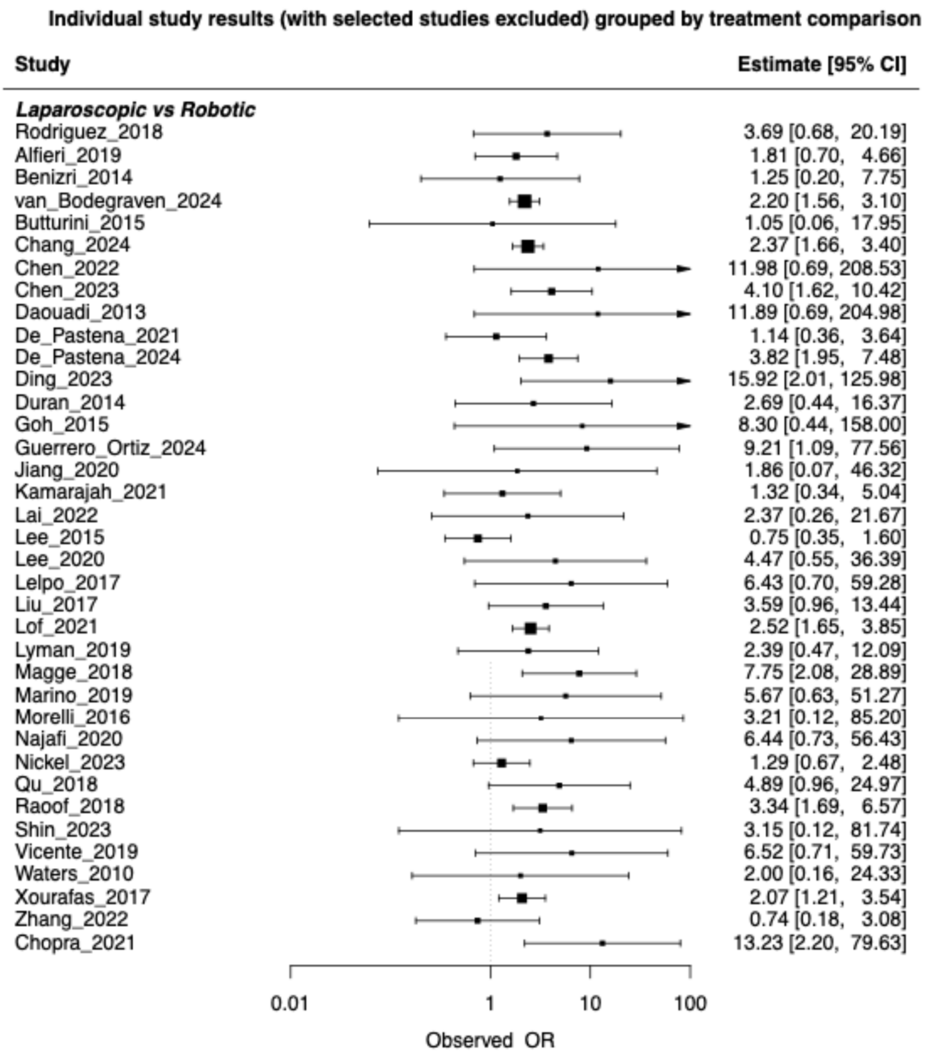

Supplementary Figure S50: Individual study results grouped by treatment comparison for the outcome conversions to open.

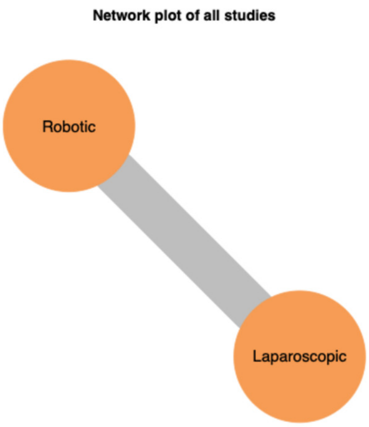

Supplementary Figure S51: Network plot of all studies for outcome conversions. The size of the nodes and thickness of edges represent the number of studies that examined a treatment and compared two given treatments respectively.

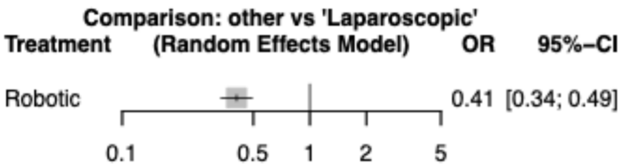

Supplementary Figure S52: Forest plot for outcome conversions. The size of the nodes and thickness of edges represent the number of studies that examined a treatment and compared two given treatments respectively. Between-study standard deviation (log-odds scale): 0.2 , Number of studies: 37 , Number of treatments: 2. All outcomes are versus the reference treatment: Laparoscopic

Supplementary Table S13: Comparison of all treatment pairs related to outcome conversions. Treatments are ranked from best to worst along the leading diagonal. Above the leading diagonal are estimates from pairwise meta-analyses, below the leading diagonal are estimates from network meta-analyses. Relative treatment effects in ranked order for all studies

	**Robotic**	**Laparoscopic**
**Robotic**	Robotic	0.41 [0.34; 0.49]
**Laparoscopic**	0.41 [0.34; 0.49]	Laparoscopic

Supplementary Table S14: Treatment effects for all studies: comparison of all treatment pairs. Outcomes conversions. Bayesian NMA.

	Laparoscopic	Robotic
**Laparoscopic**	Laparoscopic	0.35 (0.25, 0.44)
**Robotic**	2.88 (2.27, 4.03)	Robotic

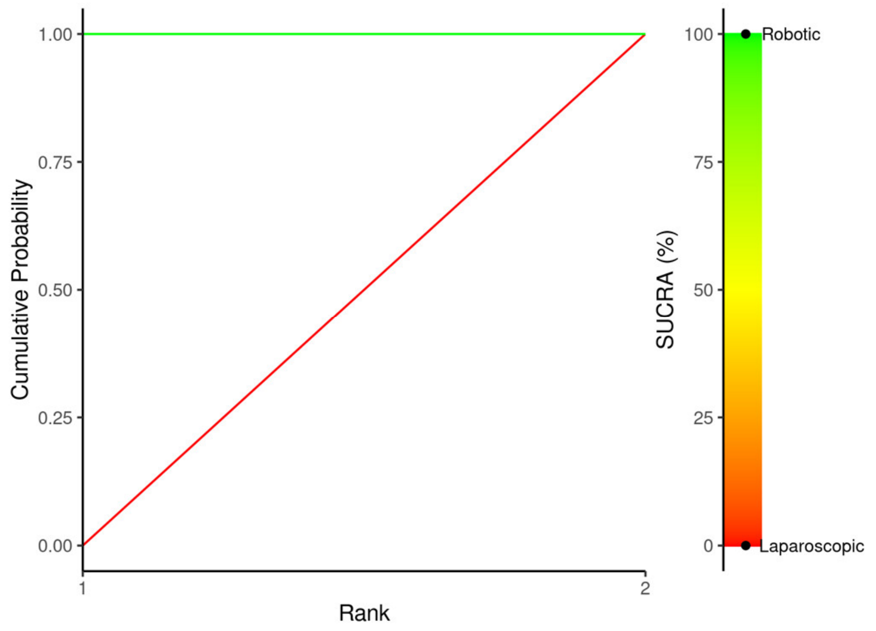

Supplementary Figure S53: Litmus Rank-O-Gram: Outcomes conversions. Higher SUCRA (Surface Under the Cumulative Ranking Curve) values and cumulative ranking curves nearer the top left indicate better performance.

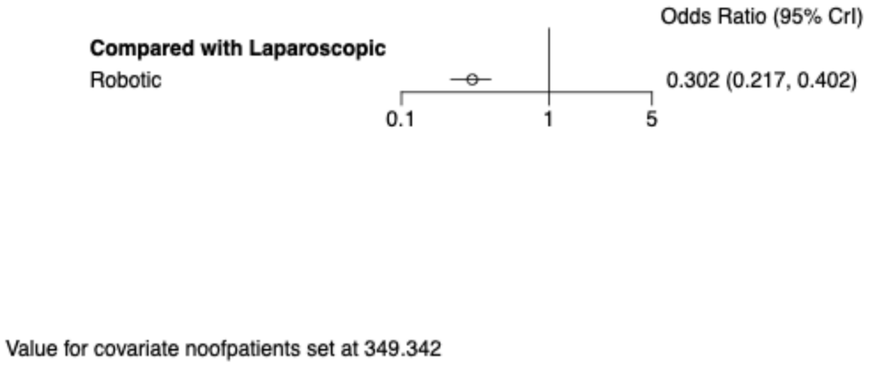

Supplementary Figure S54: Forrest plot of metaregression for the outcome conversions having as covariate the number of patients in the included studies. Between-study standard deviation (log-odds scale): 0.39 . 95% credible interval: 0.02 , 0.81 .

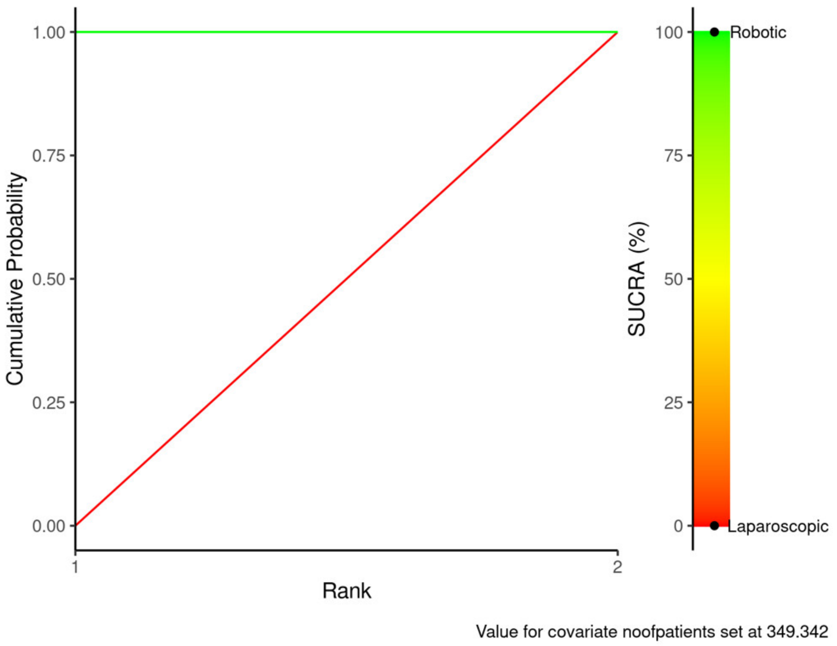

Supplementary Figure S55: : Litmus Rank-O-Gram: Outcomes conversions. Higher SUCRA (Surface Under the Cumulative Ranking Curve) values and cumulative ranking curves nearer the top left indicate better performance.

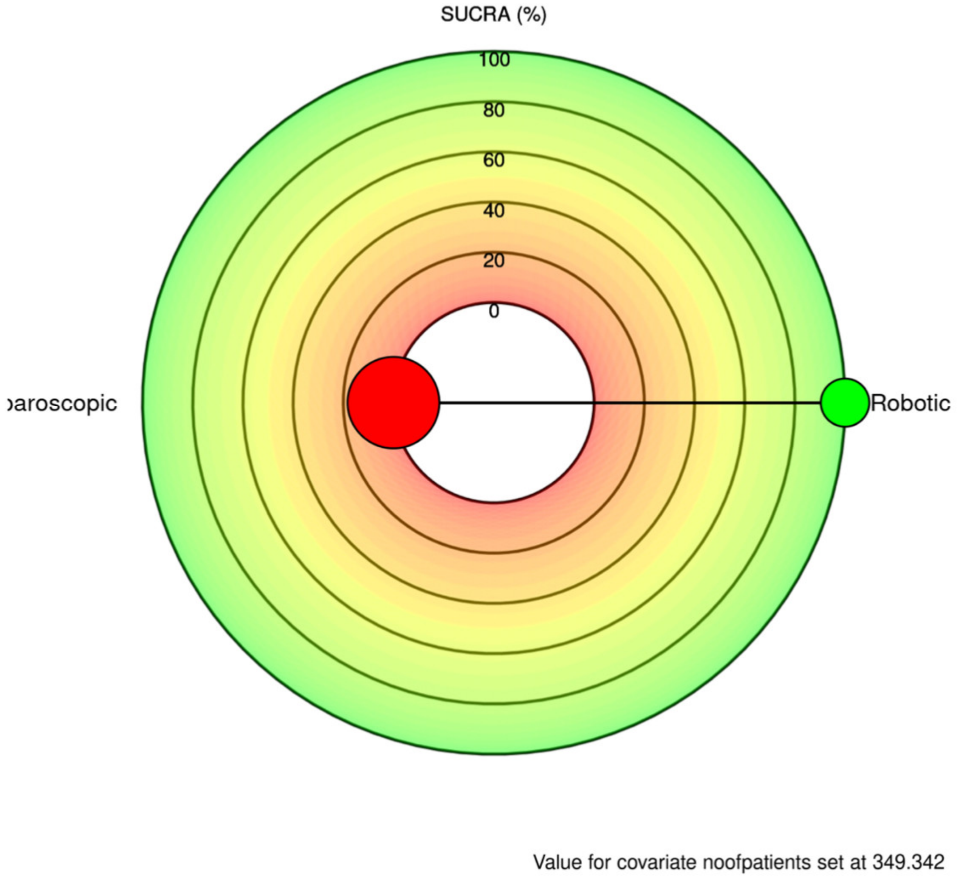

Supplementary Figure S56: Radial SUCRA plot: Outcomes conversions. Higher SUCRA values indicate better treatments; size of nodes represent number of participants and thickness of lines indicate number of trials conducted.

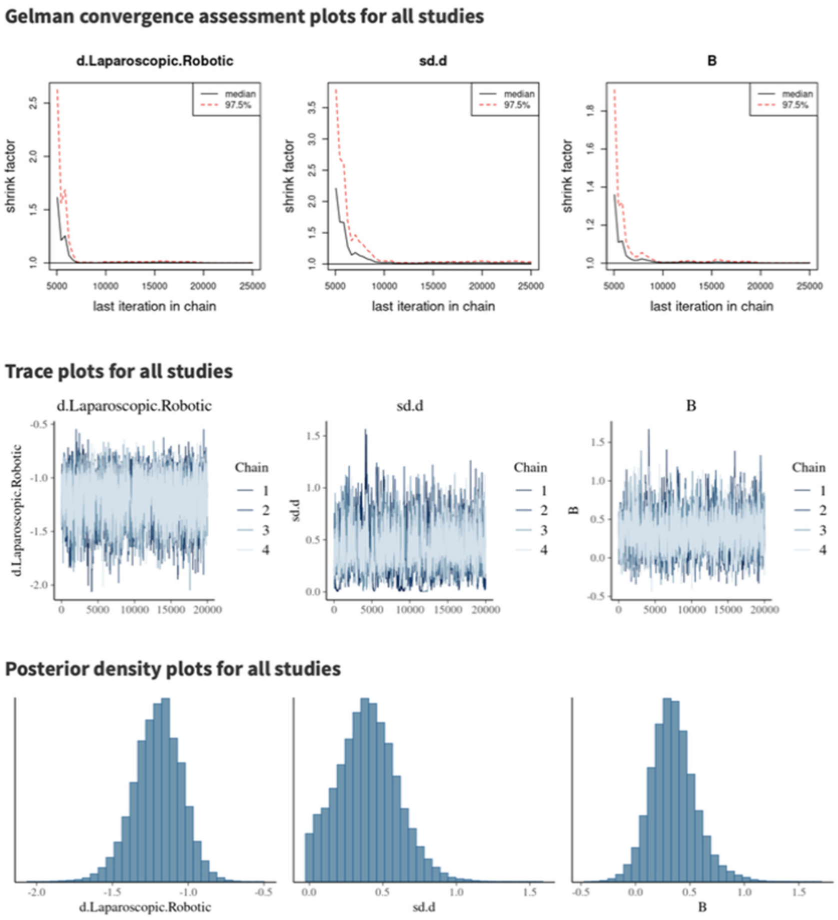

Supplementary Figure S57: Convergence assessment plots for the outcomes conversion.

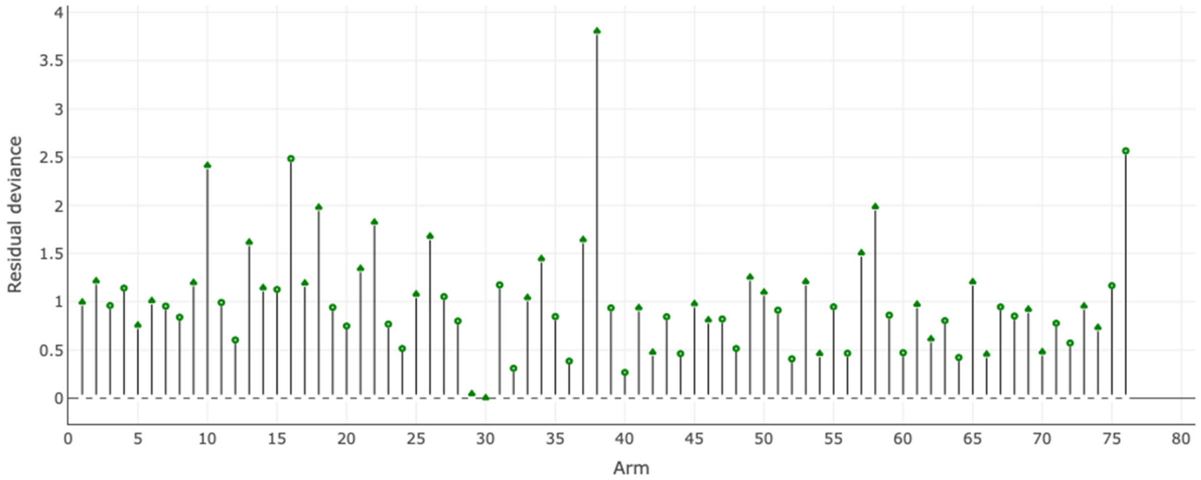

Supplementary Figure S58: Per-arm residual deviance for all studies. This stem plot represents the posterior residual deviance per study arm.

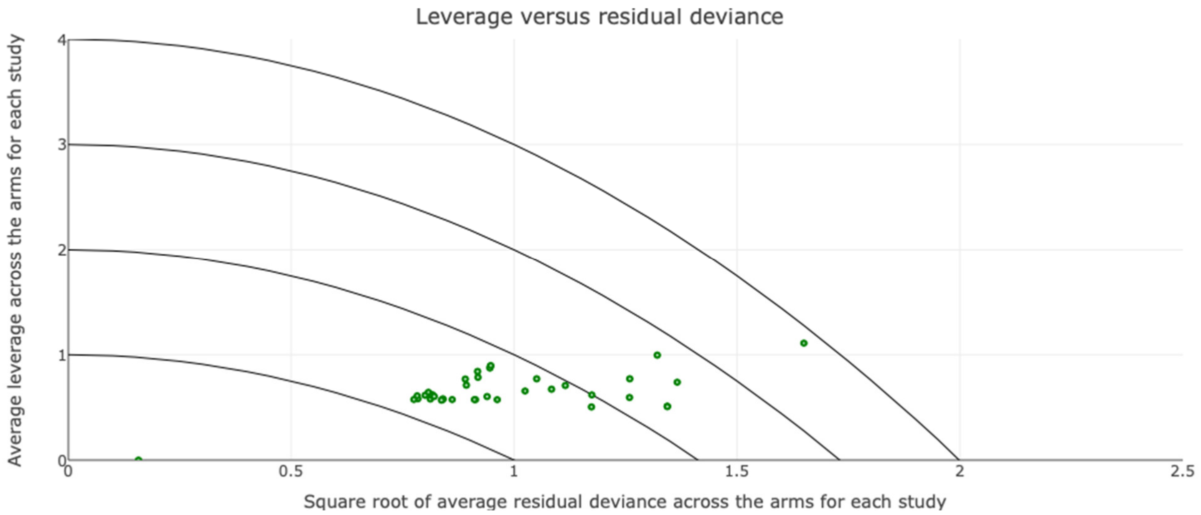

Supplementary Figure S59: Leverage plot for all studies.

7. Intraoperative blood loss

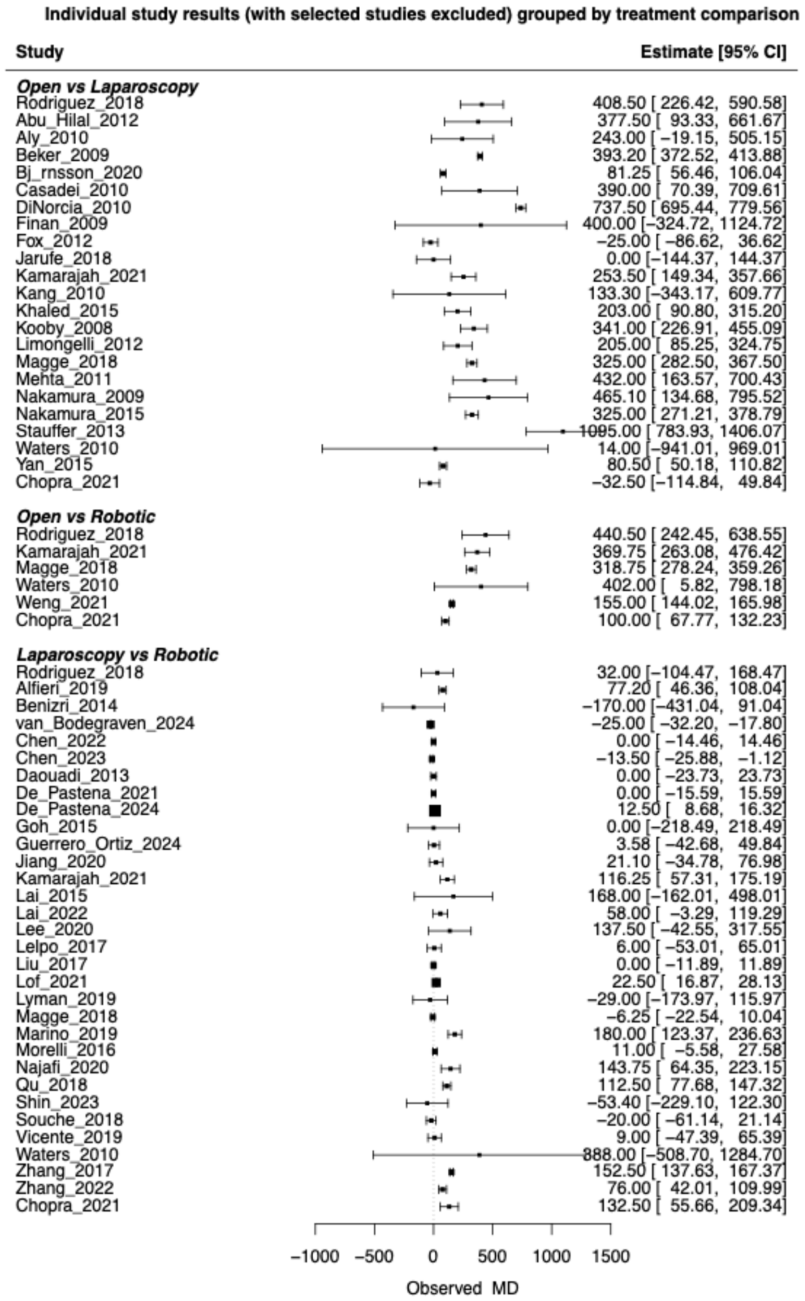

Supplementary Figure S60: Individual study results grouped by treatment comparison for the outcome Intraoperative blood loss.

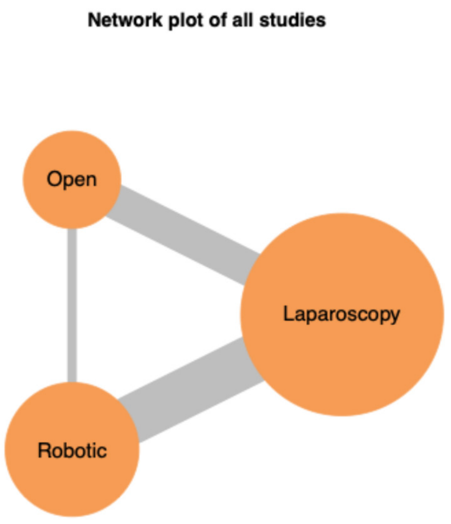

Supplementary Figure S61: Network plot of all studies for outcome Intraoperative bleeding. The size of the nodes and thickness of edges represent the number of studies that examined a treatment and compared two given treatments respectively.

Frequentist NMA

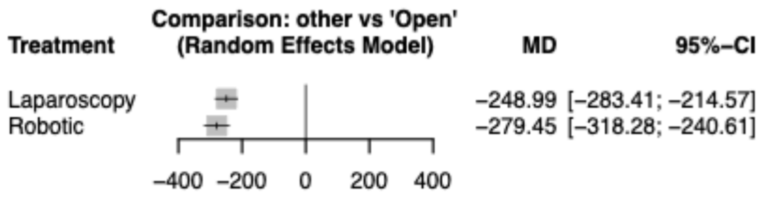

Supplementary Figure S62: Forest plot for outcome Intraoperative bleeding. The size of the nodes and thickness of edges represent the number of studies that examined a treatment and compared two given treatments respectively.

Supplementary Table S15: Comparison of all treatment pairs related to outcome intraoperative bleeding. Treatments are ranked from best to worst along the leading diagonal. Above the leading diagonal are estimates from pairwise meta-analyses, below the leading diagonal are estimates from network meta-analyses. Relative treatment effects in ranked order for all studies

	Robotic	Laparoscopy	Open
**Robotic**	Robotic	-38.79 [-63.70; -13.88]	-239.38 [-302.09; -176.67]
**Laparoscopy**	-30.45 [-54.66; -6.25]	Laparoscopy	-260.63 [-297.53; -223.74]
**Open**	-279.45 [-318.28; -240.61]	-248.99 [-283.41; -214.57]	Open

Supplementary Table S16: Assessment of inconsistency for all studies related to outcome Intraoperative bleeding.

	Comparison	No.Studies	NMA	Direct	Indirect	Difference	Diff_95CI_lower	Diff_95CI_upper	pValue
1	Laparoscopy:Open	23	-248.992286829271	-260.632974653398	-170.790127240655	-89.8428474127432	-192.342970684633	12.6572758591469	0.0858081232482131
2	Laparoscopy:Robotic	32	30.453697002749	38.7897986061359	-110.135388602818	148.925187208954	43.6274860770919	254.222888340816	0.00557085249117154
3	Robotic:Open	6	-279.44598383202	-239.383118954338	-304.370743338983	64.9876243846459	-14.8806189013062	144.855867670598	0.110758286671654

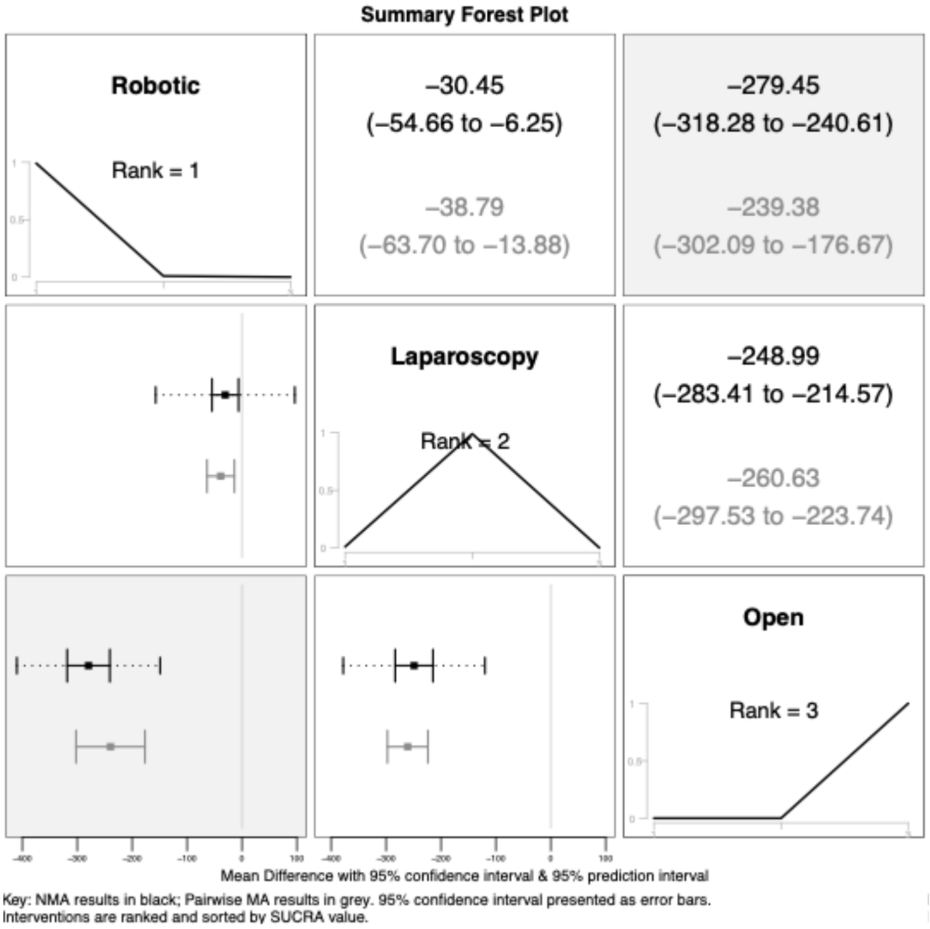

Supplementary Figure S63: Summary Forrest Plot for outcome Intraoperative bleeding. Ranking of the interventions based on the SUCRA value.

Bayesian NMA

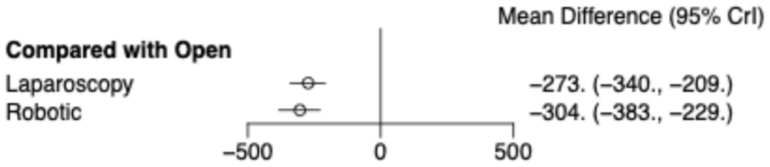

Supplementary Figure S64: Bayesian random effect consistency model forrest plot for outcome Intraoperative bleeding. Between studies standard deviation 139.19, 95% credible interval 109.56 to 177.58.

Supplementary Table S17: Treatment effects for all studies: comparison of all treatment pairs. Outcomes Intraoperative bleeding. Bayesian NMA.

	Laparoscopy	Open	Robotic
**Laparoscopy**	Laparoscopy	272.89 (209.01, 340.15)	-31.17 (-81.92, 19.44)
**Open**	-272.89 (-340.15, -209.01)	Open	-303.98 (-382.8, -229.26)
**Robotic**	31.17 (-19.44, 81.92)	303.98 (229.26, 382.8)	Robotic

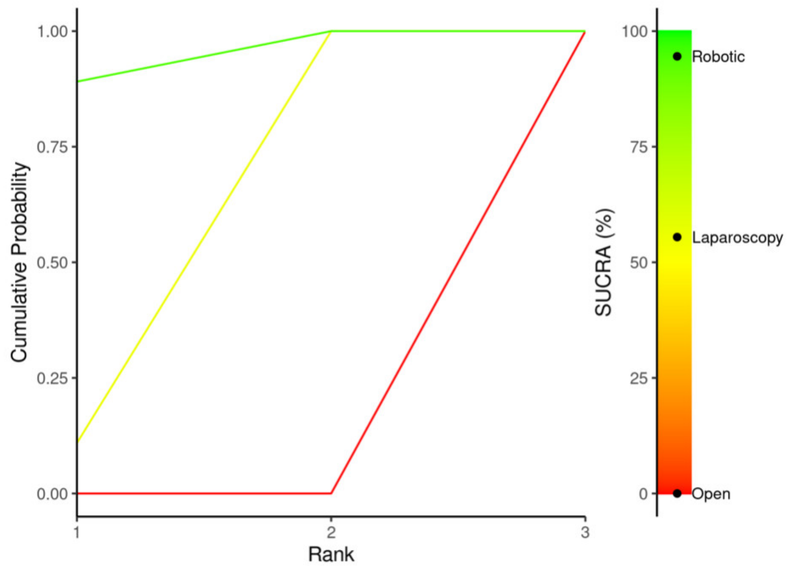

Supplementary Figure S65: Litmus Rank-O-Gram: Outcomes Intraoperative bleeding. Higher SUCRA (Surface Under the Cumulative Ranking Curve) values and cumulative ranking curves nearer the top left indicate better performance.

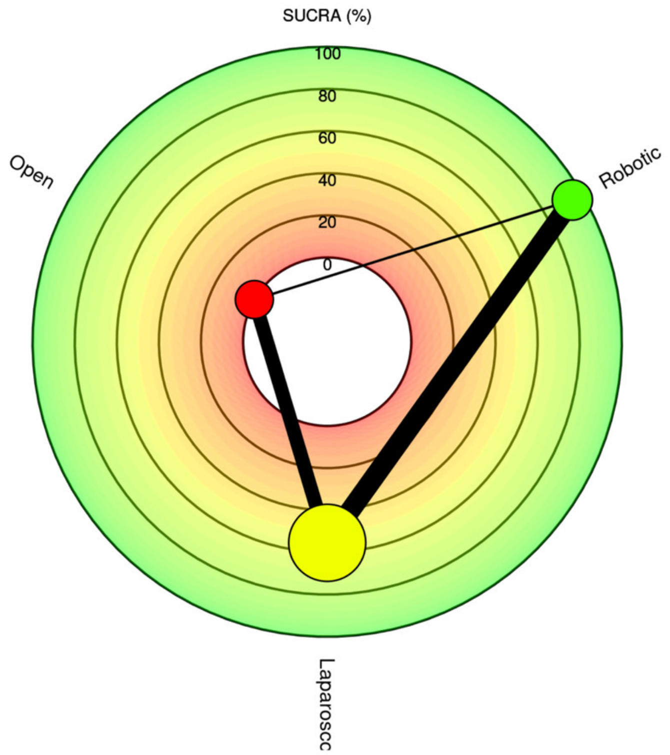

Supplementary Figure S66: Radial SUCRA plot: Outcomes Intraoperative bleeding. Higher SUCRA values indicate better treatments; size of nodes represent number of participants and thickness of lines indicate number of trials conducted.

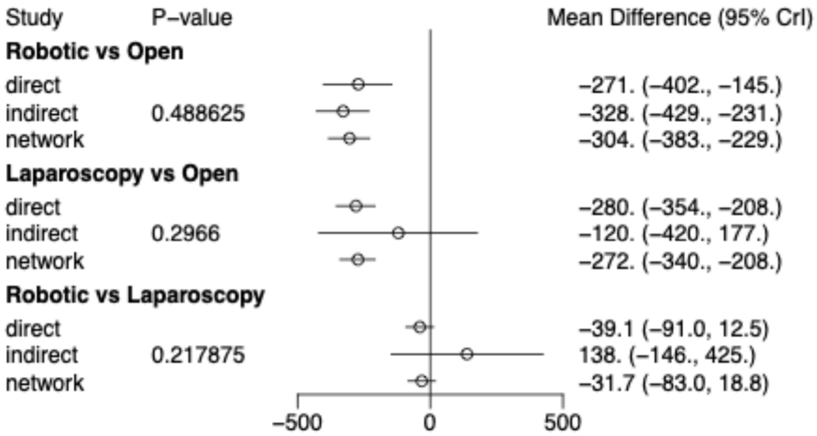

Supplementary Figure S67: The nodesplit model for the Bayesian NMA.

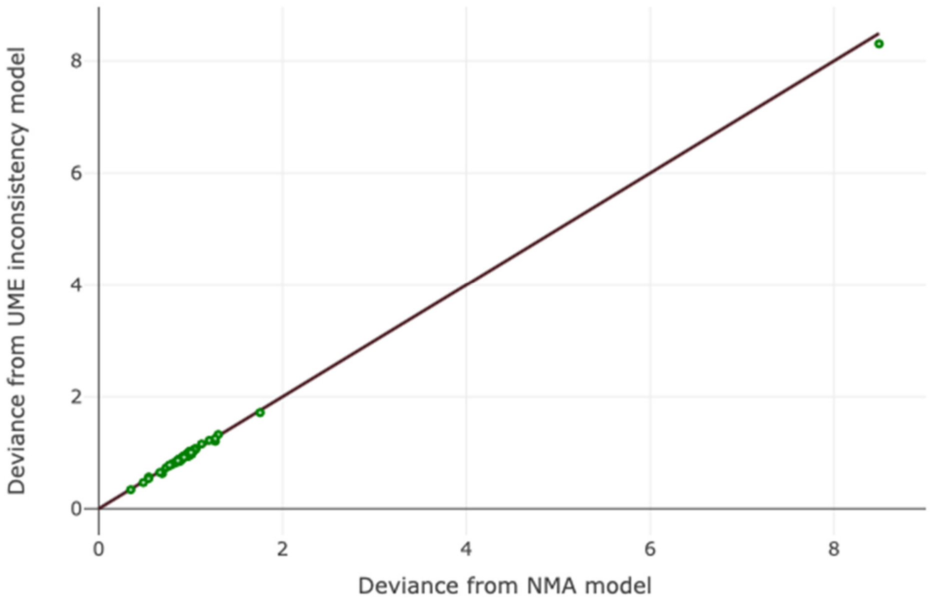

Supplementary Figure S68: Residual deviance from NMA model and UME inconsistency model for all studies.

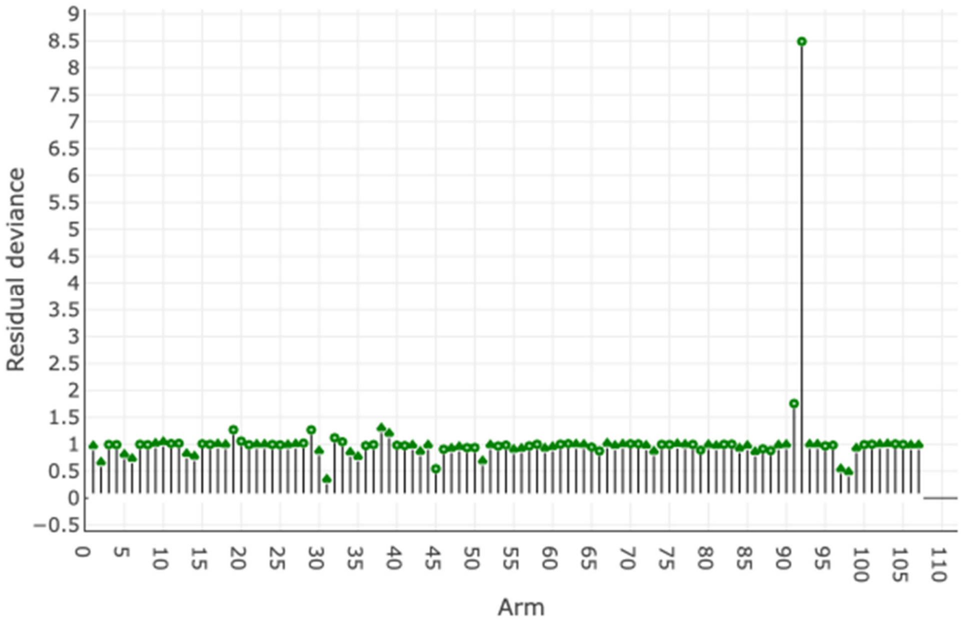

Supplementary Figure S69: Per-arm residual deviance for all studies. This stem plot represents the posterior residual deviance per study arm.

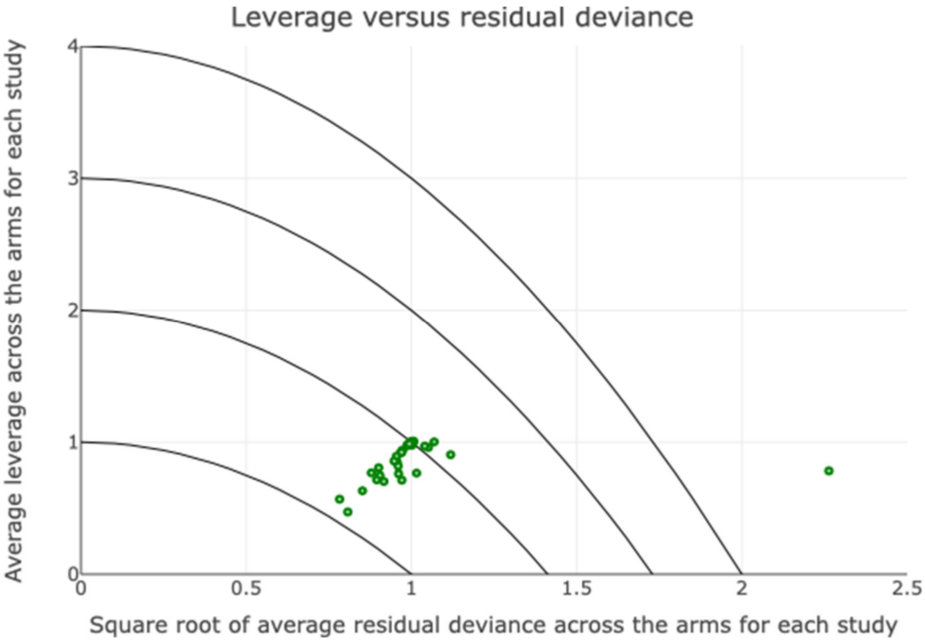

Supplementary Figure S70: Leverage plot for all studies.

Meta-regression

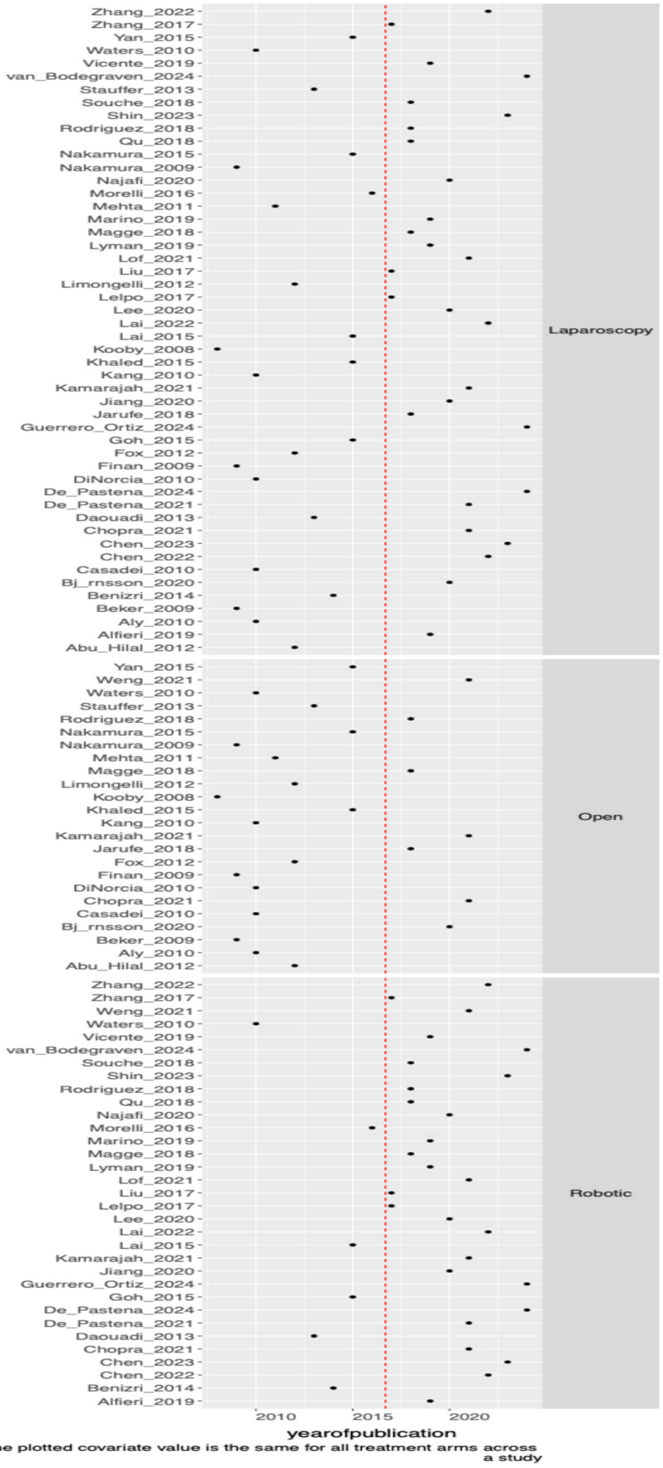

Supplementary Figure S71: The summary characteristic plot having as co-variate year of publication across al treatment arms.

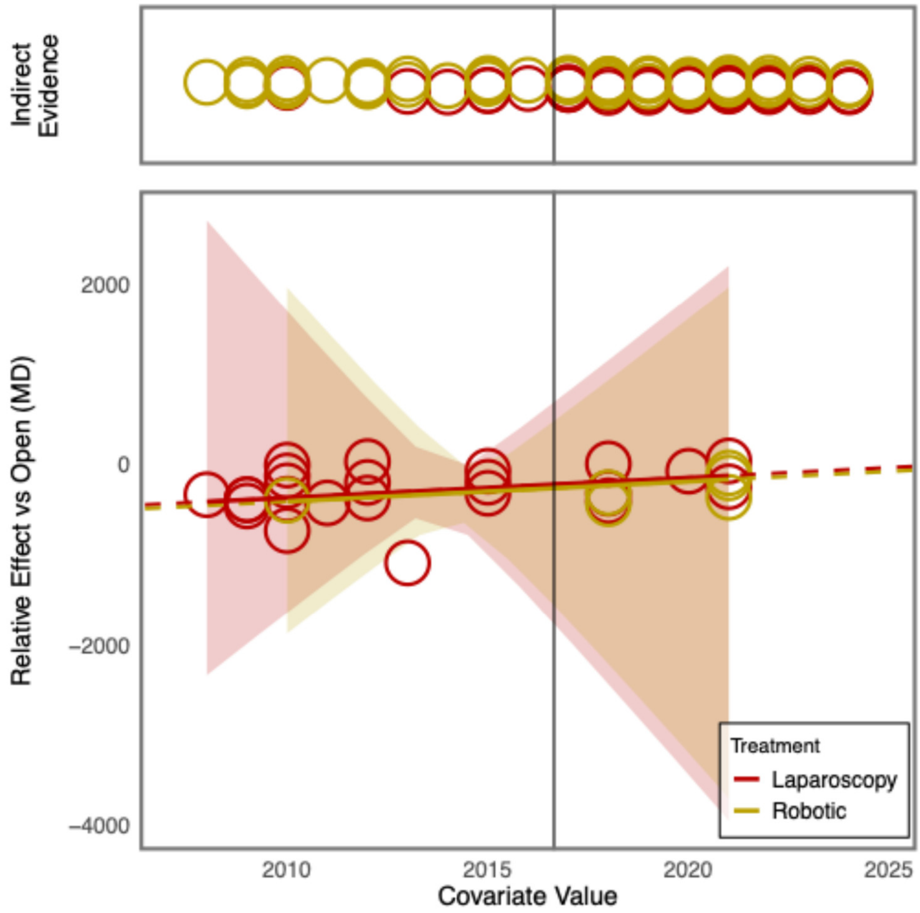

Supplementary Figure S72: Metaregression for the outcome Intraoperative bleeding having as covariate the year of study. publication.

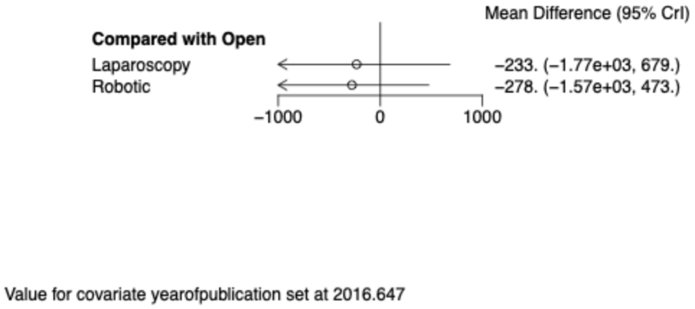

Supplementary Figure S73: Forrest plot of metaregression for the outcome intraoperative bleeding having as covariate the year of the study publication.

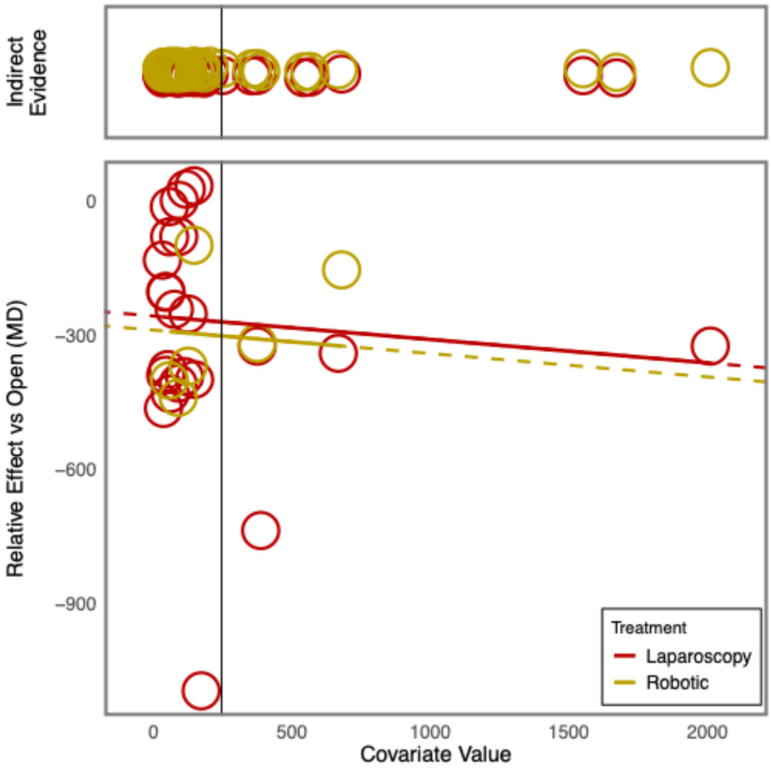

Supplementary Figure S74: Metaregression for the outcome Intraoperative bleeding having as covariate the number of patients in the included studies.

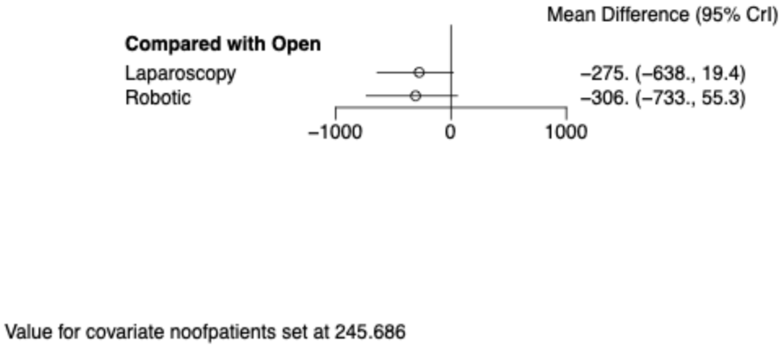

Supplementary Figure S75: Forrest plot of metaregression for the outcome intraoperative bleeding having as covariate the number of patients in the included studies.

8. Intraoperative bleeding more than 500 ml

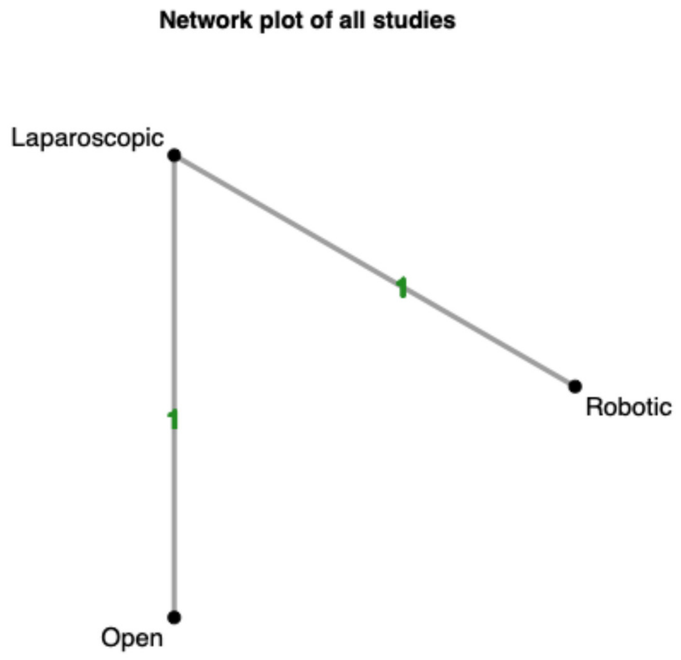

Supplementary Figure S76: Network plot of all studies for outcome intraoperative bleeding more 500 ml. The size of the nodes and thickness of edges represent the number of studies that examined a treatment and compared two given treatments respectively.

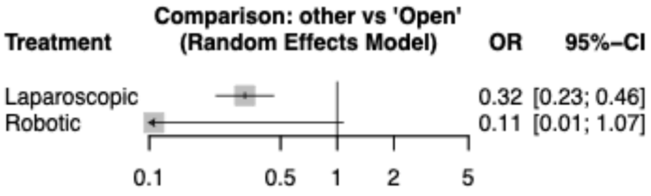

Supplementary Figure S77. Forest plot for outcome intraoperative bleeding more 500 ml.

Supplementary Table S18: Comparison of all treatment pairs related to outcome intraoperative bleeding more 500 ml. Treatments are ranked from best to worst along the leading diagonal. Above the leading diagonal are estimates from pairwise meta-analyses, below the leading diagonal are estimates from network meta-analyses. Relative treatment effects in ranked order for all studies

	Robotic	Laparoscopic	Open
**Robotic**	Robotic	0.33 [0.03; 3.24]	.
**Laparoscopic**	0.33 [0.03; 3.24]	Laparoscopic	0.32 [0.23; 0.46]
**Open**	0.11 [0.01; 1.07]	0.32 [0.23; 0.46]	Open

Supplementary Table S19: Assessment of inconsistency for all studies related to outcome intraoperative bleeding more 500 ml.

	Comparison	No.Studies	NMA	Direct	Indirect	Difference	Diff_95CI_lower	Diff_95CI_upper	pValue
1	Laparoscopic:Open	1	-1.13060420374009	-1.13060420374009	NA	NA	NA	NA	NA
2	Laparoscopic:Robotic	1	1.11541940698449	1.11541940698449	NA	NA	NA	NA	NA
3	Robotic:Open	0	-2.24602361072458	NA	-2.24602361072458	NA	NA	NA	NA

9. Number of patients receiving transfusions

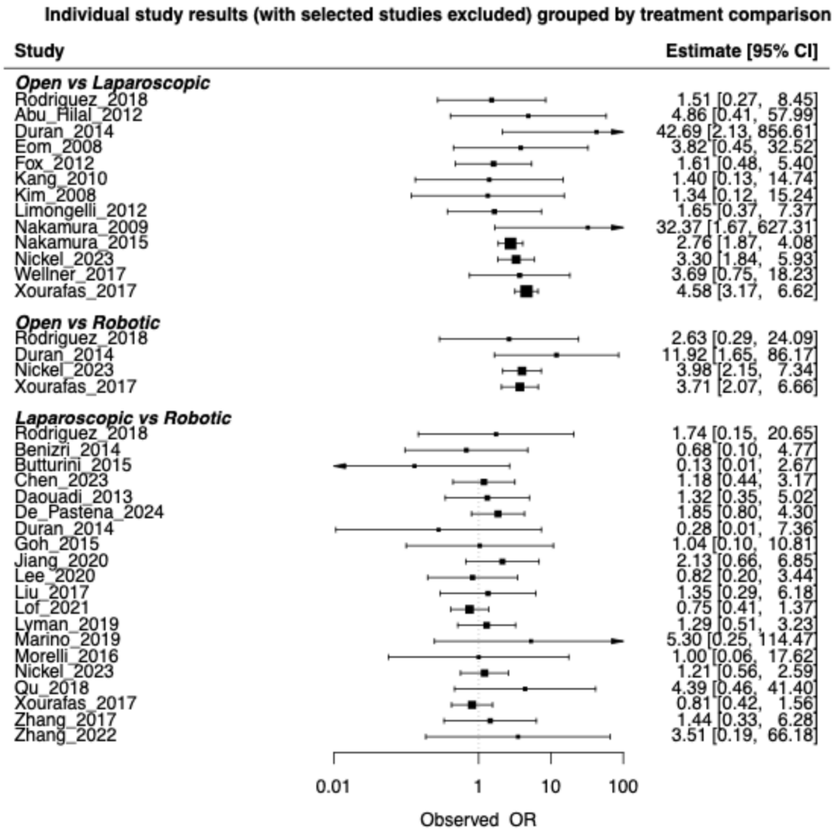

Supplementary Figure S78: Individual study results grouped by treatment comparison for the outcome patients receiving blood transfusion.

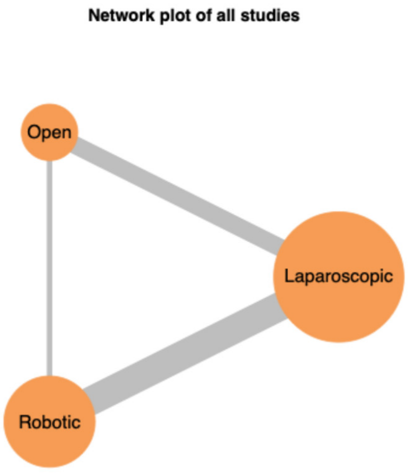

Supplementary Figure S79: Network plot of all studies for outcome patients receiving blood transfusion. The size of the nodes and thickness of edges represent the number of studies that examined a treatment and compared two given treatments respectively.

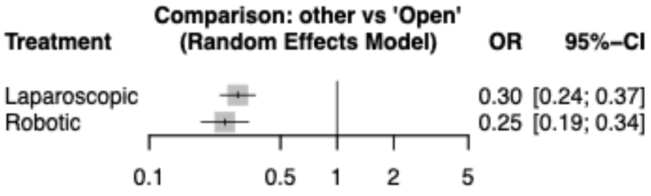

Supplementary Figure S80: Forest plot for outcome Number of patients receiving blood transfusions.

Supplementary Table S20: Comparison of all treatment pairs related to outcome number of patients receiving transfusions. Treatments are ranked from best to worst along the leading diagonal. Above the leading diagonal are estimates from pairwise meta-analyses, below the leading diagonal are estimates from network meta-analyses. Relative treatment effects in ranked order for all studies

	Robotic	Laparoscopic	Open
**Robotic**	Robotic	0.90 [0.69; 1.17]	0.25 [0.17; 0.38]
**Laparoscopic**	0.85 [0.66; 1.10]	Laparoscopic	0.29 [0.23; 0.37]
**Open**	0.25 [0.19; 0.34]	0.30 [0.24; 0.37]	Open

Supplementary Table S21: Assessment of inconsistency for all studies related to outcome number of patients with blood transfusions.

	Comparison	No.Studies	NMA	Direct	Indirect	Difference	Diff_95CI_lower	Diff_95CI_upper	pValue
1	Laparoscopic:Open	13	-1.21714010979183	-1.22278490114182	-1.17274006277716	-0.0500448383646597	-0.721427925317185	0.621338248587865	0.883845959949681
2	Laparoscopic:Robotic	20	0.157897493463074	0.107548647007187	1.03278155891915	-0.925232911911965	-2.05326846279931	0.202802638975376	0.107924126915767
3	Robotic:Open	4	-1.3750376032549	-1.37970398196641	-1.37034216051701	-0.00936182144940401	-0.585382703017054	0.566659060118246	0.9745881175923

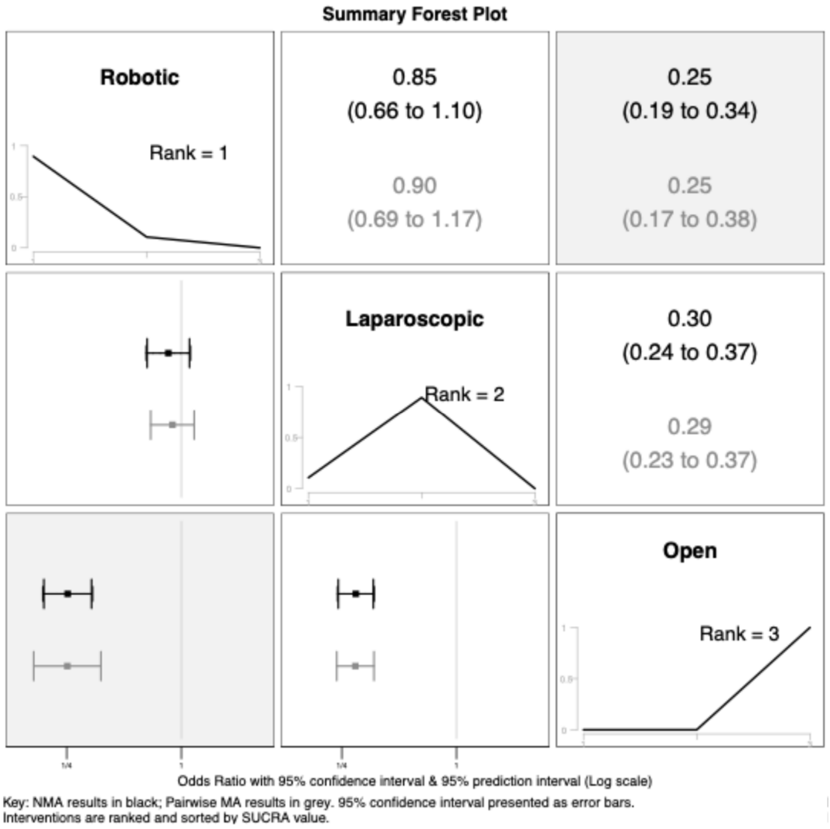

Supplementary Figure S81: Summary Forrest Plot for outcome number of patients having blood tranfusuions. Ranking of the interventions based on the SUCRA value.

10.The quantity of blood tranfusion

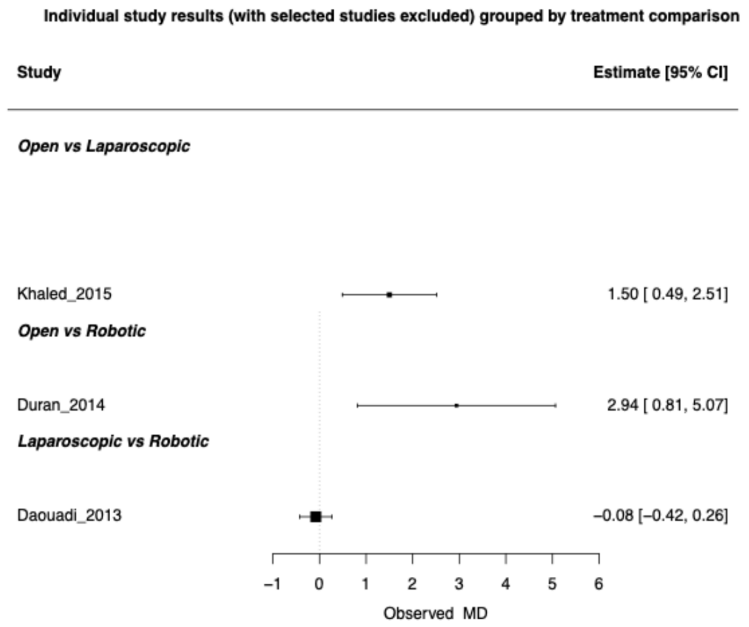

Supplementary Figure S82: Individual study results grouped by treatment comparison for the outcome quantity blood transfused.

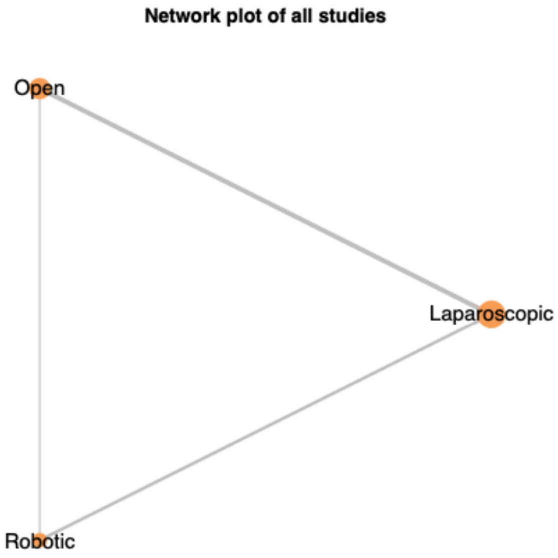

Supplementary Figure S83: Network plot of all studies for outcome quantity of blood transfused. The size of the nodes and thickness of edges represent the number of studies that examined a treatment and compared two given treatments respectively.

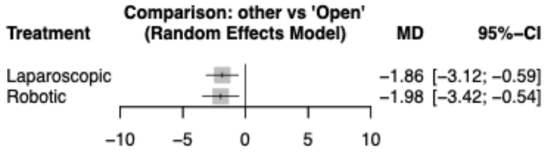

Supplementary Figure S84: Forest plot for outcome quantity of blood transfused. Between-study standard deviation: 0.53 , Number of studies: 3, Number of treatments: 3. All outcomes are versus the reference treatment: Open.

Supplementary Table S22: Comparison of all treatment pairs related to outcome quantity of blood transfused. Treatments are ranked from best to worst along the leading diagonal. Above the leading diagonal are estimates from pairwise meta-analyses, below the leading diagonal are estimates from network meta-analyses. Relative treatment effects in ranked order for all studies.

	Robotic	Laparoscopic	Open
**Robotic**	Robotic	0.08 [-1.01; 1.17]	-2.94 [-5.30; -0.58]
**Laparoscopic**	-0.12 [-1.14; 0.89]	Laparoscopic	-1.50 [-2.95; -0.05]
**Open**	-1.98 [-3.42; -0.54]	-1.86 [-3.12; -0.59]	Open

Supplementary Table S23: Assessment of inconsistency for all studies related to outcome quantity of blood transfused.

	Comparison	No.Studies	NMA	Direct	Indirect	Difference	Diff_95CI_lower	Diff_95CI_upper	pValue
1	Laparoscopic:Open	1	-1.85866556272519	-1.5	-3.02	1.52	-1.45914525650088	4.49914525650088	0.317310507862914
2	Laparoscopic:Robotic	1	0.124125444295657	-0.0800000000000001	1.44	-1.52	-4.49914525650088	1.45914525650088	0.317310507862913
3	Robotic:Open	1	-1.98279100702085	-2.94	-1.42	-1.52	-4.49914525650088	1.45914525650088	0.317310507862914

11.ICU stay

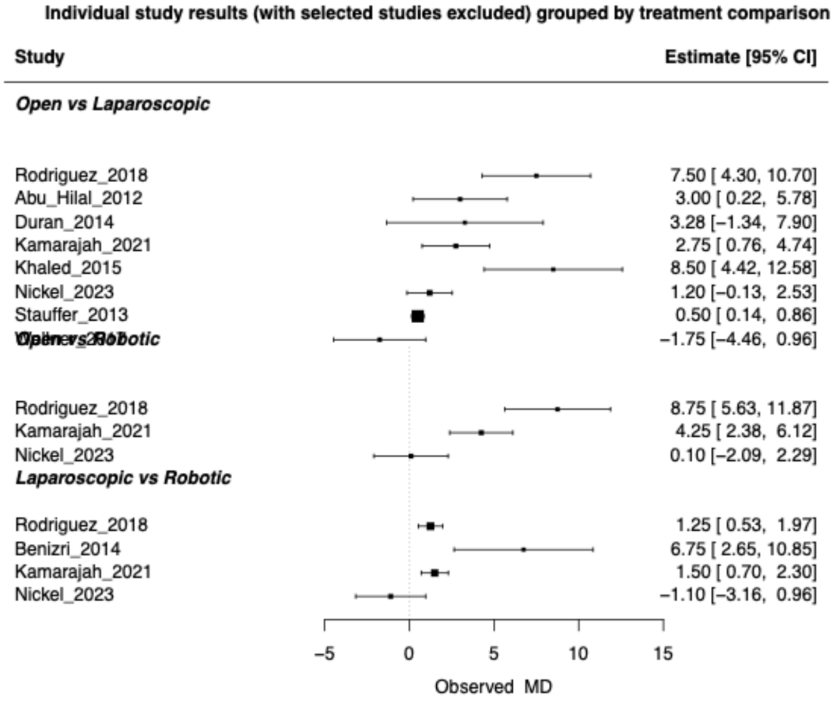

Supplementary Figure S85: Individual study results grouped by treatment comparison for the outcome ICU stay.

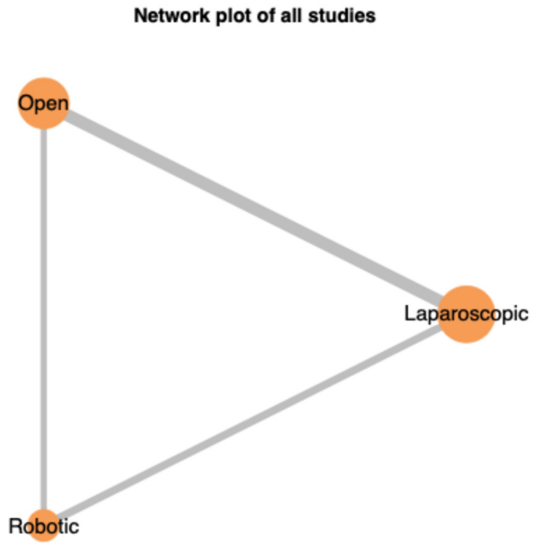

Supplementary Figure S86: Network plot of all studies for outcome ICU stay. The size of the nodes and thickness of edges represent the number of studies that examined a treatment and compared two given treatments respectively.

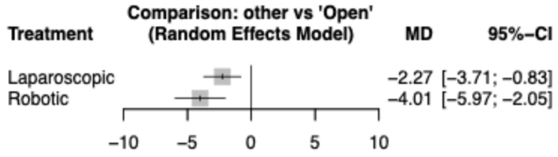

Supplementary Figure S87: Forest plot for outcome ICU stay. The size of the nodes and thickness of edges represent the number of studies that examined a treatment and compared two given treatments respectively.

Supplementary Table S24: Comparison of all treatment pairs related to outcome ICU stay. Treatments are ranked from best to worst along the leading diagonal. Above the leading diagonal are estimates from pairwise meta-analyses, below the leading diagonal are estimates from network meta-analyses. Relative treatment effects in ranked order for all studies

	**Robotic**	**Laparoscopic**	**Open**
**Robotic**	Robotic	-1.48 [-3.35; 0.39]	-3.99 [-6.30; -1.67]
**Laparoscopic**	-1.74 [-3.52; 0.04]	Laparoscopic	-2.50 [-3.95; -1.04]
**Open**	-4.01 [-5.97; -2.05]	-2.27 [-3.71; -0.83]	Open

Supplementary Table S25: Assessment of inconsistency for all studies related to outcome ICU stay.

	**Comparison**	**No.Studies**	**NMA**	**Direct**	**Indirect**	**Difference**	**Diff_95CI_lower**	**Diff_95CI_upper**	**pValue**
**1**	Laparoscopic:Open	8	-2.26988419156948	-2.49779691981468	13.8811562712979	-16.3789531911125	-28.6966639155707	-4.0612424666544	0.009155861363346
**2**	Laparoscopic:Robotic	4	1.7374978919991	1.47524835850292	4.27367574928185	-2.79842739077892	-8.9071835480017	3.31032876644386	0.36925939817614
**3**	Robotic:Open	3	-4.00738208356858	-3.9864497135483	-4.06072048216327	0.0742707686149764	-4.28311480244657	4.43165633967653	0.973349871697191

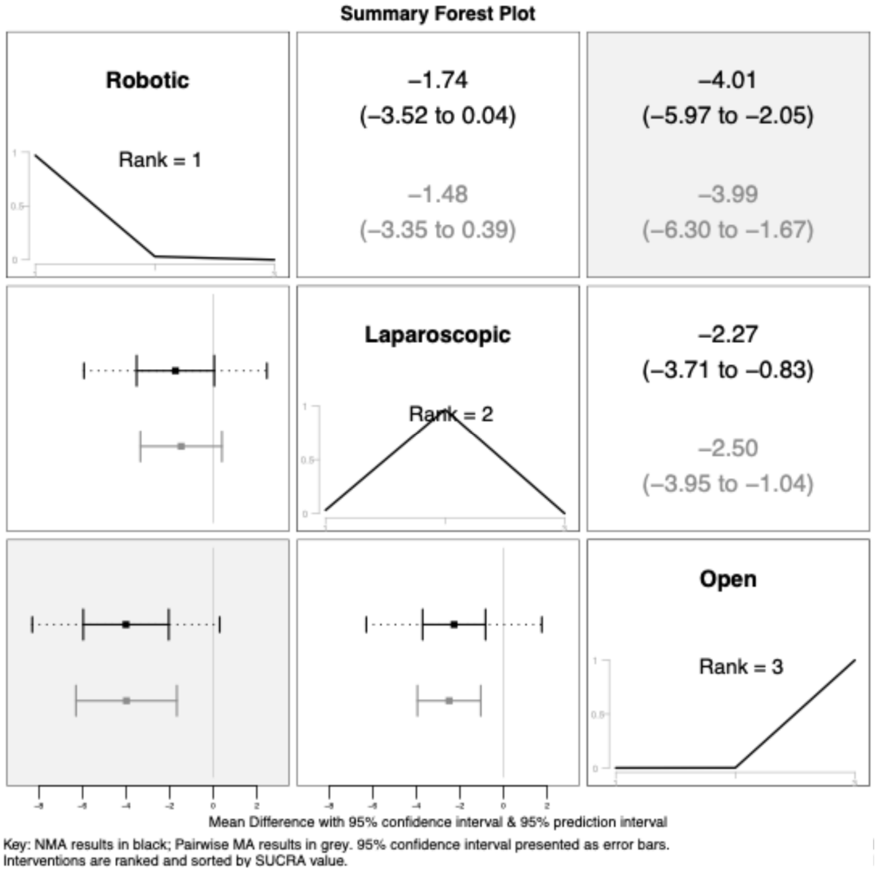

Supplementary Figure S88: Summary Forrest Plot for outcome ICU stay. Ranking of the interventions based on the SUCRA value.

12.Reintervention rate

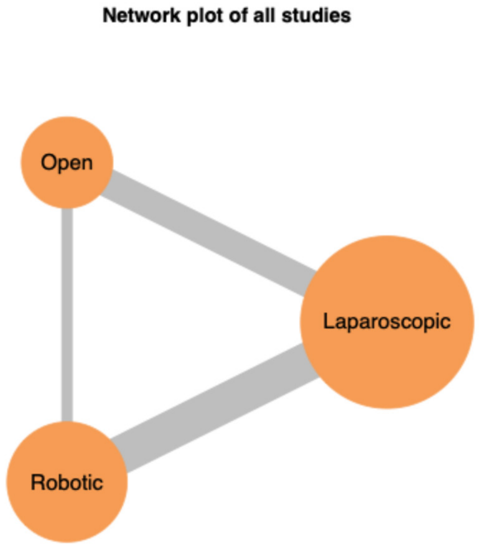

Supplementary Figure S89: Network plot of all studies for outcome reintervention rate. The size of the nodes and thickness of edges represent the number of studies that examined a treatment and compared two given treatments respectively.

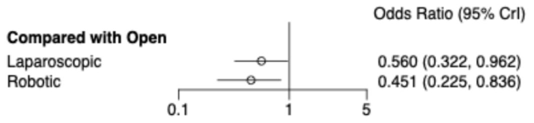

Supplementary Figure S90: Bayesian random effect consistency model forrest plot for outcome reintervention rate.

Supplementary Table S26: Treatment effects for all studies: comparison of all treatment pairs. Outcomes reinterention rate. Bayesian NMA.

	Laparoscopic	Open	Robotic
**Laparoscopic**	Laparoscopic	1.79 (1.04, 3.1)	0.81 (0.47, 1.3)
**Open**	0.56 (0.32, 0.96)	Open	0.45 (0.23, 0.84)
**Robotic**	1.24 (0.77, 2.13)	2.22 (1.2, 4.44)	Robotic

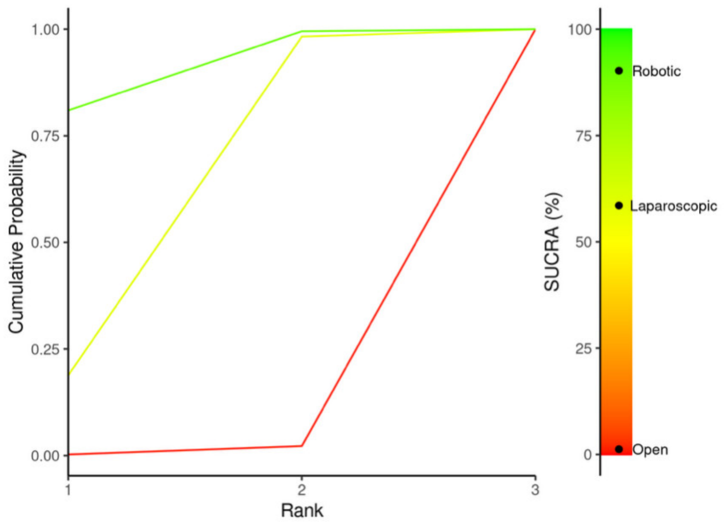

Supplementary Figure S91: Litmus Rank-O-Gram: Outcomes reintervention rate. Higher SUCRA (Surface Under the Cumulative Ranking Curve) values and cumulative ranking curves nearer the top left indicate better performance.

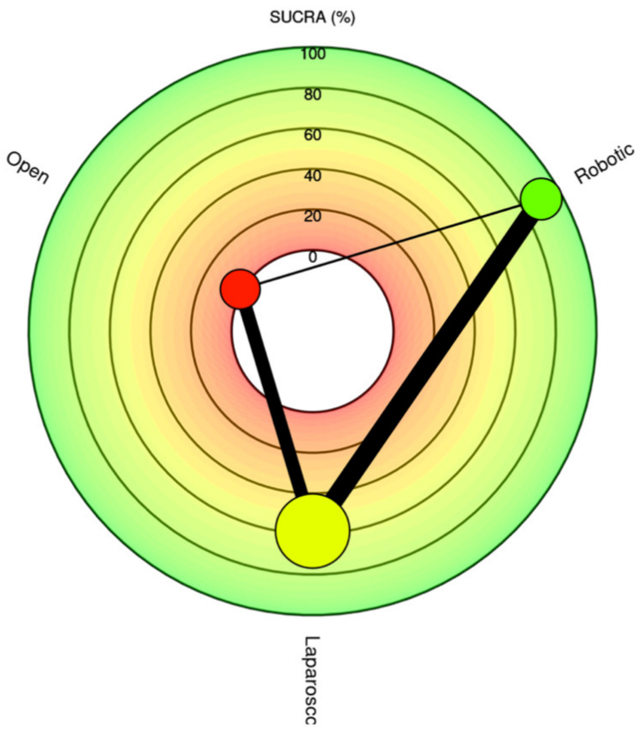

Supplementary Figure S92: Radial SUCRA plot: Outcomes reintervention rate. Higher SUCRA values indicate better treatments; size of nodes represent number of participants and thickness of lines indicate number of trials conducted.

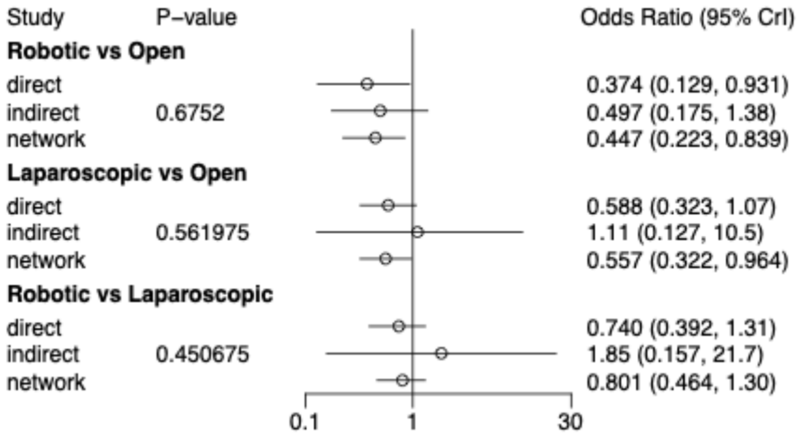

Supplementary Figure S93: Nodesplit model for the outcome reintervention rate.

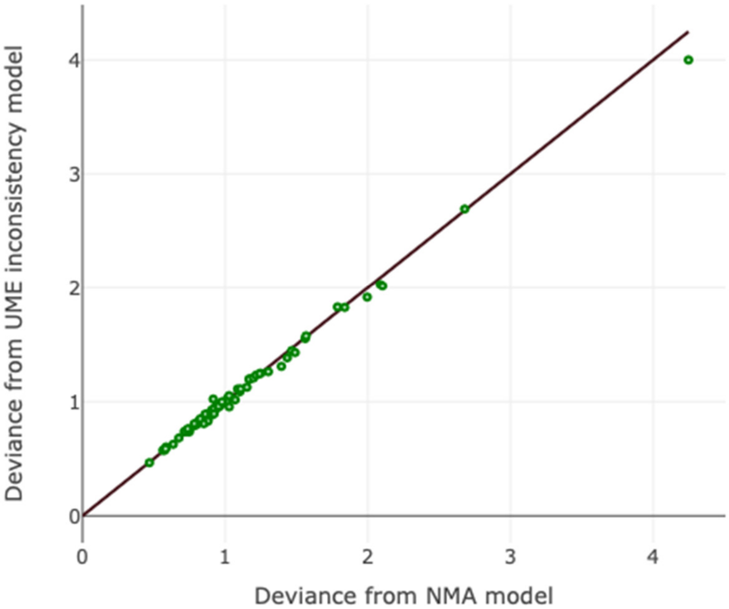

Supplementary Figure S94: Residual deviance from NMA model and UME inconsistency model for all studies.

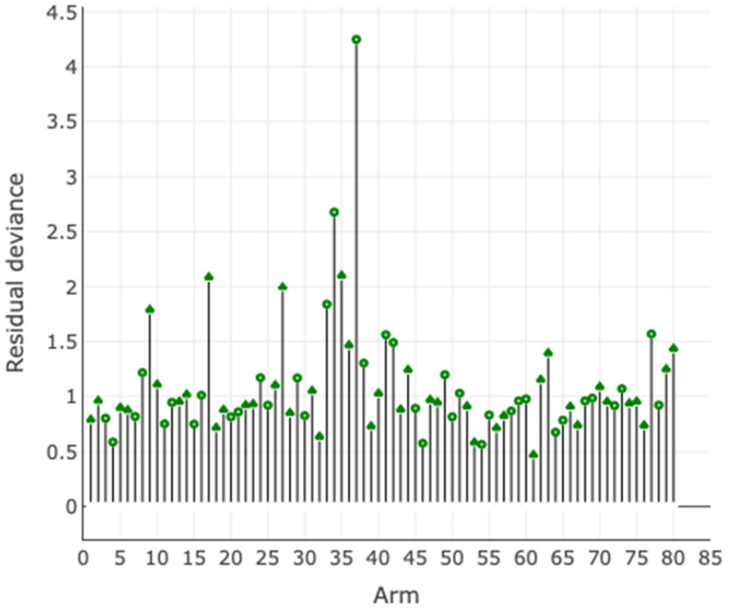

Supplementary Figure S95: Per-arm residual deviance for all studies. This stem plot represents the posterior residual deviance per study arm.

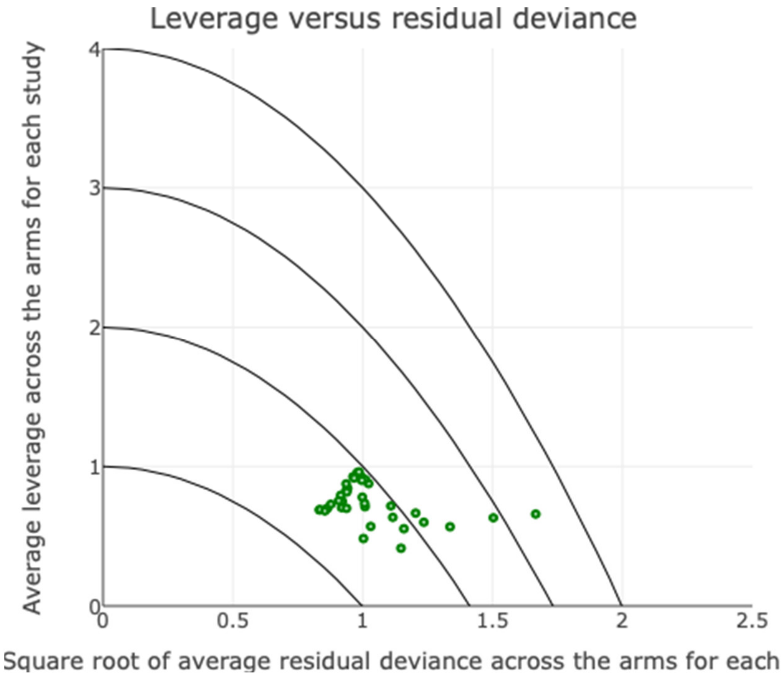

Supplementary Figure S96: Leverage plot for all studies.

13.Hospital stay

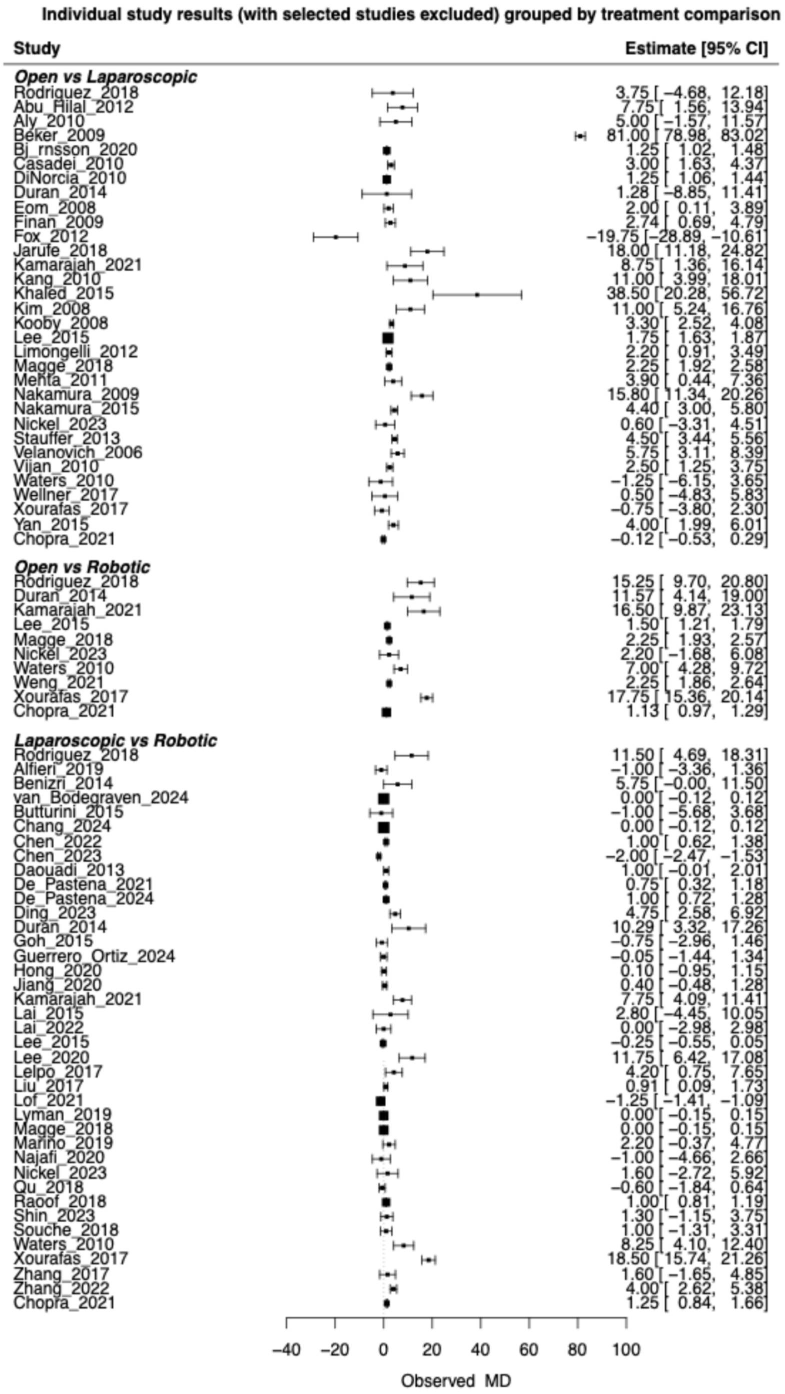

Supplementary Figure S97: Individual study results grouped by treatment comparison for the outcome hospital stay.

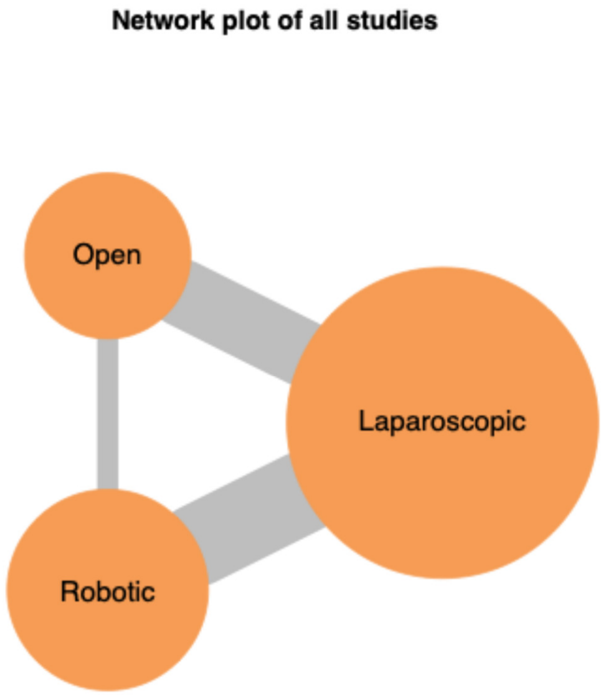

Supplementary Figure S98: Network plot of all studies for outcome hospital stay. The size of the nodes and thickness of edges represent the number of studies that examined a treatment and compared two given treatments respectively.

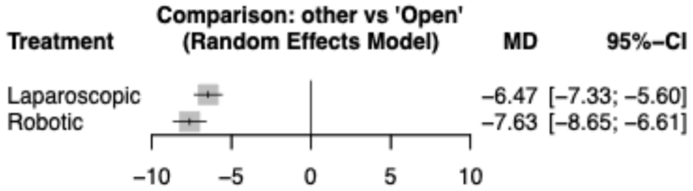

Supplementary Figure S99: Forest plot for outcome hospital stay. Between-study standard deviation: 2.07 , Number of studies: 63 , Number of treatments: 3. All outcomes are versus the reference treatment: Open.

Supplementary Table S27: Comparison of all treatment pairs related to outcome hospital stay. Treatments are ranked from best to worst along the leading diagonal. Above the leading diagonal are estimates from pairwise meta-analyses, below the leading diagonal are estimates from network meta-analyses. Relative treatment effects in ranked order for all studies.

	Robotic	Laparoscopic	Open
**Robotic**	Robotic	-1.67 [-2.42; -0.92]	-5.71 [-7.26; -4.15]
**Laparoscopic**	-1.16 [-1.88; -0.44]	Laparoscopic	-6.43 [-7.35; -5.52]
**Open**	-7.63 [-8.65; -6.61]	-6.47 [-7.33; -5.60]	Open

Supplementary Table S28: Assessment of inconsistency for all studies related to outcome hospital stay.

	Comparison	No.Studies	NMA	Direct	Indirect	Difference	Diff_95CI_lower	Diff_95CI_upper	pValue
**1**	Laparoscopic:Open	32	-6.46515733262787	-6.43297469604174	-6.73315521005415	0.300180514012414	-2.50144720924287	3.1018082372677	0.83366734585659
**2**	Laparoscopic:Robotic	39	1.16333827634672	1.66867995769393	-5.32895898481172	6.99763894250565	4.21241981649106	9.78285806852023	8.46837349508198e-07
**3**	Robotic:Open	10	-7.62849560897459	-5.70567513075511	-9.05911443783396	3.35343930707885	1.29546484318225	5.41141377097545	0.00140446112270975

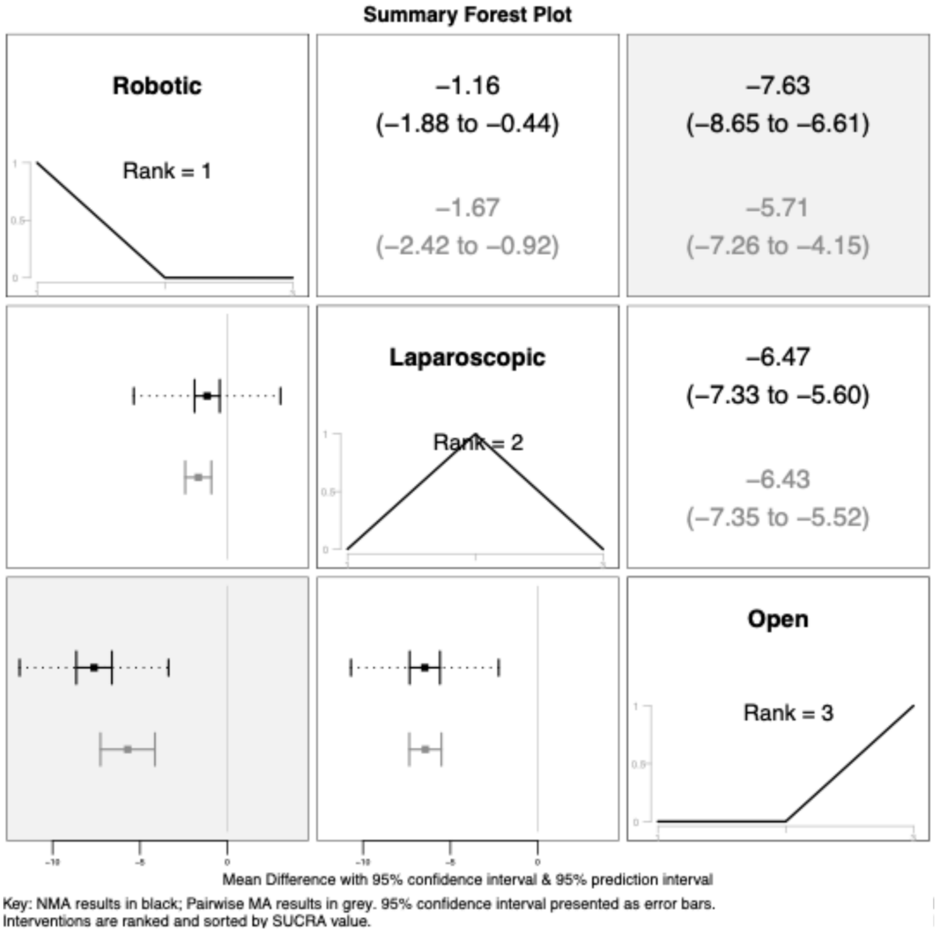

Supplementary Figure S100: Summary Forrest Plot for outcome hospital stay. Ranking of the interventions based on the SUCRA value.

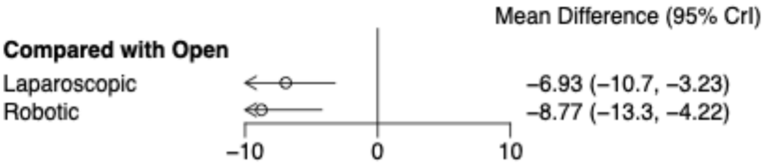

Supplementary Figure S101: Bayesian random effect consistency model forrest plot for outcome hospital stay. Between-study standard deviation: 10.86 . 95% credible interval: 9.14 , 12.96 .

Supplementary Table S29: Treatment effects for all studies: comparison of all treatment pairs. Outcomes hospital stay. Bayesian NMA.

	**Laparoscopic**	**Open**	**Robotic**
**Laparoscopic**	Laparoscopic	6.93 (3.23, 10.67)	-1.83 (-5.17, 1.55)
**Open**	-6.93 (-10.67, -3.23)	Open	-8.77 (-13.34, -4.22)
**Robotic**	1.83 (-1.55, 5.17)	8.77 (4.22, 13.34)	Robotic

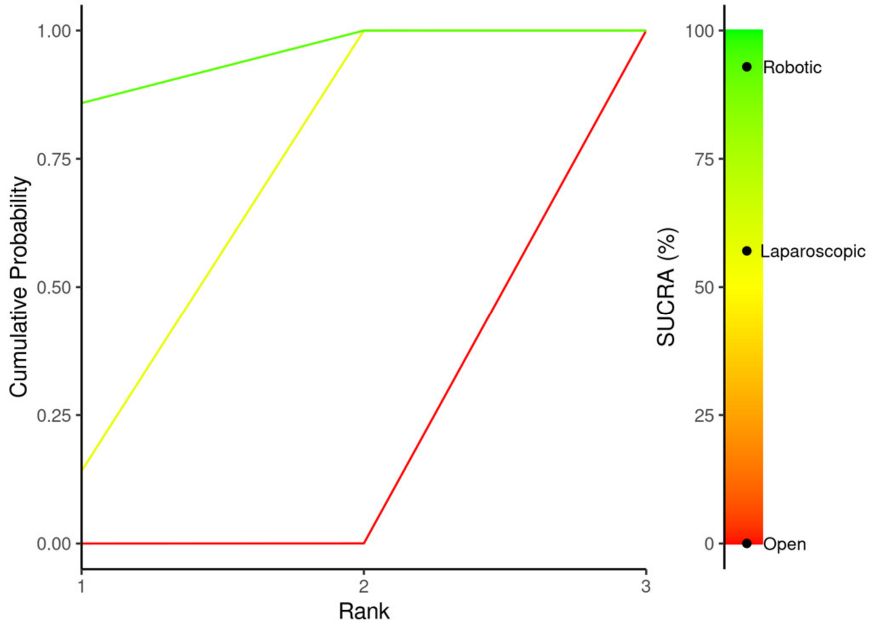

Supplementary Figure S102: Litmus Rank-O-Gram: Outcomes hospital stay. Higher SUCRA (Surface Under the Cumulative Ranking Curve) values and cumulative ranking curves nearer the top left indicate better performance.

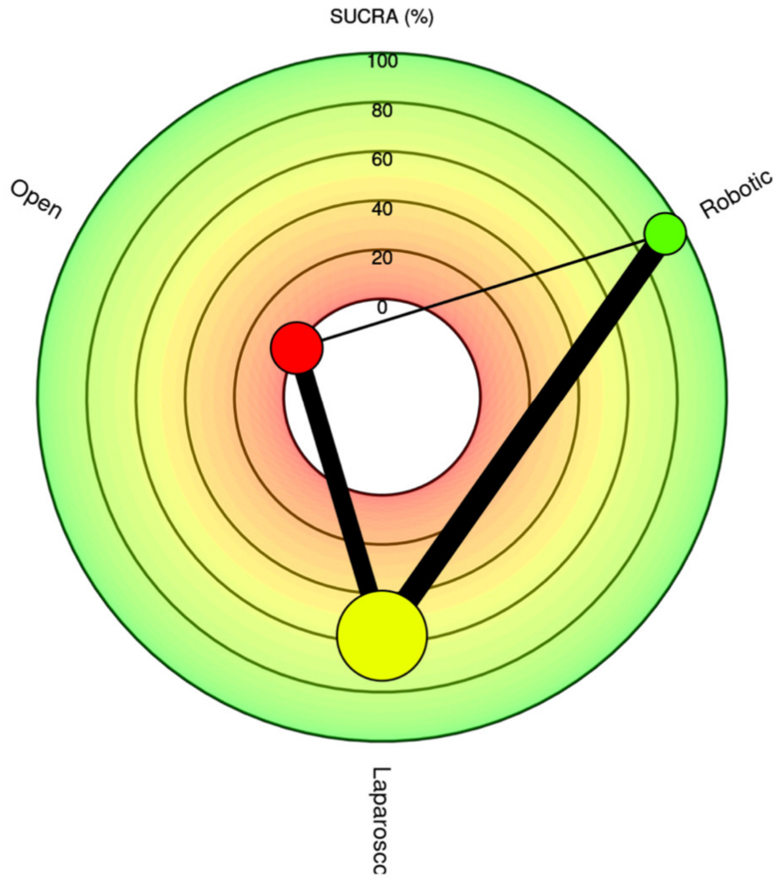

Supplementary Figure S103: Radial SUCRA plot: Outcomes hospital stay. Higher SUCRA values indicate better treatments; size of nodes represent number of participants and thickness of lines indicate number of trials conducted.

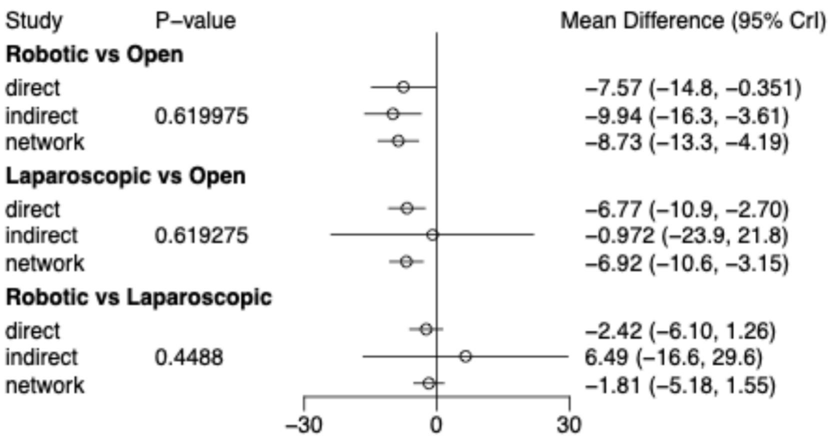

Supplementary Figure S104: Nodesplit model for the outcome hospital stay.

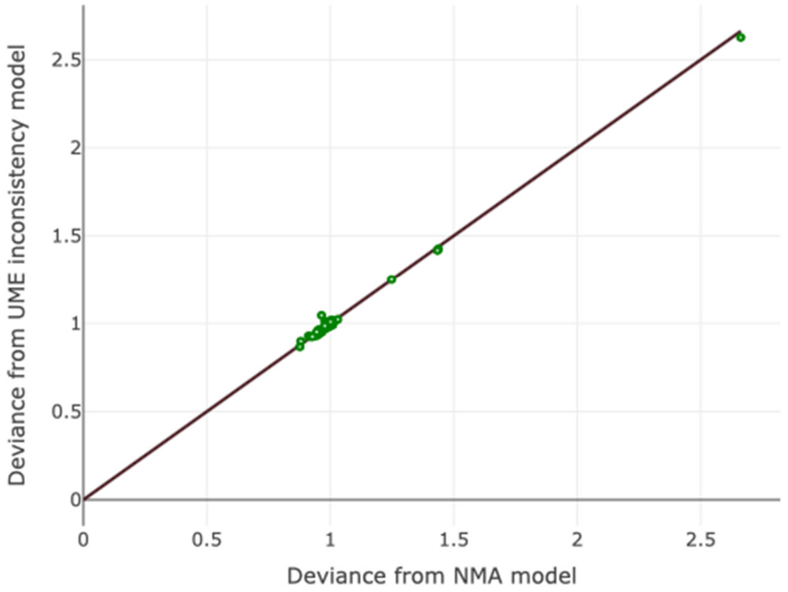

Supplementary Figure S105: Residual deviance from NMA model and UME inconsistency model for all studies.

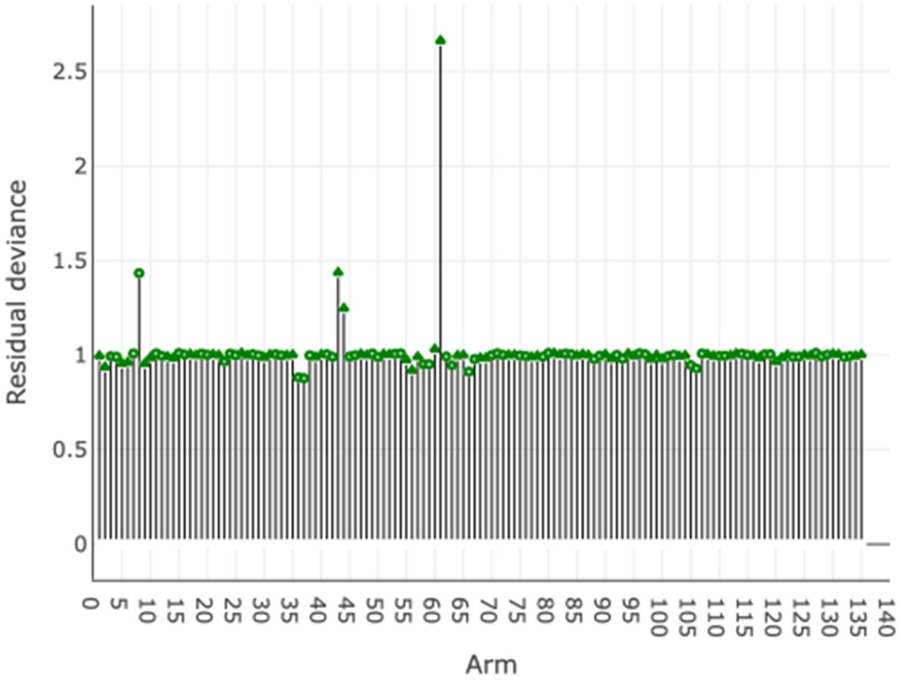

Supplementary Figure S106: Per-arm residual deviance for all studies. This stem plot represents the posterior residual deviance per study arm.

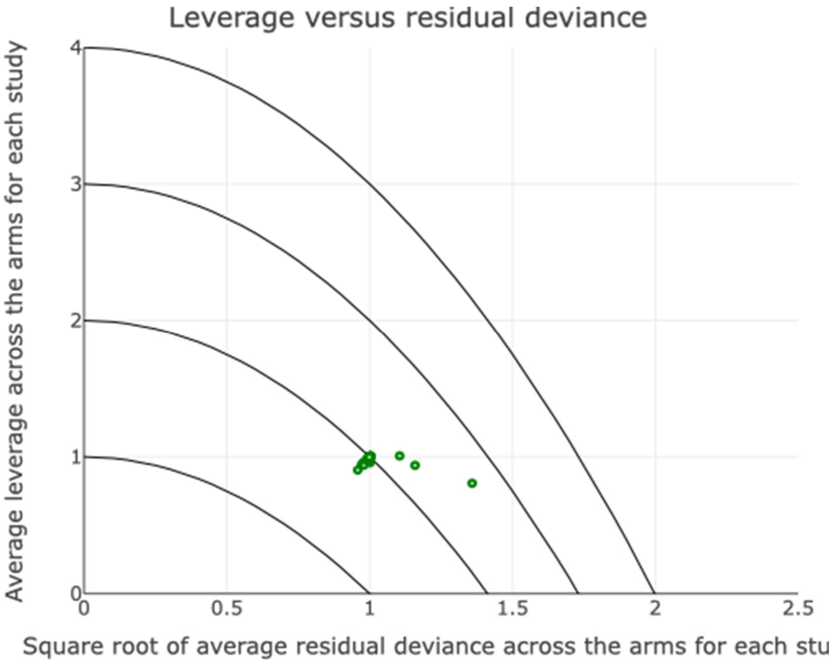

Supplementary Figure S107: Leverage plot for all studies.

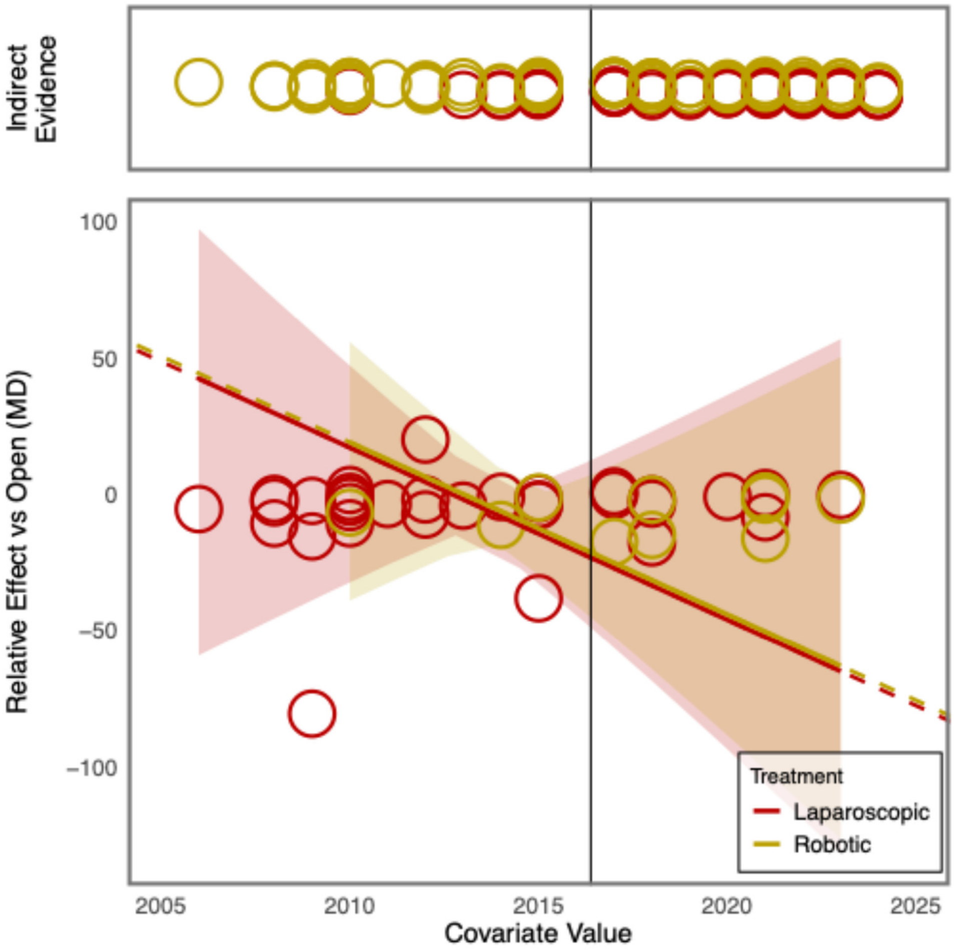

Supplementary Figure S108: Regression plot for the outcome hospital stay having as covariate the year of study publication.

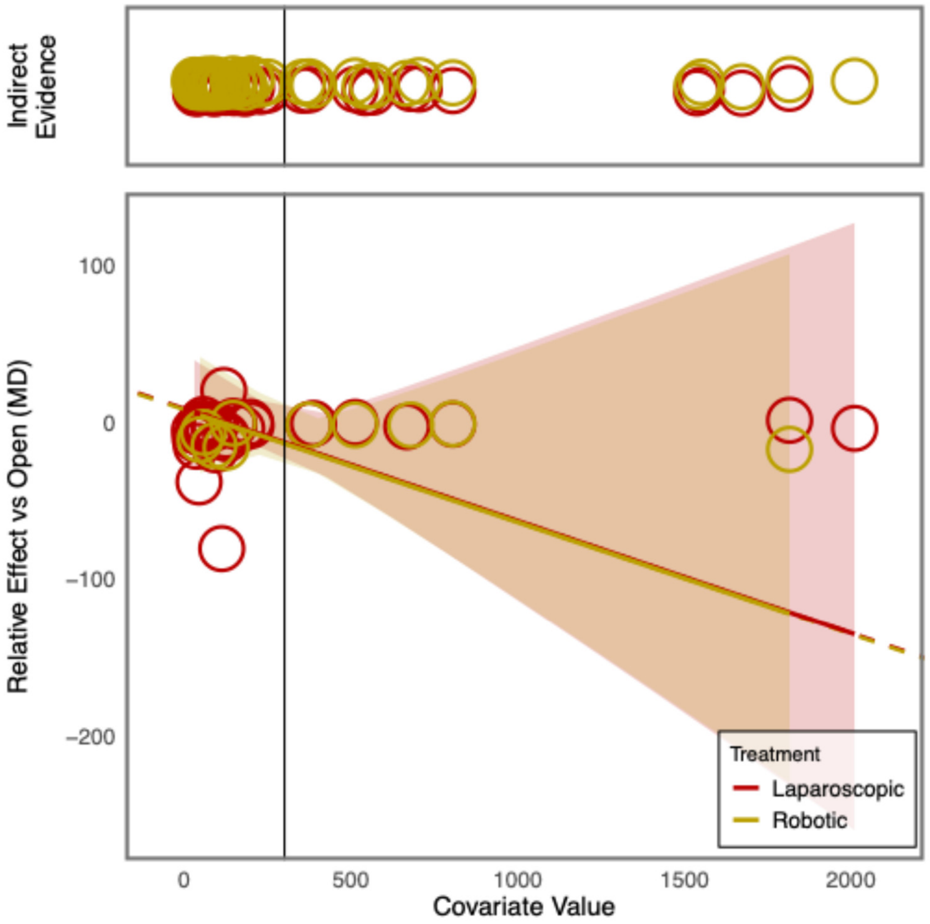

Supplementary Figure S109: Regression plot for the outcome hospital stay having as covariate the number of patients in the inlcuded studies.

14.Readmission rate

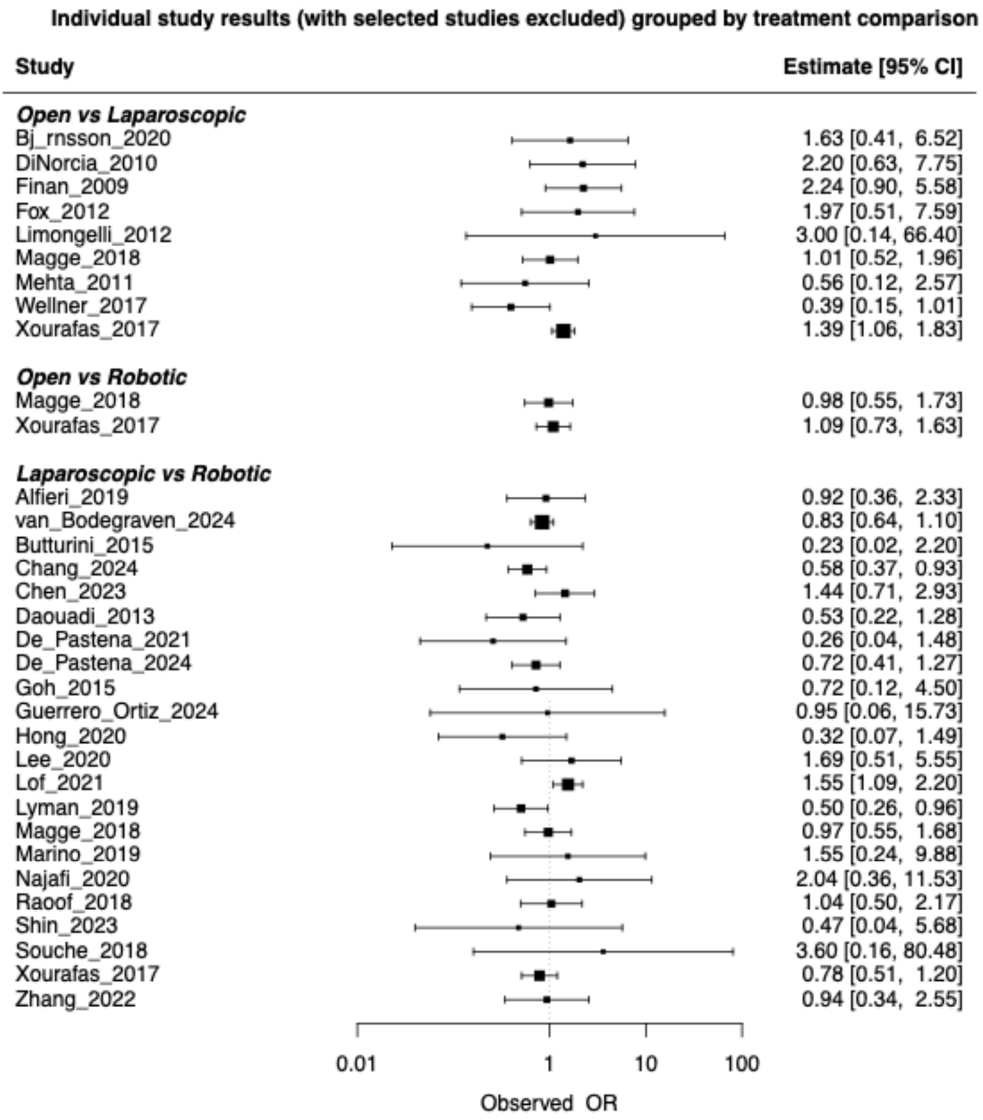

Supplementary Figure S110: Individual study results grouped by treatment comparison for the outcome readmission rate.

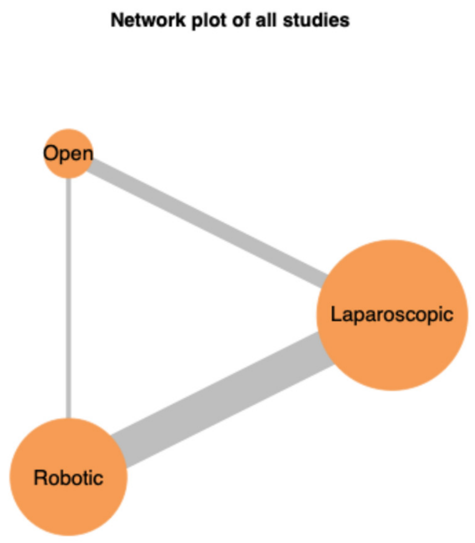

Supplementary Figure S111: Network plot of all studies for outcome readmission rate. The size of the nodes and thickness of edges represent the number of studies that examined a treatment and compared two given treatments respectively.

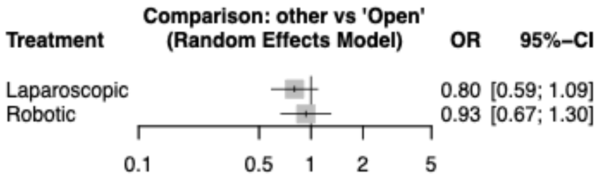

Supplementary Figure S112: Forest plot for outcome readmission rate. The size of the nodes and thickness of edges represent the number of studies that examined a treatment and compared two given treatments respectively. Between-study standard deviation (log-odds scale): 0.22 , Number of studies: 29 , Number of treatments: 3.

Supplementary Table S30: Comparison of all treatment pairs related to outcome readmission rate. Treatments are ranked from best to worst along the leading diagonal. Above the leading diagonal are estimates from pairwise meta-analyses, below the leading diagonal are estimates from network meta-analyses. Relative treatment effects in ranked order for all studies.

	**Laparoscopic**	**Robotic**	**Open**
**Laparoscopic**	Laparoscopic	0.86 [0.71; 1.05]	0.80 [0.58; 1.11]
**Robotic**	0.86 [0.71; 1.04]	Robotic	0.96 [0.61; 1.52]
**Open**	0.80 [0.59; 1.09]	0.93 [0.67; 1.30]	Open

Supplementary Table S31: Assessment of inconsistency for all studies related to outcome readmission rate.

	**Comparison**	**No.Studies**	**NMA**	**Direct**	**Indirect**	**Difference**	**Diff_95CI_lower**	**Diff_95CI_upper**	**pValue**
**1**	Laparoscopic:Open	9	-0.218360537403712	-0.222631724157136	-0.18697809697847	-0.0356536271786658	-0.981578509784171	0.910271255426839	0.941110164471328
**2**	Laparoscopic:Robotic	22	-0.150562599201622	-0.148463744313914	-0.241225097414418	0.0927613531005043	-1.20710767447626	1.39263038067727	0.888764996137389
**3**	Robotic:Open	2	-0.06779793820209	-0.0406989660645829	-0.0976042007983429	0.0569052347337601	-0.60711274694509	0.72092321641261	0.866610303969005

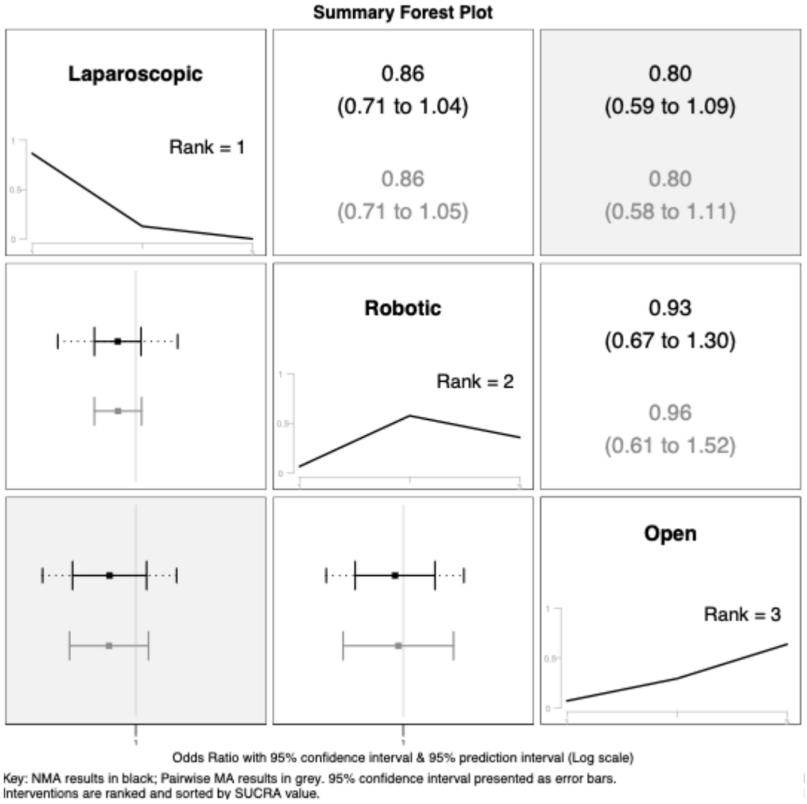

Supplementary Figure S113: Summary Forrest Plot for outcome readmission rate. Ranking of the interventions based on the SUCRA value.

15.In-hospital mortality

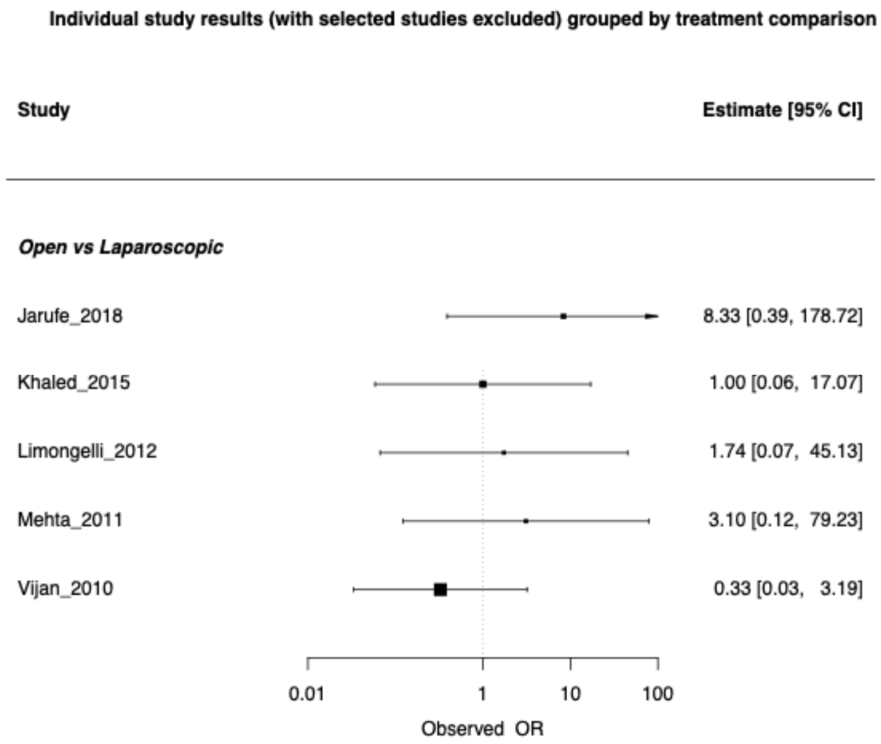

Supplementary Figure S114: Individual study results grouped by treatment comparison for the outcome in-hospital mortality.

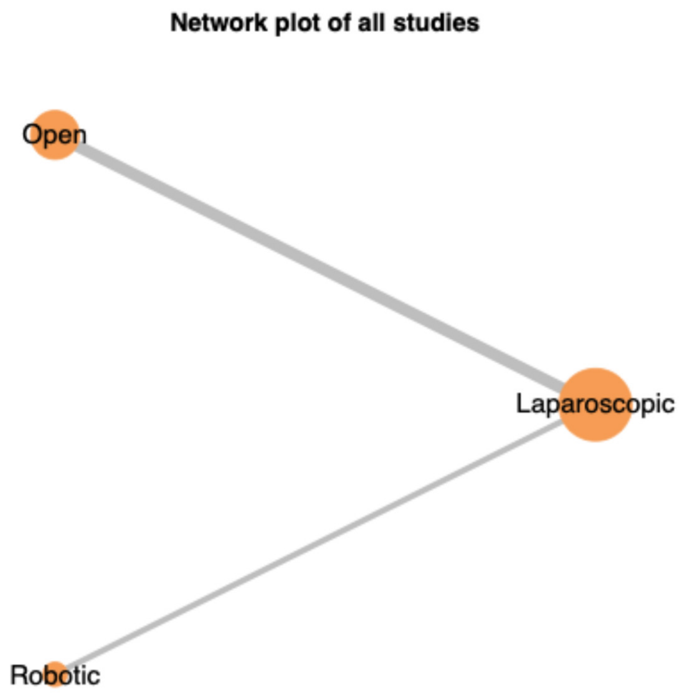

Supplementary Figure S115: Network plot of all studies for outcome in-hospital mortality. The size of the nodes and thickness of edges represent the number of studies that examined a treatment and compared two given treatments respectively.

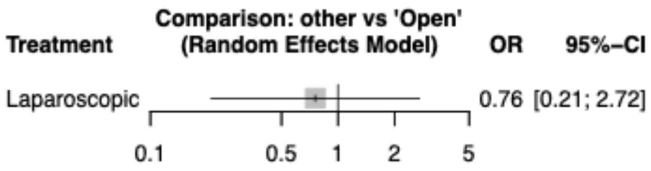

Supplementary Figure S116: Forest plot for outcome in-hospital mortality. The size of the nodes and thickness of edges represent the number of studies that examined a treatment and compared two given treatments respectively.

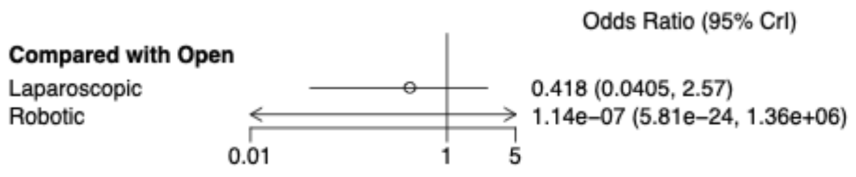

Supplementary Figure S117: Bayesian random effect consistency model forrest plot for outcome in-hospital mortality.

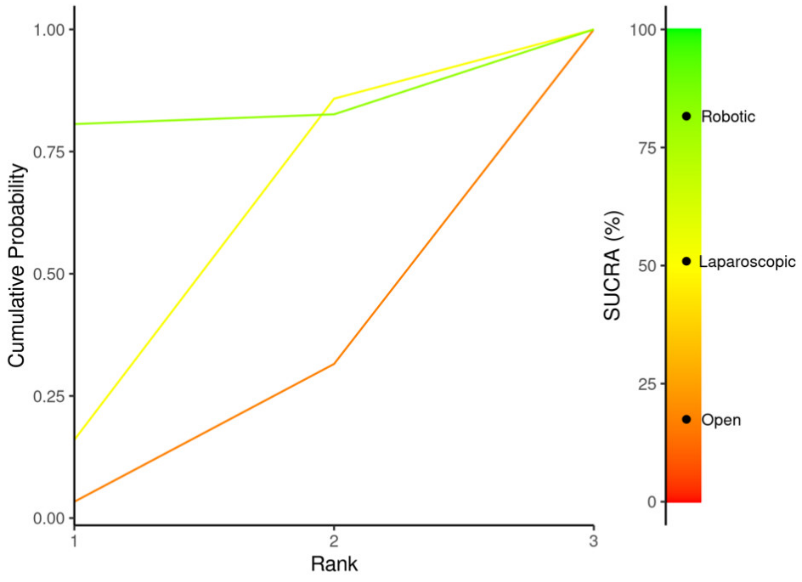

Supplementary Figure S118: Litmus Rank-O-Gram: Outcomes in-hospital mortality. Higher SUCRA (Surface Under the Cumulative Ranking Curve) values and cumulative ranking curves nearer the top left indicate better performance.

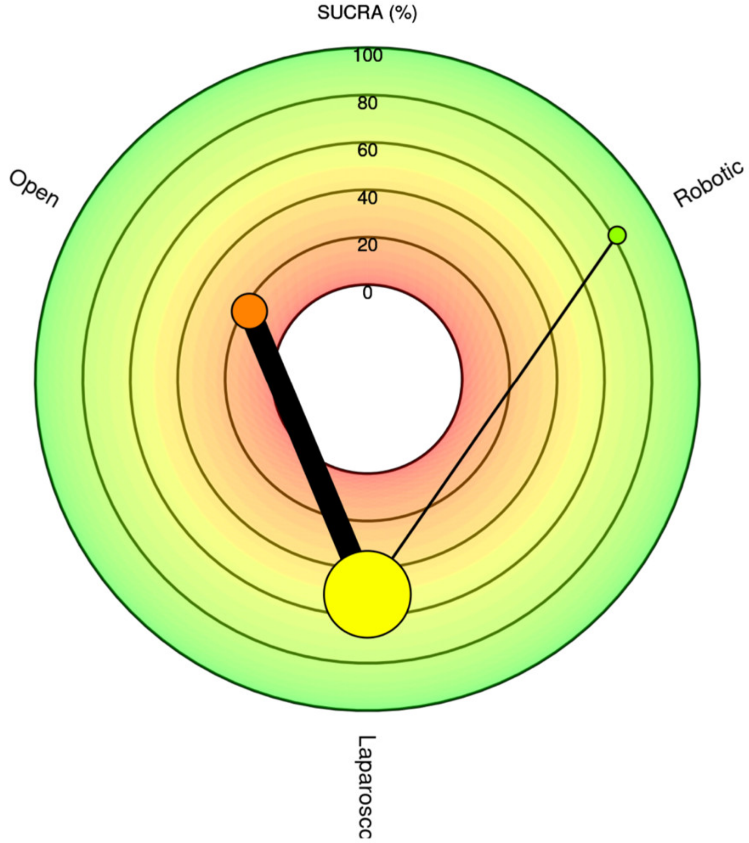

Supplementary Figure S119: Radial SUCRA plot: Outcomes in-hospital mortality. Higher SUCRA values indicate better treatments; size of nodes represent number of participants and thickness of lines indicate number of trials conducted.

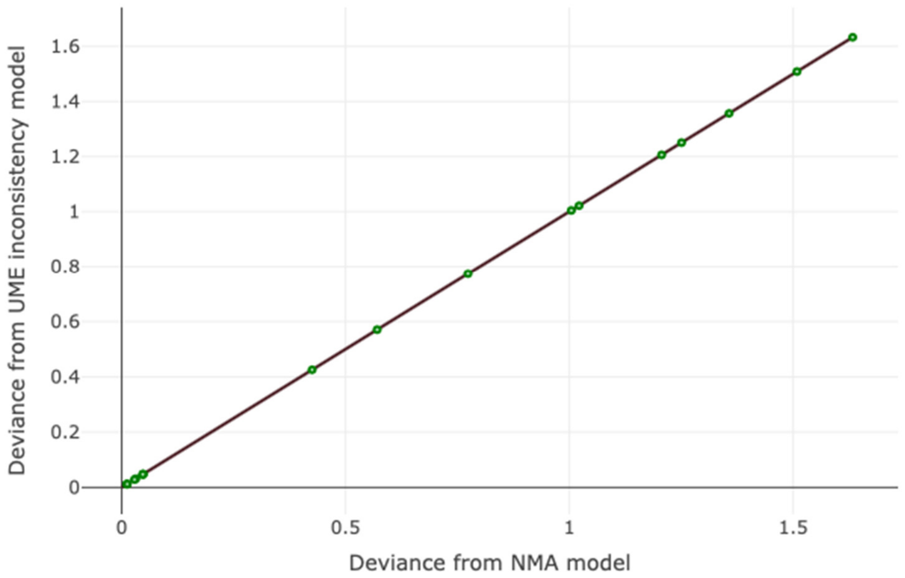

Supplementary Figure S120: Residual deviance from NMA model and UME inconsistency model for all studies.

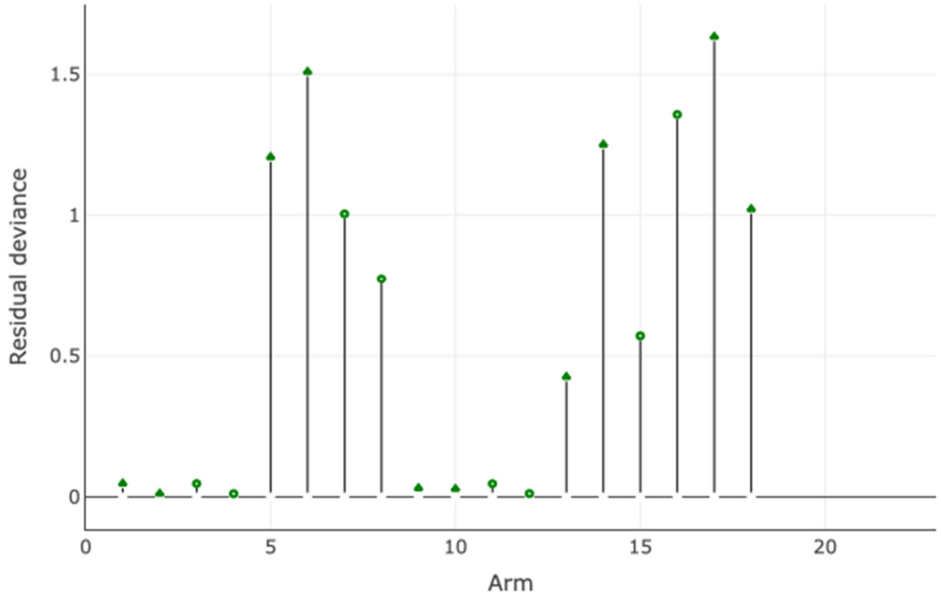

Supplementary Figure S121: Per-arm residual deviance for all studies. This stem plot represents the posterior residual deviance per study arm.

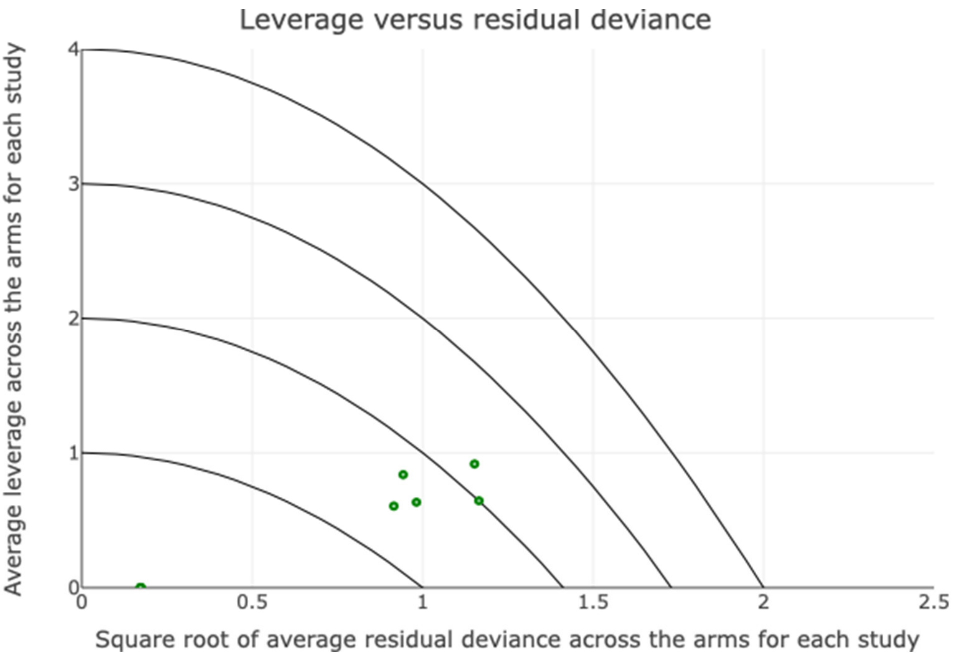

Supplementary Figure S122: Leverage plot for all studies.

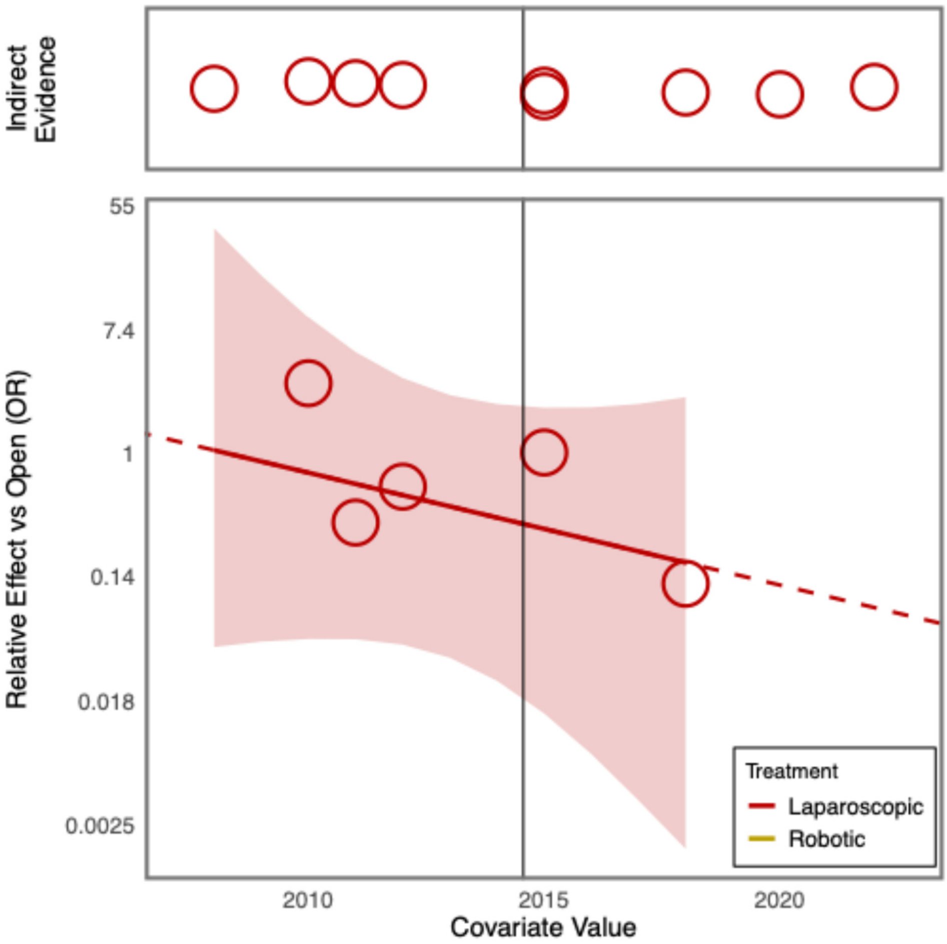

Supplementary Figure S123: Regression plot for the outcome in-hospital mortality having as covariate the year of study publication.

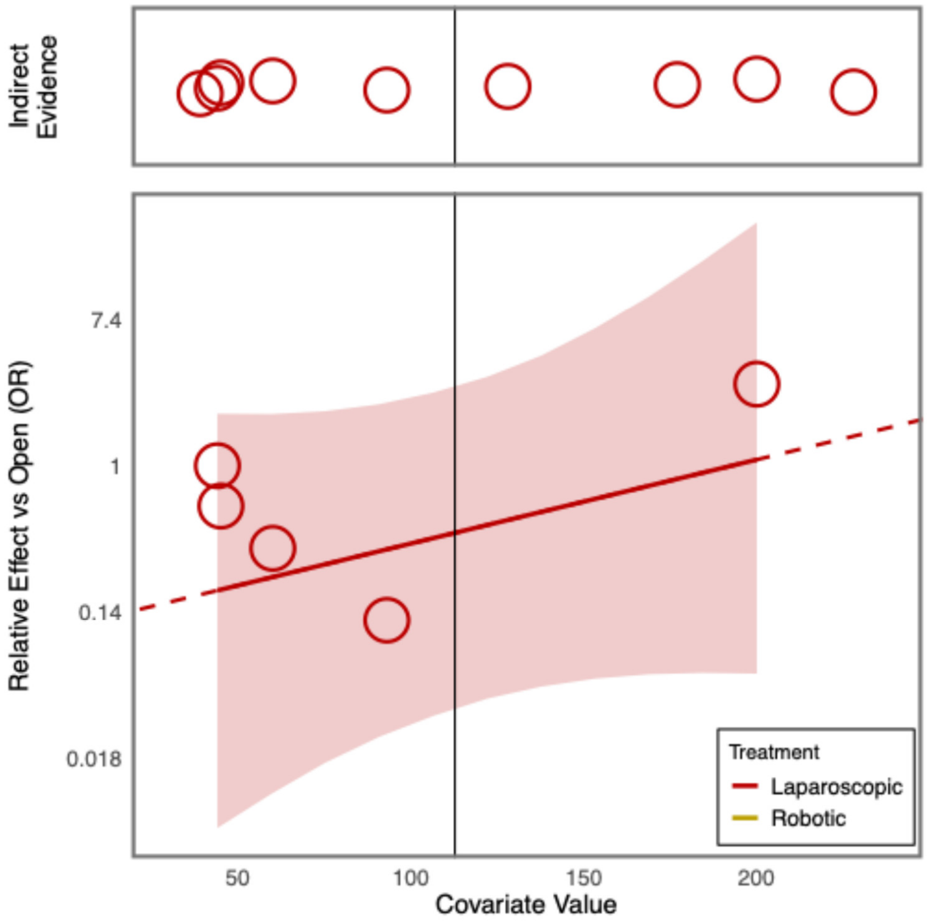

Supplementary Figure S124: Regression plot for the outcome in-hospital mortality having as covariate the number of patients in included studies.

16.30-day mortality

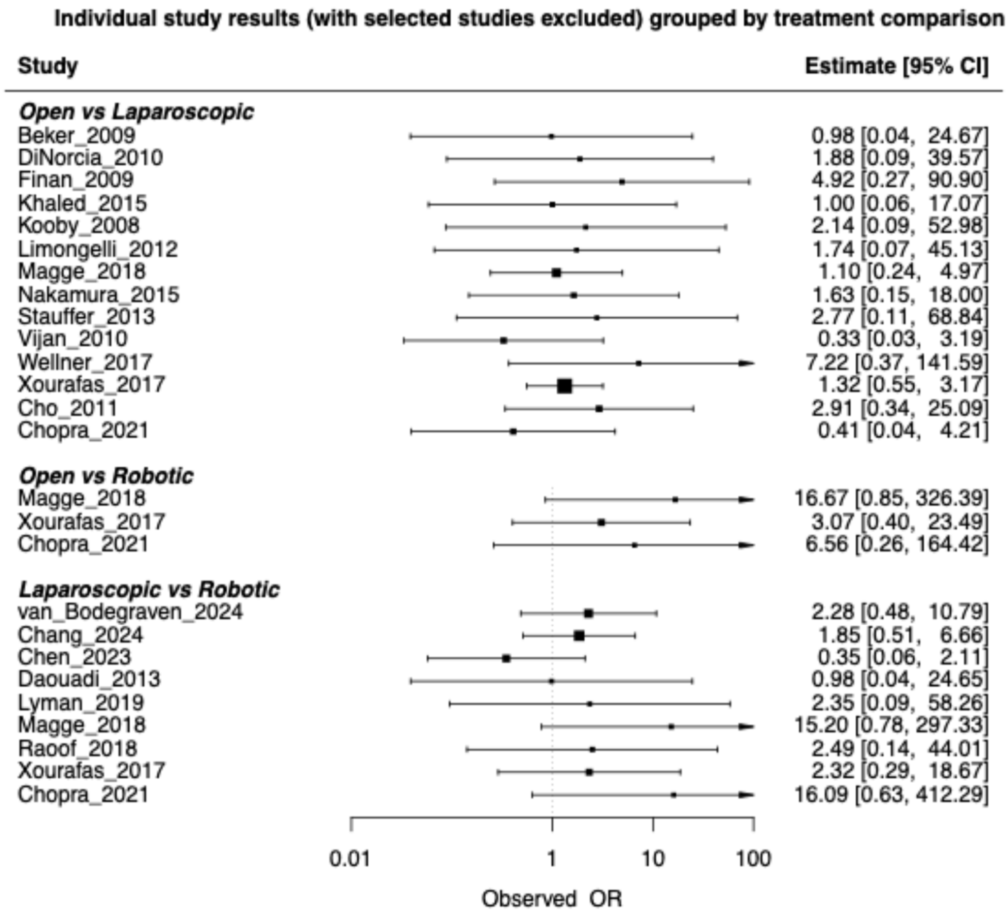

Supplementary Figure S125: Individual study results grouped by treatment comparison for the outcome 30-day mortality.

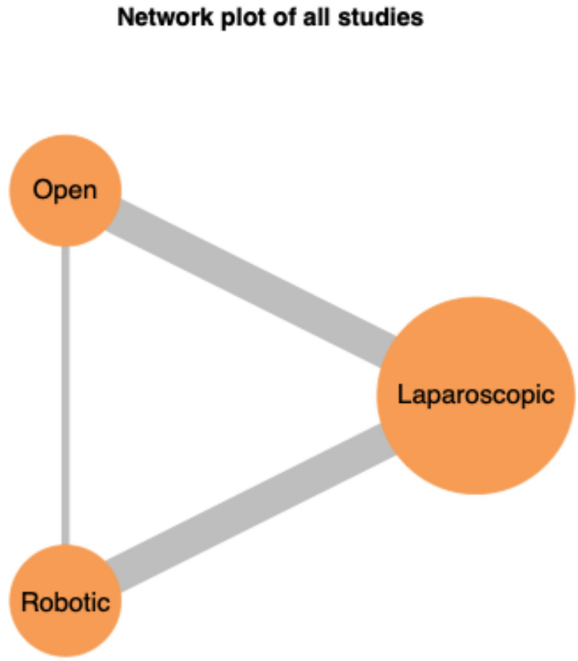

Supplementary Figure S126: Network plot of all studies for outcome 30-day mortality. The size of the nodes and thickness of edges represent the number of studies that examined a treatment and compared two given treatments respectively.

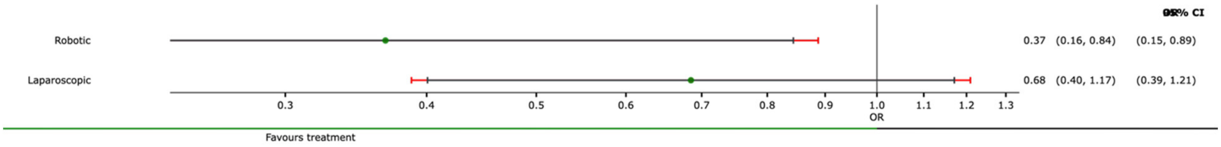

Supplementary Figure S127: Forest plot for outcome 30-day mortality. With red – prediction intervals.

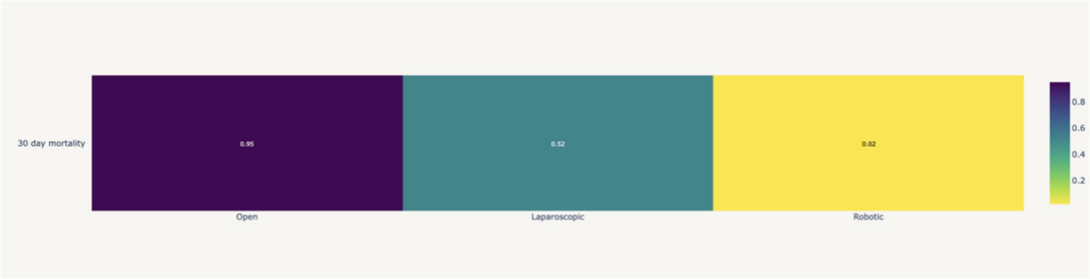

Supplementary Figure S128: P-scores Heatmap for the outcome 30-day mortality.

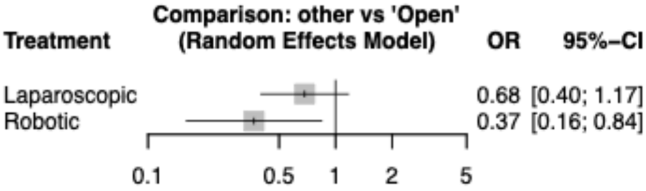

Supplementary Figure S129: Forest plot for outcome 30-day mortality. Between-study standard deviation (log-odds scale): 0 , Number of studies: 20 , Number of treatments: 3. All outcomes are versus the reference treatment: Open.

Supplementary Table S32: Comparison of all treatment pairs related to outcome 30-day mortality. Treatments are ranked from best to worst along the leading diagonal. Above the leading diagonal are estimates from pairwise meta-analyses, below the leading diagonal are estimates from network meta-analyses. Relative treatment effects in ranked order for all studies

	**Robotic**	**Laparoscopic**	**Open**
**Robotic**	Robotic	0.52 [0.26; 1.05]	0.18 [0.04; 0.80]
**Laparoscopic**	0.54 [0.27; 1.07]	Laparoscopic	0.73 [0.42; 1.26]
**Open**	0.37 [0.16; 0.84]	0.68 [0.40; 1.17]	Open

Supplementary Table S33: CINEMA league table.

**Laparoscopic**	**0.702 (0.416, 1.182)**	**1.474 (0.794, 2.735)**
**1.425 (0.846, 2.401)**	Open	2.101 (0.970, 4.552)
**0.678 (0.366, 1.259)**	0.476 (0.220, 1.031)	Robotic

Supplementary Table S34: Assessment of inconsistency for all studies related to outcome 30-day mortality.

	Comparison	No.Studies	NMA	Direct	Indirect	Difference	Diff_95CI_lower	Diff_95CI_upper	pValue
1	Laparoscopic:Open	14	-0.378520290715878	-0.310717066049767	-2.21943298061361	1.90871591456384	-0.98696979565642	4.8044016247841	0.196382438706105
2	Laparoscopic:Robotic	9	0.621631160660939	0.646693519227774	-2.12870619665136	2.77539971587913	-4.51648257074522	10.0672820025035	0.455672443607904
3	Robotic:Open	3	-1.00015145137682	-1.70817230331255	-0.681081261909629	-1.02709104140292	-2.82067903886081	0.766496956054965	0.261707029012972

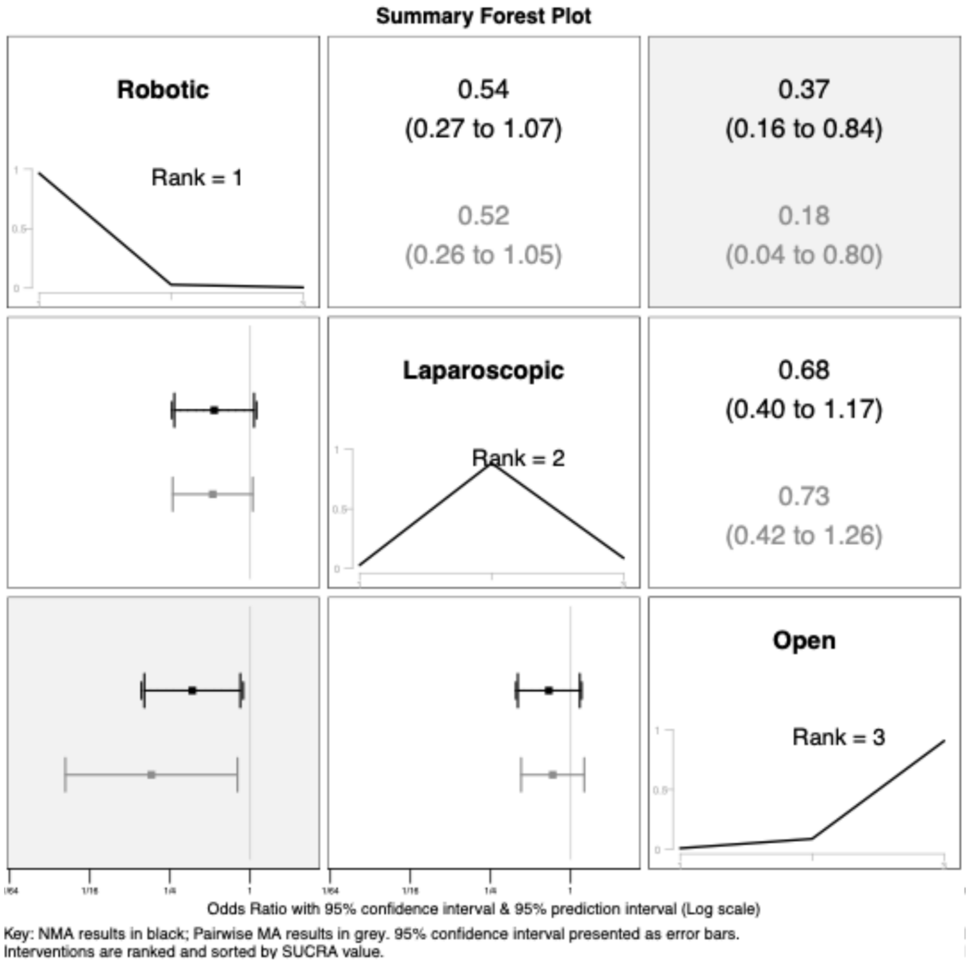

Supplementary Figure S130: Summary Forrest Plot for outcome 30-day mortality. Ranking of the interventions based on the SUCRA value.

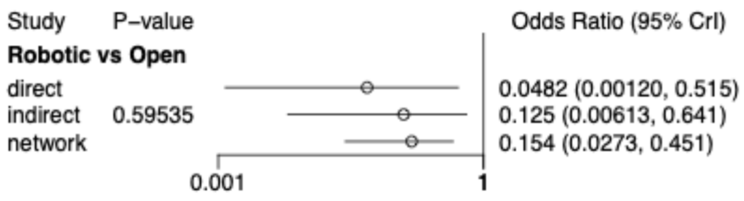

Supplementary Figure S131: Nodesplit model for the outcome 30-day mortality.

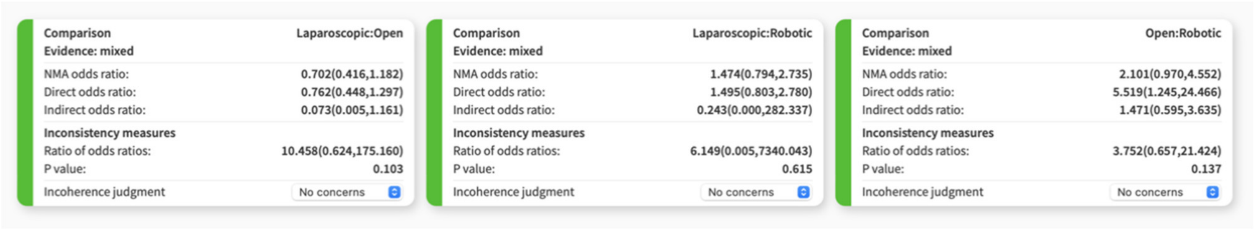

Comparison	Laparoscopic:Open
Evidence: mixed	
NMA odds ratio:	0.702(0.416,1.182)
Direct odds ratio:	0.762(0.448,1.297)
Indirect odds ratio:	0.073(0.005,1.161)
Inconsistency measures	
Ratio of odds ratios:	10.458(0.624,175.160)
P value:	0.103
Incoherence judgment	No concerns

Comparison	Laparoscopic:Robotic
Evidence: mixed	
NMA odds ratio:	1.474(0.794,2.735)
Direct odds ratio:	1.495(0.803,2.780)
Indirect odds ratio:	0.243(0.000,282.337)
Inconsistency measures	
Ratio of odds ratios:	6.149(0.005,7340.043)
P value:	0.615
Incoherence judgment	No concerns

Comparison	Open:Robotic
Evidence: mixed	
NMA odds ratio:	2.101(0.970,4.552)
Direct odds ratio:	5.519(1.245,24.466)
Indirect odds ratio:	1.471(0.595,3.635)
Inconsistency measures	
Ratio of odds ratios:	3.752(0.657,21.424)
P value:	0.137
Incoherence judgment	No concerns

Supplementary Figure S132: evaluation of inconsistency in CINEMA.

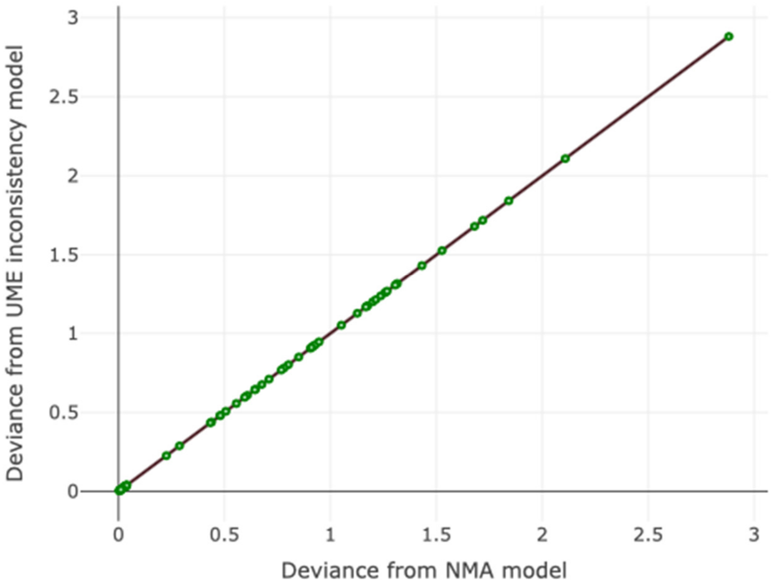

Supplementary Figure S133: Residual deviance from NMA model and UME inconsistency model for all studies.

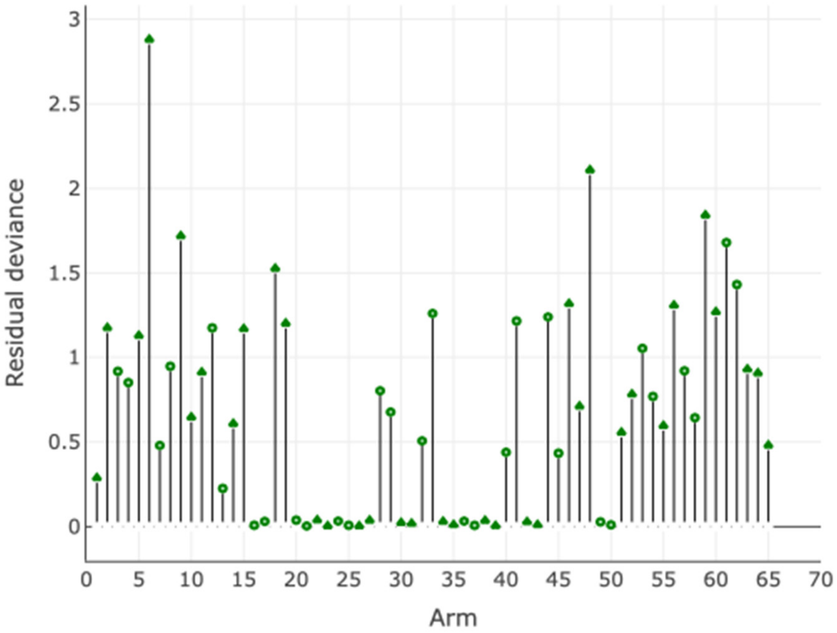

Supplementary Figure S134: Per-arm residual deviance for all studies. This stem plot represents the posterior residual deviance per study arm.

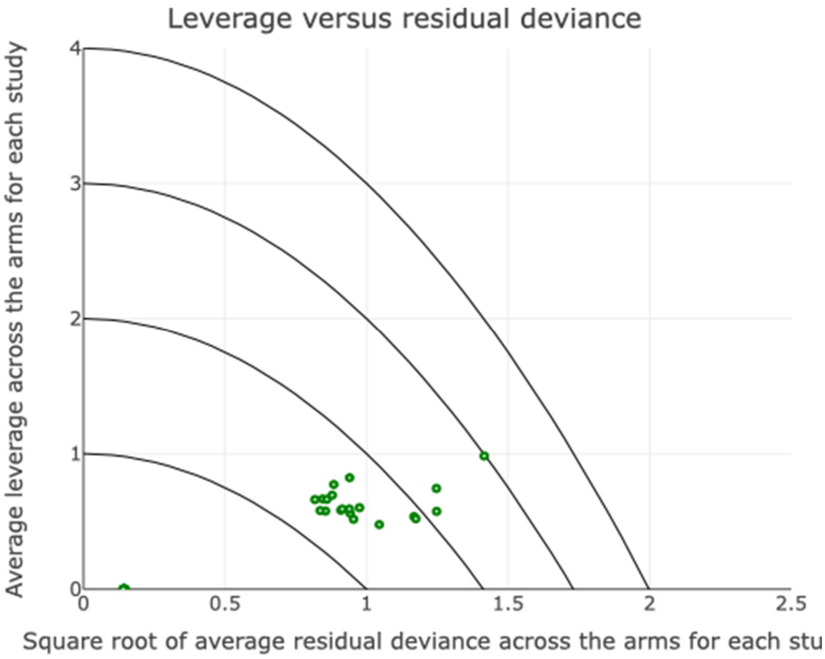

Supplementary Figure S135: Leverage plot for all studies.

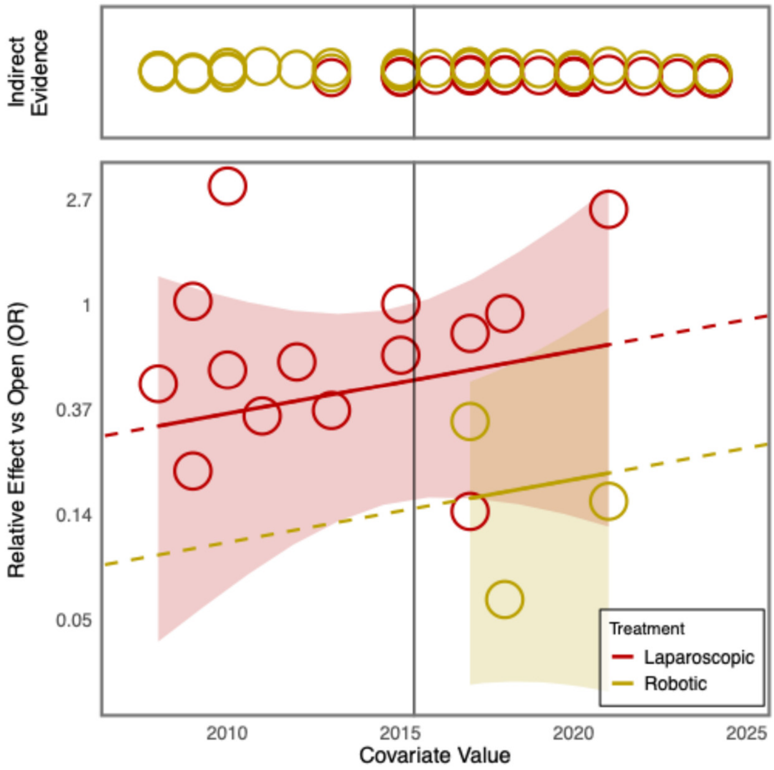

Supplementary Figure S136: Regression plot for the outcome 30-day mortality having as covariate the year of study publication.

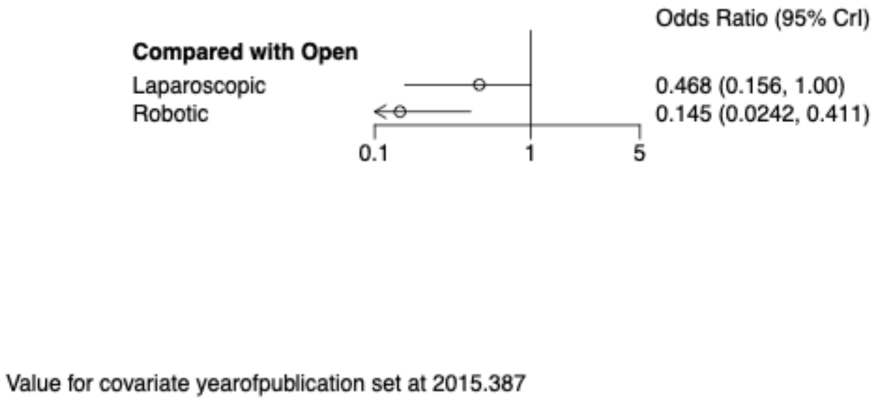

Supplementary Figure S137: Forrest plot of metaregression for the outcome 30-day mortality having as covariate the year of the study publication.

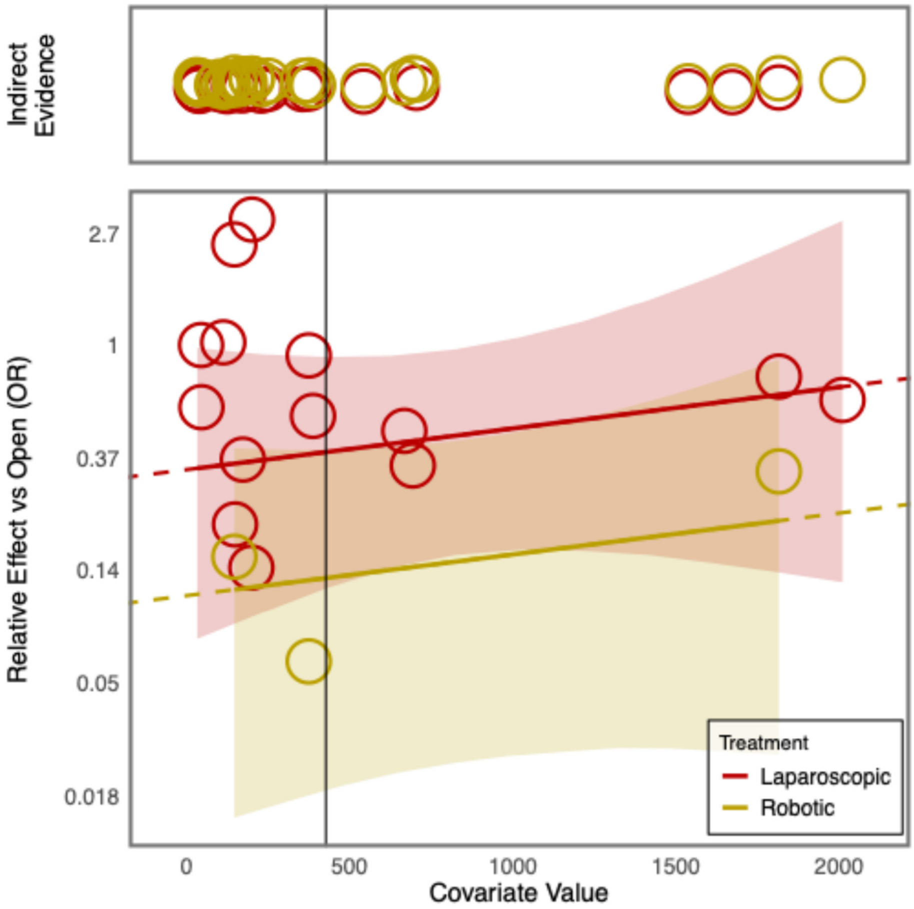

Supplementary Figure S138: Regression plot for the outcome 30-day mortality having as covariate the number of patients in the included studies.

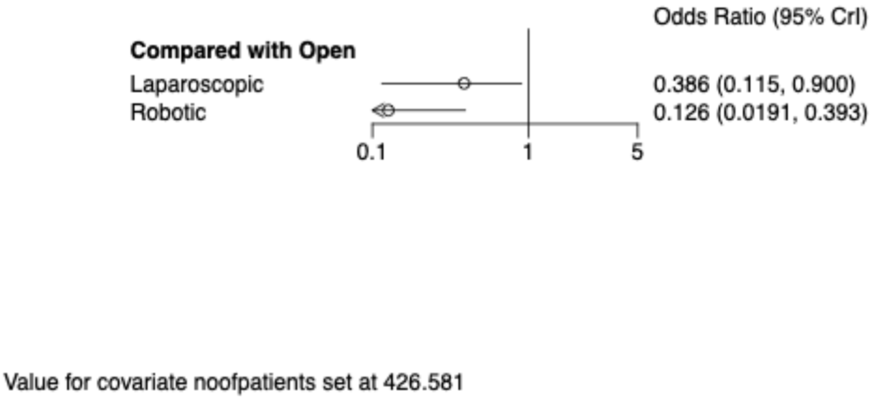

Supplementary Figure S139: Forrest plot of metaregression for the outcome 30-day mortality having as covariate the number of patiemts in theincluded studies.

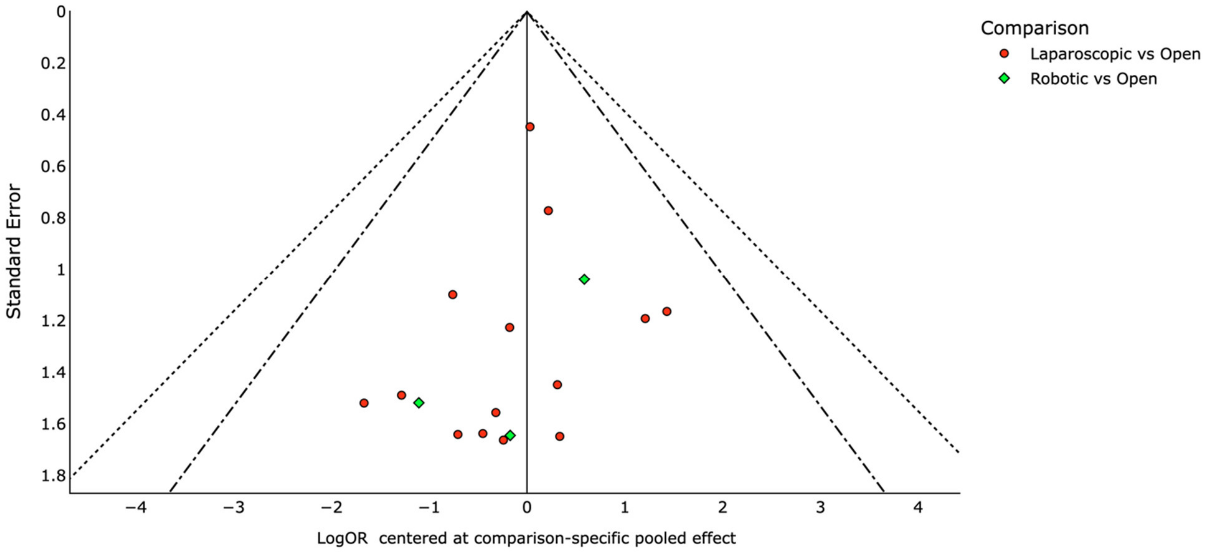

Supplementary Figure S140: Publication bias evaluation for the outcomes 30-day mortality.

17.90-day major complications

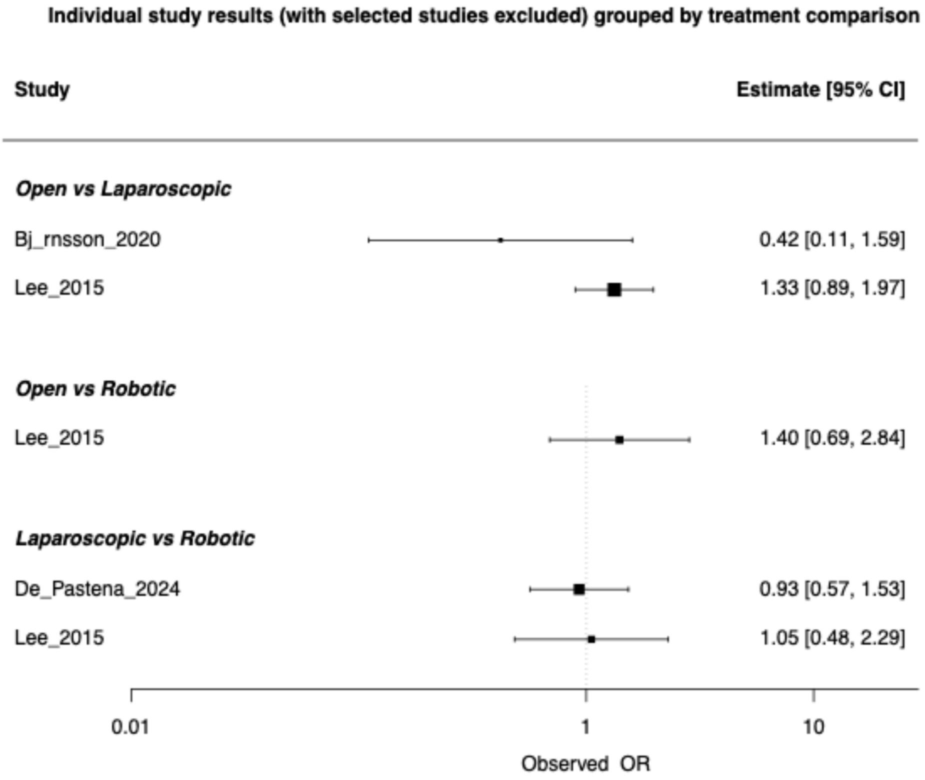

Supplementary Figure S141: Individual study results grouped by treatment comparison for the outcome 90-day major complications.

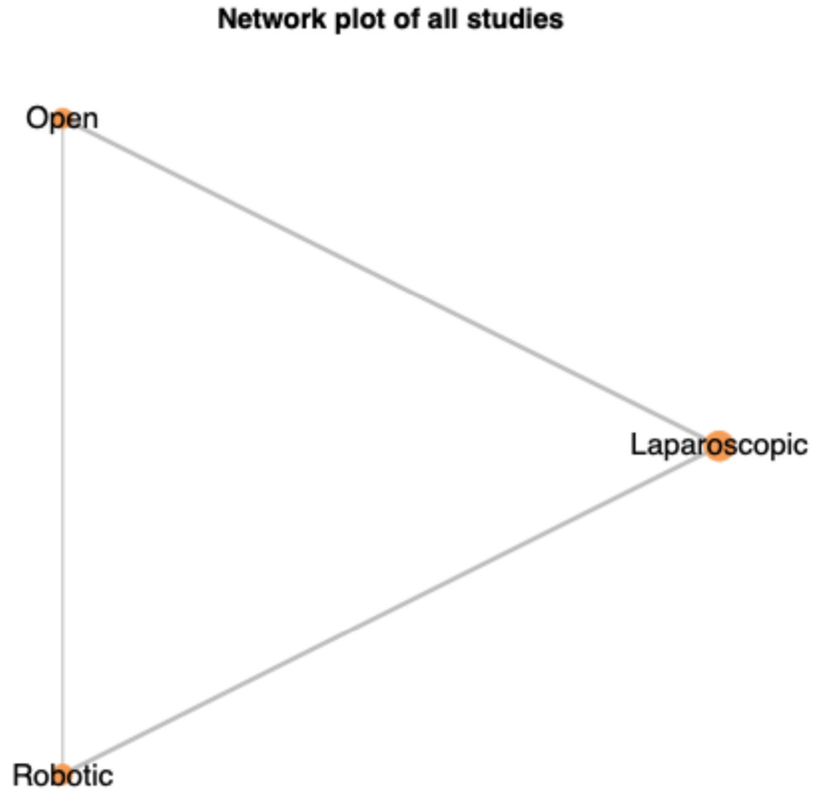

Supplementary Figure S142: Network plot of all studies for outcome 90-day major complications. The size of the nodes and thickness of edges represent the number of studies that examined a treatment and compared two given treatments respectively.

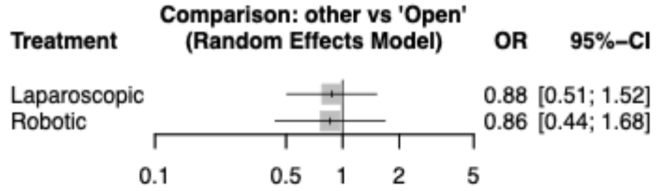

Supplementary Figure S143: Forest plot for outcome 90-day major complications. Between-study standard deviation (log-odds scale): 0.25 , Number of studies: 3 , Number of treatments: 3. All outcomes are versus the reference treatment: Open

Supplementary Table S35: Comparison of all treatment pairs related to outcome 90-day major complication. Treatments are ranked from best to worst along the leading diagonal. Above the leading diagonal are estimates from pairwise meta-analyses, below the leading diagonal are estimates from network meta-analyses. Relative treatment effects in ranked order for all studies

	Robotic	Laparoscopic	Open
**Robotic**	Robotic	1.03 [0.59; 1.80]	0.71 [0.30; 1.69]
**Laparoscopic**	0.98 [0.56; 1.71]	Laparoscopic	0.91 [0.51; 1.63]
**Open**	0.86 [0.44; 1.68]	0.88 [0.51; 1.52]	Open

Supplementary Table S36: Assessment of inconsistency for all studies related to outcome 90-day major complications.

	Comparison	No.Studies	NMA	Direct	Indirect	Difference	Diff_95CI_lower	Diff_95CI_upper	pValue
**1**	Laparoscopic:Open	2	-0.132279178331727	-0.0928277945166356	-0.480843755083479	0.388015960566844	-1.43013923065722	2.20617115179091	0.675742670072934
**2**	Laparoscopic:Robotic	2	0.0198914668006144	-0.0263735095479554	3.90314518729222	-3.92951869684018	-9.08671399413491	1.22767660045456	0.135334559018435
**3**	Robotic:Open	1	-0.152170645132341	-0.336347244433062	0.131663944262061	-0.468011188695123	-1.84425599545742	0.908233618067173	0.50508324817545

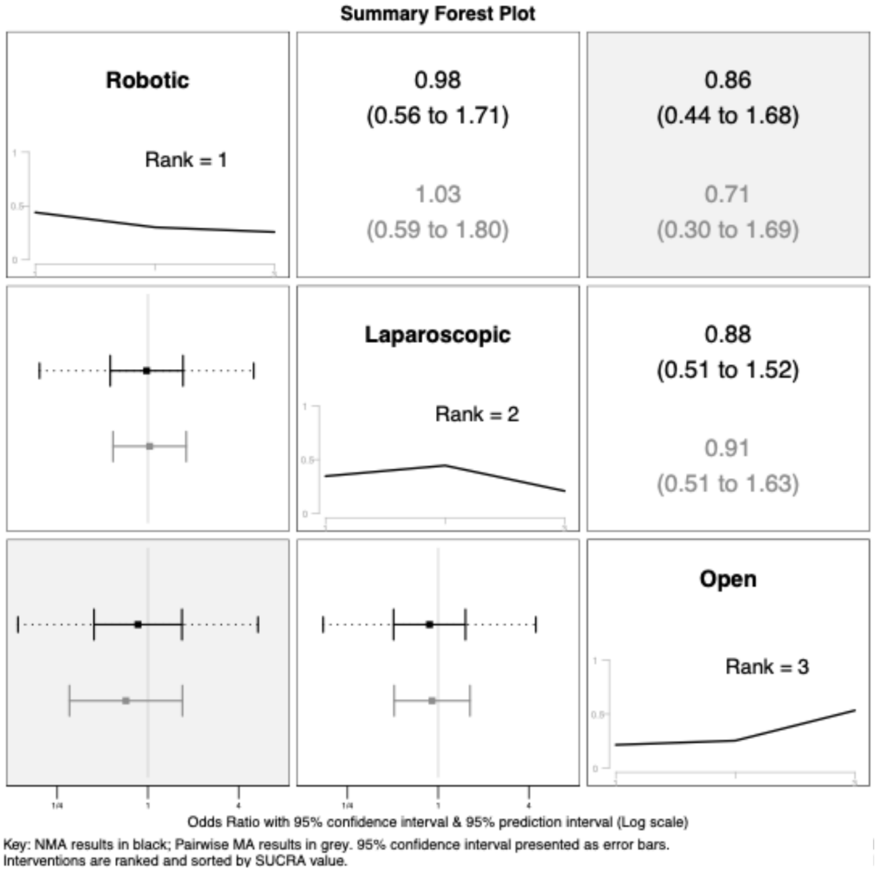

Supplementary Figure S144: Summary Forrest Plot for outcome 90-day major complications. Ranking of the interventions based on the SUCRA value.

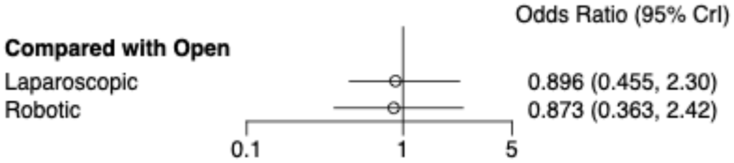

Supplementary Figure S145: Bayesian random effect consistency model forrest plot for outcome 90-day major complications.
Between-study standard deviation (log-odds scale): 0.37 . 95% credible interval: 0.02 , 0.78 .

Supplementary Table S37: Treatment effects for all studies: comparison of all treatment pairs. Outcomes 90-day major complications. Bayesian NMA.

	Laparoscopic	Open	Robotic
**Laparoscopic**	Laparoscopic	1.12 (0.43, 2.2)	0.97 (0.42, 1.99)
**Open**	0.9 (0.45, 2.3)	Open	0.87 (0.36, 2.42)
**Robotic**	1.03 (0.5, 2.36)	1.15 (0.41, 2.75)	Robotic

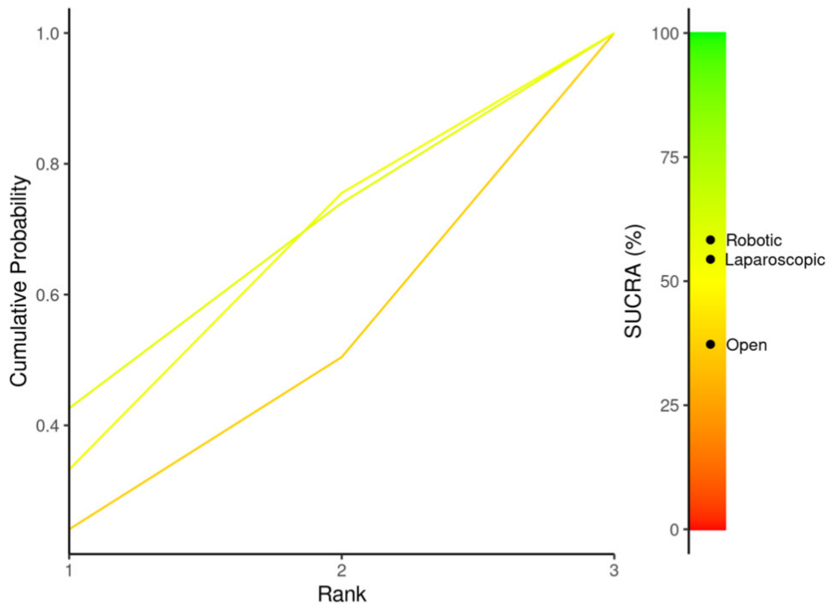

Supplementary Figure S146: Litmus Rank-O-Gram: Outcomes 90-day major complications. Higher SUCRA (Surface Under the Cumulative Ranking Curve) values and cumulative ranking curves nearer the top left indicate better performance.

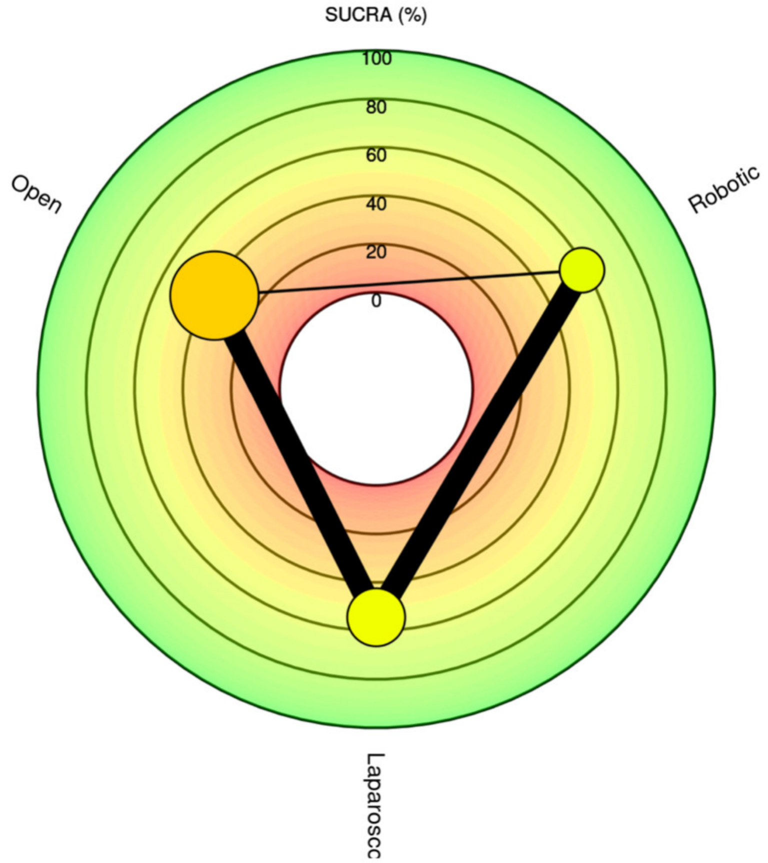

Supplementary Figure S147: Radial SUCRA plot: Outcomes 90-day major complications. Higher SUCRA values indicate better treatments; size of nodes represent number of participants and thickness of lines indicate number of trials conducted.

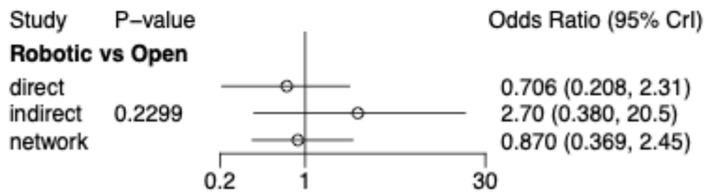

Supplementary Figure S148: Nodesplit model for the outcome 90-day major complications.

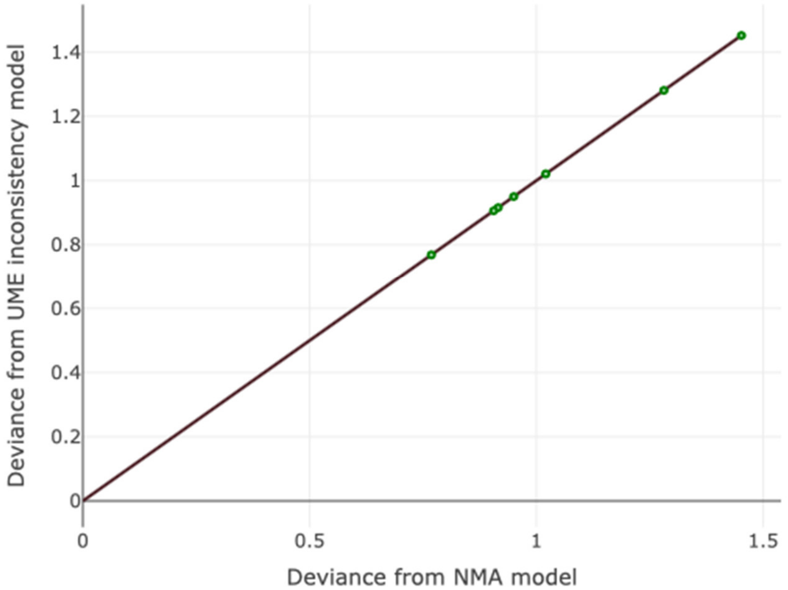

Supplementary Figure S149: Residual deviance from NMA model and UME inconsistency model for all studies.

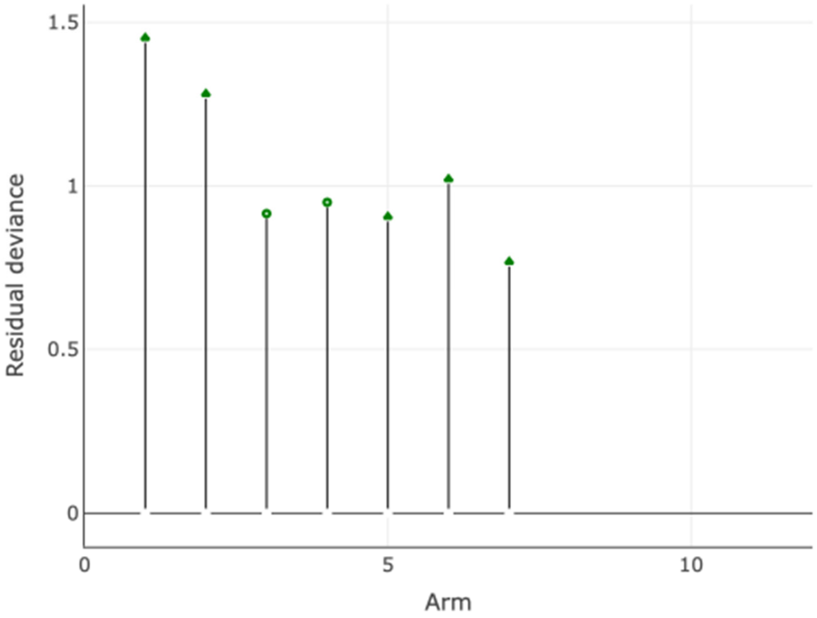

Supplementary Figure S150: Per-arm residual deviance for all studies. This stem plot represents the posterior residual deviance per study arm.

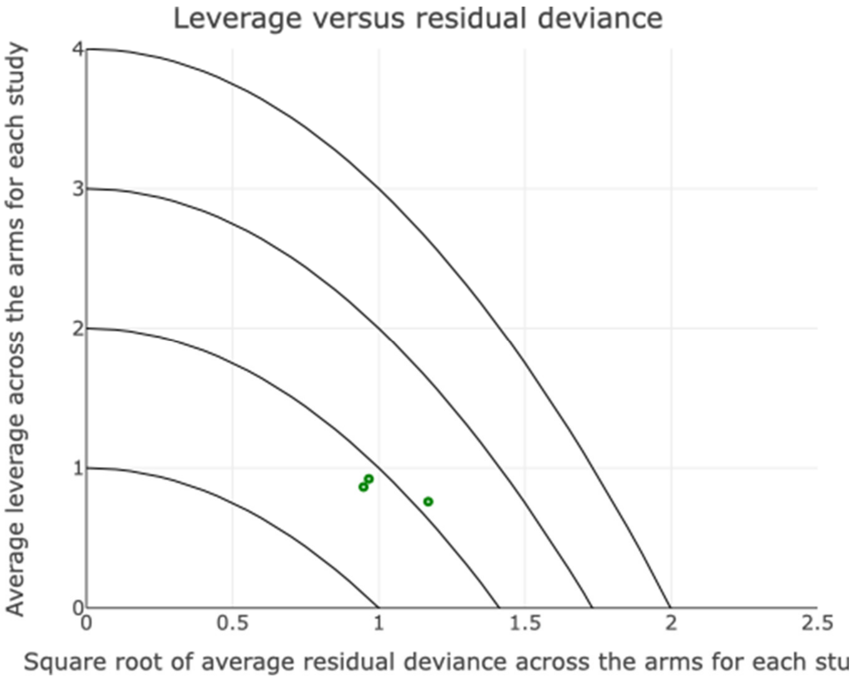

Supplementary Figure S151: Leverage plot for all studies.

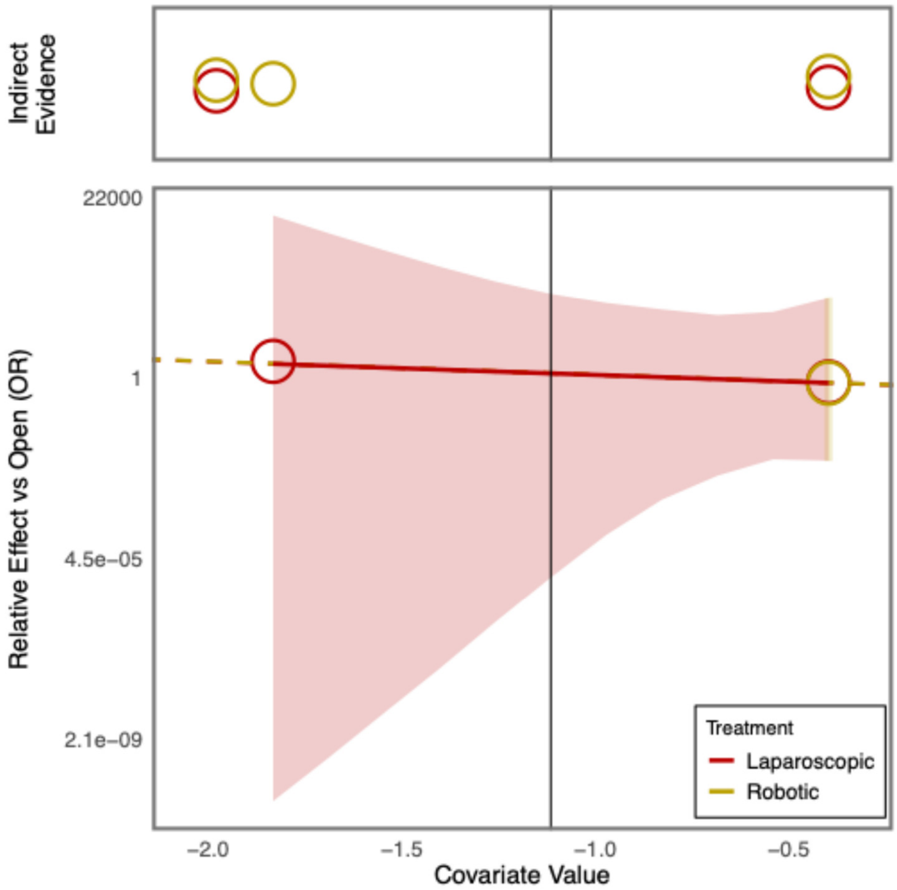

Supplementary Figure S152: Regression plot for the outcome 90-day major complications having as covariate the year of study publication.
